# School‐based interventions for preventing dating and relationship violence and gender‐based violence: A systematic review and synthesis of theories of change

**DOI:** 10.1002/rev3.3382

**Published:** 2022-12-15

**Authors:** Noreen Orr, Annah Chollet, Andrew J. Rizzo, Naomi Shaw, Caroline Farmer, Honor Young, Emma Rigby, Vashti Berry, Chris Bonell, G. J. Melendez‐Torres

**Affiliations:** ^1^ University of Exeter Medical School, University of Exeter Exeter UK; ^2^ University of Oxford Oxford UK; ^3^ College of Health and Human Performance University of Florida Gainesville Florida USA; ^4^ School of Social Sciences Cardiff University Cardiff UK; ^5^ Association for Young People's Health London UK; ^6^ London School of Hygiene and Tropical Medicine London UK

**Keywords:** dating relationship violence, gender‐based violence, schools, systematic review, theory synthesis

## Abstract

School‐based interventions for preventing dating and relationship violence (DRV) and gender‐based violence (GBV) are an important way of attempting to prevent and reduce the significant amount of DRV and GBV that occurs in schools. A theoretical understanding of how these interventions are likely to cause change is essential for developing and evaluating effectiveness, so developing an overarching theory of change for school‐based interventions to prevent DRV and GBV was the first step in our systematic review. Theoretical data were synthesised from 68 outcome evaluations using methods common to qualitative synthesis. Specifically, we used a meta‐ethnographic approach to develop a line‐of‐argument for an overarching theory of change and Markham and Aveyard's (2003, *Social Science & Medicine*, 56, 1209) theory of human functioning and school organisation as a framework for structuring the concepts. The overall theory of change generated was that by strengthening relationships between and among staff and students, between the classroom and the wider school, and between schools and communities, and by increasing students' sense of belonging with student‐centred learning opportunities, schools would encourage student commitment to the school and its values, prosocial behaviour and avoidance of violence and aggression. The theory of human functioning informed our understanding of the mechanisms of action but from our analysis we found that it required refinement to address the importance of context and student agency.

**Context and implications**

**Rationale for this study**

Despite numerous evaluations of school‐based interventions against dating and relationship violence and gender‐based violence, interventions are collectively poorly theorised.

**Why the new findings matter**

Our findings stress the importance of improving commitment to school, strengthening relationships between peers and with teachers, and acknowledging student agency. This transcends the limited psychological theories characterising many included interventions.

**Implications for practitioners and policy makers**

Interventions drawing strictly on psychological models of behaviour change may not be sufficient to address dating and relationship violence or gender‐based violence. However, prevention strategies that prioritise interactivity, relationship‐building and commitment to prosocial school norms may create the opportunities for students to develop positive behaviour change, with downstream implications for school cultures. We characterised school connectedness as a critical mediator, and noted that the perception and experience of safety in school spaces formed an often neglected, but theoretically salient, construct in understanding intervention effectiveness. New prevention strategies should start from a perspective of safety and connectedness rather than intra‐individual change.

## INTRODUCTION

Dating and relationship violence (DRV) refers to physical, sexual and emotional violence (including coercive control) in relationships between young people. This includes those aged under 16, who are not included in UK government definitions of domestic violence. Gender‐based violence (GBV) refers to violence rooted in gender equality and sexuality, such as harassment or bullying on the basis of gender or sexuality, sexual violence, coercion and assault including rape, within or outside dating relationships (Jewkes et al., 2015). Although DRV and GBV share common risk factors and mechanisms (Exner‐Cortens et al., 2013), they are rarely considered as joint constructs (Taquette & Monteiro, 2019). Common mechanisms include patriarchal and toxic gender norms at the societal level; inconsistent development and enforcement of violence prevention policies at the school level; and, at the individual level, exposure to and reinforcement of antisocial norms relating to gender, sexuality and violence (Barter & Stanley, 2016; Earnest & Brady, 2014; Taquette & Monteiro, 2019; Young et al., 2021). Norms accepting of GBV and harassment strongly correlate with DRV perpetration and victimisation, highlighting the importance of considering DRV and GBV jointly (Foshee et al., 2016; Jewkes et al., 2015; Miller et al., 2020; Reyes et al., 2016).

Adolescence is a crucial stage for focusing on the prevention of DRV and GBV and schools, as settings where young people are socialised into gender norms and where significant amounts of DRV and GBV occurs (Girlguiding, 2021; Ofsted, 2021), have the potential to promote gender‐equitable attitudes. The Department for Education's statutory requirement for Relationships and Sex Education (RSE) to be taught in all English secondary schools and Relationships Education in primary schools from 1 September 2020 (Department for Education, 2021) underlines the significance of schools as appropriate settings for prevention. The overall aim of RSE is that students understand the benefits of healthy relationships to their own mental well‐being.

DRV and GBV detrimentally impacts the health and well‐being of young people and, consequently, is an important public health issue. Existing evidence suggests that while boys and girls both experience major burdens of emotional and physical DRV, impacts are disproportionately experienced by girls. A cross‐sectional study based on a representative sample of 11–16 year olds in Wales found that 28% of girls report emotional victimisation and 12% report physical victimisation by a partner over the course of adolescence, while 20% of boys report emotional victimisation and 17% physical victimisation (Young et al., 2021). This study also found that age‐related trajectories in victimisation are steeper in girls than in boys, suggesting that adolescence is a critical period to arrest inequalities arising from DRV and GBV. Data analysed from the Youths' Romantic Relationships Survey in Canada found that girls were more likely than boys to report sexual dating violence victimisation (Théorêt et al., 2021), and girls were more vulnerable to experiencing the detrimental impacts of victimisation such as feelings of fear, distress and post‐traumatic stress compared to boys (Hérbert et al., 2017). Nationally representative data from the US Youth Risk Behavioural Surveillance Survey from 2001 to 2019 revealed that rates of forced sex were maintained for girls and decreased for boys, and as girls and boys aged, the risk of forced sex increased (Marcantonio et al., 2022). In Britain, the median age for most recent occurrence of sex against one's will, a form of GBV, is 18 among men and 16 among women (Macdowall et al., 2013). In addition, longitudinal evidence suggests that the onset of physical DRV and GBV peaks in mid‐adolescence, while onset of sexual DRV and GBV is greatest in late adolescence (Shorey et al., 2017).

The long‐term impacts of DRV and GBV are numerous. In adolescence, both perpetrators and victims report increased risky sexual behaviour, substance use and depressive symptoms (Barter & Stanley, 2016; Fellmeth et al., 2013; Johns et al., 2018; Shorey et al., 2015); in adulthood, survivors of DRV and GBV are more likely to be re‐victimised (Vivolo‐Kantor et al., 2016) and more likely to report poorer mental and physical health (Loxton et al., 2017). A systematic review of longitudinal studies found that both DRV and GBV experiences as adolescents were predictive of adult experiences of domestic violence (Costa et al., 2015), and earlier onset of intimate partner violence leads to greater impacts on mental and physical health in adulthood (Loxton et al., 2017). In addition, there are strong intersections with other inequalities; for example, DRV and GBV generates inequalities between heterosexual and cisgender young people and their sexual minority peers, such as substance misuse and increased burden of suicidal ideation arising from experiences of DRV and GBV (Johns et al., 2018; Mueller et al., 2015).

Many prevention programmes for DRV or GBV are delivered in schools. These interventions draw on a range of approaches ranging from ‘traditional’ classroom‐based instruction as part, or distinct from, RSE through to school‐level resourcing and restructuring. Didactically led programmes have been extensively evaluated. For example, Safe Dates included an RSE curriculum (Foshee et al., 1998); Second Step included classroom‐based social and emotional learning (Espelage, Low, Polanin, & Brown, 2015; Espelage, Low, Van Ryzin, & Polanin, 2015); and TakeCARE used a video‐based programme to teach bystander behaviour, or increased self‐efficacy to intervene, with the goal of reducing DRV and GBV (Sargent et al., 2017). Some of these had structural components; for example, Safe Dates increased services to adolescents in abusive relationships, and sought to upskill teachers and community service providers (Foshee et al., 1998). The Shifting Boundaries intervention, which compared a didactic and structural package against a structural‐only package (building‐based restraining orders, greater faculty and security staff in hot spots, school media campaign) and against no intervention, found reductions in sexual violence perpetration in the structural‐only intervention alone (Taylor et al., 2013). The mechanisms through which interventions may impact DRV and GBV outcomes are broad, including improved knowledge and self‐efficacy, improved reporting and bystander behaviours and better conflict resolution skills through to changes in school culture and responses (Espelage et al., 2016), and social norms at the group level (Pulerwitz et al., 2019).

There is no recent systematic review examining the evidence on the effectiveness of school‐based interventions for GBV; systematic reviews published since 2013 have focused on interventions for the prevention of DRV and have not considered intervention impacts with GBV (De La Rue et al., 2017; Fellmeth et al., 2013; Kettrey et al., 2019; Stanley et al., 2015). This is important because interventions nominally focusing on DRV may impact GBV and vice versa, underpinned by common mechanisms and structural features that lead to high rates of both in schools. As already noted, the shared mechanisms linking DRV and GBV constitute an important reason to consider these outcomes jointly. Some reviews (De Koker et al., 2014; De La Rue et al., 2017; Kettrey et al., 2019) have excluded important forms of GBV that may or may not occur in the context of dating relationships, such as unwanted sexting, coercive control and sexual harassment. Furthermore, some of these reviews (De Koker et al., 2014; Fellmeth et al., 2013; Kettrey et al., 2019; Lundgren & Amin, 2015) have included interventions across age ranges and settings rather than focusing on interventions in compulsory education settings specifically, which is most relevant to inform policy.

In England, schools could start using the RSE curriculum from September 2019 and research on the early adopter schools shows that challenges were encountered when developing and delivering the RSE curriculum (Department for Education, 2021). Children and young people were seldom positive about their RSE lessons and most felt the curriculum did not give them the information and advice they needed to navigate the reality of their lives (Ofsted, 2021). The early indications are that those schools seeking to implement the new RSE curriculum need further support to successfully deliver the curriculum and teaching on RSE topics (Department for Education, 2021). Thus, there is a good case for a new systematic review that provides usable information for practitioners and policy makers regarding the design and implementation of school‐based interventions for DRV and GBV prevention.

To our knowledge, there have been no systematic reviews of interventions for both DRV and GBV and so there is limited information on the effectiveness of these school‐based interventions and the characteristics most important for preventing DRB and GBV, the theories of change that drive them, and the factors that influence their implementation. As a good theoretical understanding of how an intervention is likely to cause change is essential for developing and evaluating effective interventions (Craig et al., 2008), the first step in our systematic review was to develop an overarching theory of change for school‐based interventions to prevent DRV and GBV. Synthesising theories of change for these interventions is important as it can lead to additional insights into the characteristics of effective interventions by uncovering underlying mechanisms (Baxter & Allmark, 2013) and provide the basis for refining and developing better programme theory (Michie & Prestwich, 2010).

## METHODS OF SYSTEMATIC REVIEW

A full protocol outlining the methods of this systematic review which followed the Preferred Reporting Items for Systematic Reviews and Meta‐Analyses (PRISMA) guidelines (Page et al., 2021) is available from (https://fundingawards.nihr.ac.uk/award/NIHR130144). The protocol was also registered with the PROSPERO registry of systematic reviews (CRD42020190463).

### Inclusion criteria

The following inclusion and exclusion criteria were used to determine eligibility of studies and inform the search for the literature.

#### Population

Children in compulsory education (e.g., aged 5 to 18 years) who are attending school.

#### Interventions

Interventions implemented in school contexts with students separate from, or as part of, RSE. These interventions could include one or more of:

- individual behavioural intervention (e.g., individual learning modules or apps);
- group or classroom‐based intervention or practices (e.g., as part of RSE; delivering DRV and GBV prevention content in other academic sessions (Tancred et al., 2019); delivery of content in groups during school hours);
- network‐based approaches, such as public opinion leader interventions;
- staff training and other service provision in schools (e.g., to recognise and respond better to sexual violence; Young et al., 2019); or
- local and school policy change (Bonell et al., 2018) to address structural factors relating to DRV or GBV, or to change school responses to DRV or GBV.

#### Control

Comparators could include business as usual, waitlist control or another active intervention.

#### Outcomes

Outcomes relating to the full scope of DRV and GBV behaviours. These included:

- DRV perpetration or victimisation, including physical violence; emotional violence, including isolation; coercive control, including internet‐mediated DRV; sexual assault in the context of relationships;
- GBV perpetration or victimisation, including harassment and bullying on the basis of gender or sexuality, including homophobic and transphobic bullying; internet‐mediated GBV, such as unwanted sexting or forwarding of sexts; unwanted sexual contact, such as groping; sexual assault; sexual harassment and rape; and
- knowledge and attitudes related to DRV and GBV, such as rape myth acceptance, bystander attitudes and GBV‐condoning norms.

Outcomes included self‐reported behaviours or experiences (e.g., were you groped in the last year; did you call someone names because of their sex or because you thought they were gay), teacher‐reported behaviours (e.g., how many times did you see students engaging in sexual harassment) or official reports (e.g., how many sexual harassment incidents were reported in the last year). Outcome measures were quantitative and included categorical, continuous or count measures. Measures could be composite items (a range of DRV behaviours collected as a count of behaviours) or could be behaviour‐specific. Behavioural outcomes could focus on: behaviours over a specific period; frequency (monthly, weekly or daily); the number of episodes of a behaviour; or an index constructed from multiple measures. Economic analyses could also include health‐related quality of life. Knowledge and attitude outcomes relating to gender or violence norms generally were excluded.

### Search strategy

In July 2020, we searched the following bibliographic databases from inception and without limitation on date or language:

- MEDLINE, Embase, PsycINFO, Social Policy and Practice (Ovid);
- CINAHL, ERIC, British Education Index, Education Research Complete, EconLit, Criminal Justice Abstracts (EBSCO*host*);
- Cochrane Database of Systematic Reviews (CDSR) and the Cochrane Central Register of Controlled Trials (CENTRAL);
- NHS Economic Evaluation Database (NHS EED via the Centre for Reviews and Dissemination);
- Social Science Citation Index and Conference Proceedings Citation Index (Web of Science, Clarivate Analytics);
- Australian Education Index, ProQuest Dissertations & Theses Global, Sociological Abstracts including Social Services Abstracts, Applied Social Sciences Index and Abstracts (ProQuest);
- Trials Register of Promoting Health Interventions (TRoPHI) and Bibliomap (EPPI‐Centre);
- Campbell Systematic Reviews (Campbell Collaboration).

The search strategy included both free‐text terms (i.e., searches in the title and abstract) and subject headings (e.g., MeSH in MEDLINE) for the school setting and DRV/GBV outcomes. In order to identify outcome, process and economic evaluations, we did not apply publication type or study design limitations. We updated the bibliographic database searches in June 2021, with a revised strategy developed to improve precision, and added further search terms for named interventions. Full strategies for the original and updated bibliographic database searches are available in Appendix S1.

We completed forwards and backwards citation chasing on included studies in Scopus (Elsevier), Web of Science (Clarivate Analytics) and Google Scholar, and reviewed the reference lists of relevant systematic reviews and reports. To identify linked studies and further grey literature, we conducted targeted searches in Web of Science and Scopus using first and last author names, and searched Google Scholar for specific intervention names (e.g., Project Respect; Shifting Boundaries). We also searched or browsed publication lists on key websites (including USAID: www.usaid.gov; the National Criminal Justice Reference Service: www.ncjrs.gov; and UNGEI: www.ungei.org).

### Study selection

Search results were downloaded into EndNote for deduplication. Subsequently, a single search file was uploaded to Covidence software. Two reviewers piloted the screening of successive batches of 100 titles/abstracts, meeting to discuss disagreements, calling on a third reviewer where necessary. Once 90% agreement was reached, each title and abstract was reviewed independently and in duplicate. Records retained after this stage were accessed in full text and assessed against the inclusion criteria in duplicate, and assigned to one or more evidence types (implementation/process, outcome, economic evaluation, mediation and moderation).

### Data extraction

Data were extracted using standardised forms by two reviewers independently and checked by a third reviewer. The data from the forms were entered into EPPI‐Reviewer 4. The full extraction details of the larger project included information on basic study details, study design and methods, outcome measures, relevant mediation and moderation analyses and economic data. For the theory synthesis, we focused on the descriptions of any theory of change, theoretical assumptions on how the interventions were expected to function, constructs, mechanisms and any contextual contingencies affecting these, as well as other theories cited.

### Data analysis

Drawing on methods used in previous theory syntheses (Bonell et al., 2013), this inductive analysis included a lines‐of‐argument synthesis (Noblit & Hare, 1988). Lines‐of‐argument synthesis is an appropriate method based on its understanding that each included study examines a ‘part of the whole’; that is, each study's account of theory of change represents one possible part of how school‐based interventions can work to reduce DRV and GBV broadly. Analysis was undertaken using thematic synthesis and line‐by‐line coding (Malpass et al., 2012).

Two reviewers began the theory synthesis with a subset of studies first to understand and agree a common approach, prioritising a selection of interventions that: (a) were multiply evaluated, and (b) represented the breadth of interventions. Subsequently, both reviewers proceeded through each set of studies, meeting to agree findings. The findings from each intervention (second‐order constructs) were then compared against each other using a ‘higher‐level’ iteration of these thematic grids to produce an overarching theory of change (third‐order constructs). Coding unfolded over several cycles, proceeding from initial coding to saturation, followed by confirmatory coding on remaining papers in the analysis.

## RESULTS

### Search results

**FIGURE 1:**
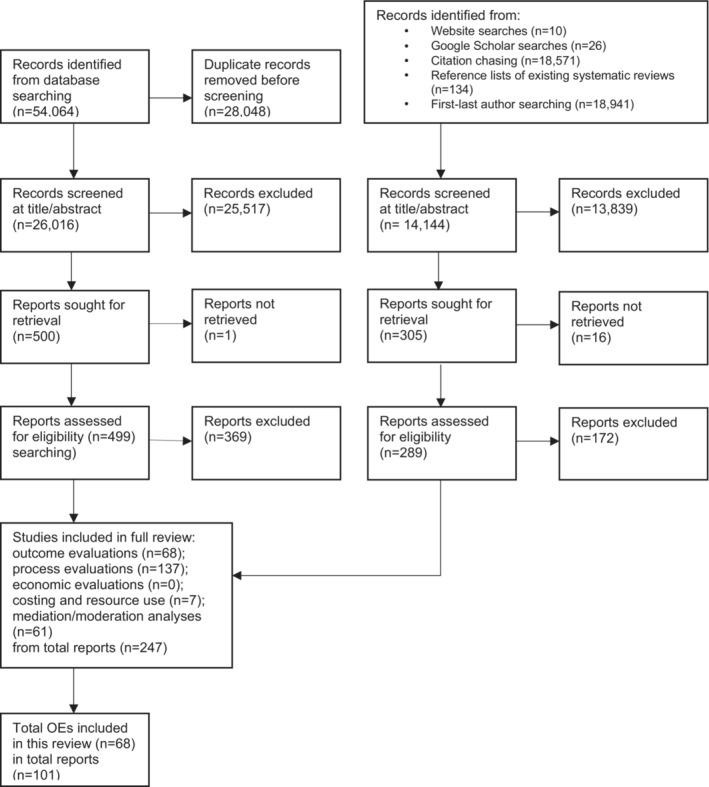
PRISMA

Searches identified 40,160 records after deduplication, of which 793 were screened in full text. Of these, 247 reports were identified as eligible for inclusion, and these were coded into 68 outcome evaluations, 137 process evaluations, and 7 costing and resource use studies (see Figure 1). Only outcome evaluations are included in this theory synthesis. A summary of intervention theories of change is presented in Appendix S2.

### Synthesis of theories of change

The synthesis of theories of change drew on different sets of included studies in cycles. After an initial coding round of five interventions described in ten reports, we undertook exploratory coding between two reviewers informed by template analysis (Brooks et al., 2015). We used a coding template with codes exchanged by two reviewers in a subset of interventions (*n* = 17) reported in two or more reports, focusing as well on breadth of intervention type. The template reflected the focus on programme theories and included intervention inputs, components, mechanisms, outcomes and key theoretical concepts (see Table 1). An example of the coding template as applied to interventions is provided in Appendix S3.

**TABLE 1:** Coding template

Themes	Codes and sub‐codes
*Inputs*	Curriculum, training of teachers, materials to deliver intervention
*Intervention goals*	Reduce bullying perpetration, peer victimisation, reduce sexual harassment victimisation and perpetration, increase bystander behaviour
*Key theoretical concepts*	Mid‐range theories, e.g., social learning theory, theory of planned behaviour, theory of reasoned action
*Mechanisms of change*	Knowledge, attitudes, skills, behaviours,
*Outcomes*	Reduce/prevent dating violence behaviours, reduce/prevent gender‐based violence behaviours

We then synthesised programme theories using thematic synthesis (Thomas & Harden, 2008). A subset of interventions that were reported in two or more papers were prioritised: we identified similarities, differences of emphasis and contradictions between multiple papers and developed summaries of the theories of change for the interventions, describing inputs, mechanisms of change, underlying theoretical assumptions, and proximal and distal outcomes. For the remaining interventions, summaries of the programme theories were produced. The analysis proceeded with another 37 reports analysed, at which point thematic synthesis yielded no new insights. The remaining included reports were analysed using a confirmatory coding approach.

The findings of the thematic synthesis were transformed using meta‐ethnography to develop a line‐of‐argument for an overarching theory of change. The key concepts identified during coding were treated as ‘first‐order constructs’ (Schütz et al., 1962), which we summarised as logic models and compared between each intervention (see Appendix S4 for examples). This generated second‐order constructs, which were our interpretations of the first‐order concepts. Third‐order constructs were then developed (based on key concepts and second‐order interpretations) to produce a line of argument that aimed to describe the overall mechanisms of change.

### Theory of human functioning and school organisation

To help in developing a line of argument, we used Markham and Aveyard's (2003) theory of human functioning and school organisation to promote student health, as it offered a framework for structuring the concepts. This middle‐range theory provided a ‘level of abstraction’ (Jagosh, 2019) that facilitated the interpretation of ‘what initially appeared to be disparate concepts’ (Tancred et al., 2018, p. 8). This theory proposes that schools can assist students to develop ‘essential human capacities’, two of which are ‘practical reasoning’ and ‘affiliation with other humans’. Practical reasoning is the ability to reason, critically reflect, and consider issues from a range of different perspectives. Affiliation is about concern for others and the ability to form relationships. Schools can play a ‘potentially profound’ role in helping students realise these capacities through their ‘instructional’ order, which relates to the way in which the school enables students to learn, and the ‘regulatory’ order, which is the way the school engenders a sense of belonging by encouraging students to internalise its values and adopt behavioural norms. The ideal situation for schools is when students are ‘committed’ to both the instructional and the regulatory orders, that is, being engaged with and able to meet the demands of the instructional order, and accepting of the norms of the regulatory order. Securing commitment from students depends on the ‘classification’, which relates to the school's institutional ‘boundaries’ and how rigidly they are set, for example, in the relationships between teachers and students; and ‘framing’, which relates to the degree to which a school's communication and pedagogic practices are student‐centred. In short, this theory proposes that by ‘weakening’ the classification and framing, schools can secure the commitment of students who have ‘the best chance of functioning well and maximising their health potential as adults’ (Markham & Aveyard, 2003, p. 1214).

### Intervention theory of change

**FIGURE 2:**
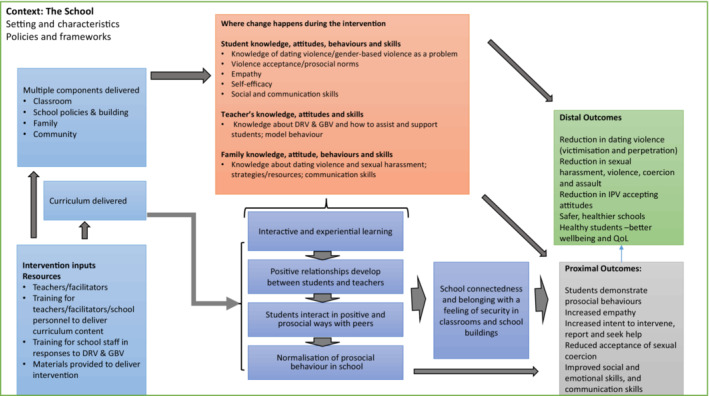
Logic model presenting the overarching theory of change for the school‐based interventions

The logic model (Figure 2) depicts the overarching theory of change for the school‐based interventions. Following a systems thinking approach, it presents schools as ‘bounded ecological systems’ (Moore et al., 2019, p. 26) into which these interventions are introduced. A systems perspective recognises that schools comprise different sub‐systems such as year‐groups and form‐groups, and are nested within bigger systems such as national education systems, the local community and other ‘parallel systems’, such as families (Keshavarz et al., 2010, p. 1469). Important interactions can occur within and between these systems. We used the theory of human functioning and school organisation with its focus on institutional processes to theorise how interventions can modify institutional boundaries and improve the connections between the various systems. After describing the starting theoretical propositions, we present the theory of change for the interventions and consider intervention inputs, intervention mechanisms of change and intervention outcomes.

Drawing on Markham and Aveyard's (2003) theory, we developed two starting hypotheses, broadly summarised as that school‐based interventions designed to reduce participation in DRV and GBV have the greatest potential to be effective when based on the principles of weakening classification and framing (Markham & Aveyard, 2003). First, we hypothesised that interventions aimed to weaken the institutional boundaries between the school and surrounding communities and strengthen relationships within the school. For example, a number of the curriculum‐based interventions had additional components directed at multiple levels such as the community and family/parents to support and reinforce change initiated in the classroom. Many of the interventions were delivered by teachers, and some in collaboration with students, which opened up opportunities for increased interaction and positive relations between teachers and students. In some cases, teachers and other school staff were trained to model positive behaviours to reinforce behaviour change among the students. Secondly, we hypothesised that interventions aimed to involve students in their learning and in school‐level decisions and increase belonging, thereby weakening the framing of the schools. For example, many of the interventions utilised student‐centred techniques such as group work and role‐playing to encourage interactive and experiential learning with student input to their own learning, which in turn, promoted positive interactions and relations between students and teachers and among the students themselves. This facilitated students' capacity for practical reasoning and critical reflection.

## INTERVENTION INPUTS

The majority of the interventions involved classroom curriculum components (Figure 2, light blue box) that could be delivered within the school timetable or ‘after school’ (Decker et al., 2018; Gage et al., 2016; Mathews et al., 2016). Some interventions had additional components targeting multiple levels: for example, the Safe Dates intervention had an out‐of‐school component with training of community service providers and a weekly support group for victims of dating violence (Foshee et al., 1998); and the JOVEN intervention had six group sessions for students and two for parents, with parents joining students in the last session to practise healthy communication skills in negotiating ‘curfews’ and dating (Gonzalez‐Guarda et al., 2015). Others can be described as multi‐component or multi‐level interventions such as the Expect Respect project which

attempted to take a whole school approach to preventing bullying and sexual harassment by preparing *all members of the school community* to recognize and respond effectively to bullying and sexual harassment. The Expect Respect Project utilized five program components: a 12‐week classroom curriculum for students, trainings for staff, education and support for parents, encouragement and guidance to administrators for policy development, and support services for students who had been affected by bullying, sexual harassment, or sexual or domestic violence. (Whitaker et al., 2003, p. 330; reviewer's emphasis)

Many of the interventions involved training teachers to be able to deliver curriculum content (Figure 2, light blue box), while others did not require teacher input as the curriculum was delivered by external facilitators. External facilitators were often very experienced in prevention of GBV and DRV (Muck et al., 2018) and could also receive extensive training: for example, the rape crisis educators who delivered the Green Dot curriculum had a 4‐day in‐depth training and received individualised feedback throughout the trial (Coker et al., 2019). In another example, the ‘instructors’ delivering the curriculum for the IMPower and 50:50 intervention were recruited through an ‘intensive process’ that ensured that they were passionate about preventing sexual violence and were respected members of their communities. They

received extensive instruction by expert facilitators and participated in mock interviews and field‐training exercises… Trainers were required to pass a rigorous examination consisting of a written test, oral examination, and physical skills demonstration before becoming paid employees teaching the curriculum at intervention sites. New trainers were supervised by a more experienced trainer for the first year of teaching. (Baiocchi et al., 2017, pp. 819–820)

There were those interventions that used both teachers and external facilitators such as the *Benzies & Batchies* intervention, with teachers delivering the introductory and last classes and trained social‐skills instructors delivering three lessons on student skills (de Lijster et al., 2016). In some cases, teachers and other school staff also received training to enable them to be positive role models for students; for example, in the BITB‐HSC intervention teachers and other school personnel were trained ‘to be positive bystanders in situations of adolescent interpersonal violence’ (Edwards et al., 2019, p. 489). A number of interventions did not require teachers or facilitators for implementation such as the online programme ‘Teen Choices’ (Levesque et al., 2016). It was cited as an advantage that it used computers and expert system technology to deliver the programme ‘efficiently’ and ‘cost‐effectively’ when compared to programmes that required significant teacher training or input from professional educators.

The focus of the curricula varied according to the goals of the interventions such as reductions in dating violence, reductions in gender‐based violence, increased bystander behaviour, and reduced acceptance of sexual coercion. Topics and skills covered varied in terms of depth from those that had a specific focus such as the legal aspects of dating violence in the three class period Ending Violence intervention (Jaycox, McCaffrey, Eiseman, et al., 2006; Jaycox, McCaffrey, Weidmer Ocampo, et al., 2006), to those that had a broader focus such as the social–emotional learning programme, Second Step, which was delivered in 41 lessons over 3 years (Espelage et al., 2013; Espelage, Low, Polanin, & Brown, 2015; Espelage, Low, Van Ryzin, & Polanin, 2015). Some of the interventions could best be described as ‘add‐ons’ such as the Katie Brown Educational Program (KBEP), a ‘freestanding’ DRV programme that could be incorporated into any school curriculum by replacing five health class periods during one week (Joppa et al., 2016). Other interventions integrated lessons into the existing school curriculum, often within health, physical education, science and social studies classes (Avery‐Leaf et al., 1997; Bando et al., 2019; Coyle et al., 2019; Jaycox, McCaffrey, Eiseman, et al., 2006; Peskin et al., 2019; Roberts, 2009). In some cases, integrating interventions into existing curriculum requirements was an attempt to weave together violence‐related and broader learning. For example, the Fourth R intervention integrated dating violence prevention with lessons on healthy relationships, sexual health and substance use; these topics were not addressed directly; rather, healthy, non‐violent relationship skills were an underlying theme that was ‘woven’ throughout the curriculum. For the authors, the advantages of integration were clear:

[t]he focus on embedding the program into curriculum that meets the guidelines for mandatory classes in high schools provides a vehicle for widespread dissemination and sustainability far beyond that which can be achieved by add‐on programs. (Wolfe et al., 2009, p. 698)

## MECHANISMS OF CHANGE

In Figure 2, we have depicted mechanisms as lower and higher level: the lower level mechanisms are where individuals react or respond to the resources provided by the interventions that trigger change (for example, the knowledge gained from lessons about sexual violence); and higher level mechanisms are where mechanisms interact with one another and evolve within the school system of relationships (for example, students interacting in positive and prosocial ways with peers).

Two key themes were generated from the theory synthesis mechanisms leading to student commitment to prosocial values and behaviours:

1. strengthening relationships between the school and surrounding communities, and within the school (between students and teachers, and between students);
2. increasing belonging in the schools by student‐centred approaches, would lead to student commitment to prosocial values and behaviours.

The higher level mechanisms of change through which this happens are depicted in the logic model. These occur through interactive and experiential learning, positive and prosocial student interaction with peers, positive relationships between students and teachers, normalisation of pro‐social behaviour and increased school connectedness (Figure 2, dark blue boxes). Lower‐level mechanisms facilitating change occur through the changes in student, teacher and family knowledge, attitudes, behaviours and skills (Figure 2, pink/orange box). These are discussed below through the broader themes of ‘strengthening relationships’ and ‘increasing belonging’.

## STRENGTHENING RELATIONSHIPS BETWEEN SCHOOL AND THE OUTSIDE WORLD

### School and community

Some multilevel interventions included community components and while these did not explicitly theorise a strengthening of school‐community relations, they were conceived as influencing students' beliefs about the need for help and where it could be sought within their communities. Some interventions provided training for community service providers such as Safe Dates, which offered 20 training workshops to service providers in the community. Other interventions, such as Expect Respect, aimed to help students and families link in with community resources and/or co‐ordinated with local free counselling services (PPM‐Based Intervention for Domestic Violence) (Ekhtiari et al., 2013, 2014). In the latter case, introducing the student to free community services was considered as an ‘enabling’ factor that would facilitate change by providing a resource that was considered ‘essential’ for the behaviour change (Ekhtiari et al., 2013, p. 23).

For Markham and Aveyard (2003, p. 1217), schools encourage students to become ‘committed’ by promoting ‘cultural congruence between the school and the wider community’. However, pursuing ‘cultural congruence’ by strengthening relationships with the community may not be an option in some schools, particularly where the values of the school and community diverge widely. Arguably, in those communities where students experience a lack of community safety and are exposed to many forms of violence, it is conceivable that schools could seek to raise their institutional boundaries with the community. By problematising and addressing DRV and GBV, schools are differentiating themselves from community norms supportive of such violence. Some interventions were implemented in settings such as these and as illustrated by Decker et al.'s (2018, p. 2) discussion of sexual violence in Malawi, where the social norms that tolerate and expect silence and compliance, especially from young women, have resulted in sexual violence being endemic. Bando et al. (2019, p. 226) highlighted the significance of context for programmes addressing social norms and the ‘extremely high rates’ of violence in El Salvador, which meant that all of the ECPVG intervention activities had to take place within the school because activities outside school presented security risks.

### School and parents

As already noted, some multicomponent interventions had family or parent components: some informed about the intervention via educational booklets, newsletters and presentations, but also linked parents to resources and included topic information, tips and activities (Macgowan, 1997). For example, the IYG intervention's parent component (Peskin et al., 2019), comprised two parent newsletters. These covered definitions of dating violence, signs of unhealthy relationships, strategies for enhancing parent–child communication, and information on online safety and resources. In addition, there was also parent–child homework activities (Peskin et al., 2019) which aimed to promote parent–child communication on dating expectations and relationships, and on healthy and unhealthy relationships. Some interventions provided training specifically for parents. For example, in the Dating Matters intervention, there were different training programmes for parents according to student grade:

Each parenting program taught participants skills for positive parenting and communicating effectively with their children about healthy relationships. (Niolon et al., 2019, p. 16)

The JOVEN intervention was offered to student‐parent dyads and parents received two group sessions focusing on effective parenting and communication. The content also addressed prosocial behaviour relating to dating violence, and understanding and working with the school system. Similarly, the Skhokho intervention had a 4‐day workshop held at weekends for parents and their children to strengthen their relations and promote better communication (Jewkes et al., 2019).

The family components of interventions were theorised as strengthening the relationships between schools and parents, and aligning norms within the family to that of the school and thereby supporting and reinforcing the change instigated by the intervention. This is highlighted by Ekhtiari et al. (2014, p. 989):

Educational booklets were distributed among parents to involve them, especially mothers, in violence prevention education to their daughters and *reinforce messages* learned at the school. (reviewer's emphasis)

## STRENGTHENING RELATIONSHIPS ACROSS THE SCHOOL BEYOND THE CLASSROOM

Some interventions aimed to strengthen relationships across the school beyond the classroom; this was particularly evident in the multicomponent, multi‐level interventions such as Expect Respect, which aimed to have strategies to build a consistent response at the individual, classroom and school‐wide levels for responding effectively to bullying and sexual harassment. In addition to training the teachers delivering the curriculum and counsellors, training was provided once a term for *all* school personnel, including bus drivers, cafeteria workers, hall monitors and office staff (Rosenbluth et al., 2004; Whitaker et al., 2003). Preparing all school staff to respond effectively to witnessed or reported incidents of bullying and sexual harassment supported the aim of the Bullyproof curriculum in reducing the social acceptance of bullying and sexual harassment. In another example, school personnel received two sessions in the JOVEN intervention on mentoring youth on relationships—this included a focus on recognising teen dating violence and promoting prosocial behaviours in the context of relationships (Gonzalez‐Guarda et al., 2015). As Espelage et al. (2015, p. 477) observed:

lessons need to be reinforced outside of the classroom and outside of the lesson. All school staff need to be reinforcing the content of these programs.

Involving all school staff in interventions to bring about change worked towards strengthening the relations between different professional roles so that all were focused on student well‐being. Arguably for students, it also increased the connection between their academic learning and broader development. Some interventions, for example, Expect Respect, also targeted school policies and encouraged school principals and administrators to develop a school policy to ensure consistency in staff responses to incidents and reports of bullying and sexual harassment. School principals were urged to create a policy document with input from staff and present it to school staff, students and parents (Rosenbluth et al., 2004; Whitaker et al., 2003). However, there did not seem to be an opportunity for students to contribute to the creation of school policy documents, rather than passively ‘receive’ these policies.

Other interventions targeted change at the physical infrastructure of the school environment with the aim of limiting places that were conducive to violent behaviours such as bullying. In these interventions, the process of how this was achieved—consulting and listening to students—was possibly as important as the outcome—a safe physical space. In the multilevel Shifting Boundaries intervention, the classroom intervention was matched by a building‐based intervention that involved building‐based restraining orders, higher levels of security presence in safe/unsafe ‘hotspots’, and placement of posters in school buildings to increase awareness and reporting of dating violence to school personnel (Taylor et al., 2011, 2013). Schools were involved in working with their students to identify the unsafe areas through ‘hotspot mapping’, which enabled students to communicate where potential danger spots were located in the school building.

Similarly, another intervention called PREPARE engaged students in the development of a school safety programme via a photovoice programme (Mathews et al., 2016). Its aim was to ‘*empower students to be the driving force* in improving physical, emotional and sexual safety at school and to influence school safety policy’ (Mathews et al., 2016, p. 1824; reviewer's emphasis), and students mapped and photographed unsafe situations and places in the school. Arguably, involving students outside the classroom in issues such as school safety presented students with opportunities to contribute to school policy and have a voice in the school's decision‐making process. This, in turn, could give both students and staff insights into ‘each other's realities’ (Markham & Aveyard, 2003, p. 1216), and potentially enhance students' attachment or connectedness to the school. It was also an opportunity for the school to reframe some of its decision‐making to focus more on student perceptions.

## STRENGTHENING RELATIONSHIPS BETWEEN TEACHERS AND STUDENTS

Teachers were key to implementing many of the interventions with opportunities to improve the quality of their relationships and strengthen bonds with their students. This had the potential to increase student connectedness to teachers and student commitment to the school and its values.

Some of the interventions dealt with changing teachers' violent behaviours towards their students in order to achieve a safer learning environment. Arguably, reducing the threat and fear of violence is essential for improving teacher‐student interactions and relations. Teachers received training as part of an intervention in order to change their attitudes, beliefs, social norms and behaviour. In some cases, the school itself could be the main location where students were exposed to violence (Devries et al., 2017; Jewkes et al., 2019) and in South Africa, schools are often the places where students feel least safe (Jewkes et al., 2019, p. 3). Part of this can be explained by teachers using physical, sexual and emotional violence against students. Interventions that aimed to reduce violence from teachers to students clearly have potential to improve teacher‐student relations and change a school culture of tolerance for violence. The Skhokho intervention trained teachers in positive discipline and classroom management (Jewkes et al., 2019), and the Good School Toolkit also focused on improving teaching techniques, and training in non‐violent methods of discipline, as part of a change of ‘operational culture at the school level’ (Devries et al., 2017). In this context, strengthening the relationships between teachers and students was fundamentally one about changing the quality of the relationships, with teachers being held accountable for respecting students' rights not to be assaulted and having a better understanding of power relations.

Teachers also received training in some interventions to enable them to model prosocial behaviours and act as positive role models for students. This extended the teacher's role from a focus on academic education to include emotional education and arguably could impact the teacher–student relationship. It may not necessarily have helped strengthen relations between teachers and students but positive role‐modelling by supportive teachers may have acted as a positive reinforcement for those students following prosocial behaviours. In the Coaching Boys into Men (CBIM) intervention, the coaches served as positive role models for the male athletes and in particular, modelled the bystander intervention skills of speaking up and intervening on witnessing harmful and disrespectful behaviours. Using coaches in this intervention was based on the recognition of their ‘role as influential, nonparental role models render[ing] them uniquely posed to positively impact how young men think and behave’ (Miller et al., 2012, p. 432).

Many of the interventions were dependent on teachers delivering the specific intervention curricula, which then opened up opportunities for positive interactions between teachers and students. In programmes such as Second Step, which was delivered by teachers weekly or semi‐weekly over a period of 2 years, teachers could develop sustained and supportive relationships with students that could ‘promote greater understanding of each other's values’ (Markham & Aveyard, 2003, p. 1216). Espelage et al. (2015, p. 466) observed that positive teacher‐student relations were promoted in the Second Step intervention, with teachers facilitating dyadic and group discussions that ‘provide opportunities to promote positive teacher‐student interactions which might not occur when the majority of instructional time is spent on only academics’.

This is considered in more detail in the discussion below of increasing belonging. Other interventions that involved students in aspects of the delivery of the intervention could potentially open up opportunities for positive relations between students and teachers by giving students a greater insight into the teachers' realities. For example, in the Safe Dates intervention (Gage et al., 2016), students were trained to lead peer support sessions, complementing the work of the teachers; in the Dat‐e Adolescence intervention (Munoz‐Fernandez et al., 2019), two students implemented the final two sessions of a seven‐session programme; and in the Good School Toolkit, activities at each school were delivered by two staff and two student ‘protagonists’ (Devries et al., 2017). Where teacher and student relationships became more collaborative, so the distinction between teacher and student roles became more blurred. Furthermore, increasing student involvement in the curriculum, as illustrated by these examples, gave students a voice and input into their own learning. Opportunities to engage like this may also have increased student affiliation with the school and its values.

## STRENGTHENING RELATIONSHIPS BETWEEN STUDENTS

A strong sub‐theme of strengthening relationships was change aimed at encouraging better communication and cooperation, and positive peer bonding between students. Many of the interventions were focused on the nature of healthy and unhealthy relationships in the context of both dating and friendship and aimed to show students how to have clearer rules for their interactions and establish appropriate boundaries with one another. This concept of ‘boundaries’ in personal relationships, as norms guiding behaviours, was a recurring theme within the interventions and focused on provoking students to consider their relations with others. Recognising and setting boundaries (e.g., naming harmful behaviours and warning about consequences) was central to the IMPower intervention (Baiocchi et al., 2017; Decker et al., 2018). The Shifting Boundaries intervention (Taylor et al., 2008), had an ‘interaction‐based curriculum’ which addressed the setting and communication of boundaries within relationships, forming healthy relationships and friendships (and the continuum of friendship and intimacy), and wanted/unwanted behaviours within relationships. The importance of setting personal limits and respecting others' limits in relationships were covered in various interventions such as It's Your Game…Keeping It Real (Peskin et al., 2014), Me & You (Peskin et al., 2019), the Katie Brown Educational Program (Joppa et al., 2016), Teen Choices (Levesque et al., 2016), Let Us Protect Our Future (Jemmott 3rd et al., 2018) and You‐Me‐Us (Coyle et al., 2019). Intervention curricula also considered the negative consequences of unhealthy relationships (Foshee et al., 1998; Taylor et al., 2008) and the legal penalties of perpetuating sexual violence (Jaycox, McCaffrey, Eiseman, et al., 2006; Taylor et al., 2008). Recognising and respecting personal limits in their personal and peer relations is arguably fundamental to improving student relationships, increasing respect and encouraging prosocial behaviours. Bystander interventions also targeted changing the relationships between students and aimed to

change the peer context, removing the bystander support that is such a critical driver of bullying and other violent behaviours’ (Espelage et al., 2015, p. 54).

Bystander interventions aim to change the norms that foster violence acceptance and sought to increase student knowledge of the range of violent behaviours and how to distinguish between, for example, sexual harassment and flirting. They also sought to increase student empathy for the victim and self‐efficacy to use appropriate bystander responses when students encountered situations and behaviours that could lead to violence (e.g., Second Step and Expect Respect).

The Green Dot bystander intervention illustrated how students themselves could play an important role in normalising prosocial behaviour and norms across student groups. Student leaders—those who were respected and emulated—were selected to receive intensive bystander training on the basis that they would diffuse prosocial behaviour and norms to other students through peer networks.

…changes in norms can be diffused rapidly within social networks through peer pressures. (Coker et al., 2019, p. 154)

There were other interventions that recognised the role of students in diffusing behaviours to other students. For example, there was potential for ‘peer‐to‐peer diffusion’ through the peer mentoring and role‐modelling that was part of the Fourth R curriculum (Cissner & Ayoub, 2014; Wolfe et al., 2009). In a similar vein, the *Benzies & Batchies* intervention used peer‐educators to perform a play and lead a group discussion to influence students' perceptions of other people's behaviour (de Lijster et al., 2016).

## INCREASING BELONGING WITH STUDENT‐CENTRED LEARNING

The second important theme related to mechanisms of actions was that interventions sought to increase students' sense of belonging by involving them in the learning process using interactive and experiential learning, which encouraged a ‘student‐centred’ framing of teaching (Markham & Aveyard, 2003). However, enabling students to input to their own learning did not detract from the important role of the teacher in delivering the curricula; teachers received training in the curriculum specific to the intervention and could also receive resources to support their delivery. For example, in the Fourth R intervention, teachers received detailed lesson plans, handouts for lessons and exercises; but there was some scope for teachers to choose activities and exercises they preferred. Training sessions explored how teachers could adapt materials, indicating that they were empowered to make lessons relevant to context. In the Shifting Boundaries intervention, Taylor et al. (2008) reports that teachers were involved in the curriculum design with their ideas incorporated into the lessons, such as varying the pedagogy to avoid relying solely on didactic pedagogy. Thus, while the curriculum for a particular intervention was designed to be delivered by teachers and ‘teacher‐led’, this did not preclude student interactive and experiential learning.

Interactive learning was achieved with a variety of techniques such as discussions, activities, exercises, worksheets and games, all offering varying degrees of student participation. An emphasis on student interaction in the Second Step intervention was highlighted by Espelage et al. (2013, p. 181):

Lessons are highly interactive, incorporating small‐group discussions and activities, dyadic exercises, whole‐class instruction, and individual work;

and in the GEMS programme by Achyut et al. (2011, p. 11):

the GEA [group education activities] use participatory methodologies such as role play, games, debates and discussions to engage students in meaningful and relevant interactions and reflection about key issues.

Interactive media, videos, decision‐making games, puppet shows, films, music and art projects also featured and supported curricula content. Elements such as plays performed by students and posters, with poster contests (Safe Dates, *Benzies & Batchies*), and social marketing campaigns (ECPVG; Bando et al., 2019) to enhance learning, were ways of enabling students to use their own knowledge and skills in the learning process. Through learning to work with each other, students also had opportunities to interact in positive and prosocial ways and improve their social and communication skills. This, in turn, contributed to strengthening the relationships between students by reinforcing those aspects that aimed to alter relationships between students.

Student acquisition of skills was important for many of the interventions and different interventions had different foci—relationship skills, problem‐solving skills, assertive skills, conflict management resolution skills, and bystander skills—to intervene safely and effectively. Lessons could incorporate skill‐based exercises and video demonstration of skills, designed to increase skill acquisition and to enable students to practise new skills. The use of group and collaborative work also encouraged students to practise skills in a supportive environment. In some interventions—such as Expect Respect, Second Step and Fourth R—there was extensive use of scenarios and role‐playing; for example, the bystander components of these interventions aimed to not only educate students on their responsibility as bystanders, but to enable them to practise the skills of intervening to help others and stop bullying behaviours.

Role‐playing also encouraged students to take the perspective of others and engender empathy for students victimised by bullying and violent behaviour. Understanding the perspective of others was also helped by reflection opportunities and ‘journaling activities’ being part of the learning process (Peskin et al., 2014). Facilitating student understanding of ‘multiple realities’ was embedded in the curricula of many interventions, as highlighted by Taylor et al. (2008, p. 10) in relation to Shifting Boundaries:

[t]he lessons…did not provide simple answers, or in some cases the answers at all, but rather made students struggle with subjectivity…and ambiguity.

Student‐centred approaches had the potential to engage students in the learning process and develop their ‘self‐reflective skills’ ‘to identify and understand the origins of their own and their classmates' orientations to meaning, values, interests and expectations’ (Markham & Aveyard, 2003, p. 1217).

There were a number of interventions such as TakeCARE (video), Jesse (prosocial video game) and the CD‐ROM Educational Program on Sexual Knowledge that purportedly used technology to engage students to increase bystander behaviour, increase empathy for female victims of intimate partner violence, and increase knowledge and attitudes towards sexual violence (Boduszek et al., 2019; Jouriles et al., 2019; Sargent et al., 2017; Yom & Lee, 2005). The videos demonstrated helpful bystander responses and how to use healthy relationship skills, while the JESSE intervention allowed the student to role‐play a variety of characters in the game, and so could be either experiencing or perpetrating physical and emotional violence. However, in these interventions, there was no provision for students to interact with each other. Arguably, embedding these interventions in a context of active learning by using, for example, structured discussions facilitated by teachers and associated exercises and assignments, would have increased student engagement. The MVMC intervention (Rowe et al., 2015) is an example where technology, in the form of an immersive virtual environment (IVE), was combined with input from facilitators who modelled and demonstrated assertive resistance skills through role‐play. The aim was that girls should have ‘multiple’ opportunities to practise skills and receive feedback on use of skills: each participant completed three virtual simulations in which the verbal sexual coercion increased in severity and after each they received feedback from the facilitator and other participants, with the possibility of repeating sessions until the participant demonstrated assertive resistance.

Where technology was used within an intervention, authors often claimed that it enhanced interactivity and enjoyment (Yom & Lee, 2005), enabled immediate feedback, and a ‘tailored’ experience. For example, in the Me & You intervention (Peskin et al., 2019), some of the computer activities such as the quizzes provided tailored answers depending on students' answers. Levesque et al. (2016) have argued that one of the problems with existing interventions is that they are ‘one‐size‐fits‐all’ and neglect important factors such as differences in dating history and history of dating violence victimisation and perpetration. Their Teen Choices intervention was an online programme that provided assessments and individualised guidance matched to dating history, dating violence experiences, and stages of readiness for using healthy relationship skills. It had five intervention tracks to meet the unique needs of (a) high‐risk victims, (b) high‐risk daters, (c) low‐risk daters, (d) high‐risk non‐daters, and (e) low‐risk daters. Clearly, tailored feedback to students according to dating status and risk level is responsive to student‐needs but, arguably, there was scope to enhance Teen Choices further, as acknowledged by Levesque et al. (2016), with teachers facilitating class discussions and student activities to encourage active learning.

## OUTCOMES

Most interventions aimed to reduce violence: some focused on dating and relationship violence victimisation and perpetration, some on gender‐based violence, and some on both. For many of the interventions, proximal outcomes (Figure 2, light grey box) led to reduced dating‐based violence and gender‐based violence through the development of prosocial skills, increased empathy, increased intent to intervene, report and seek help, and increased social and communication skills. These changes were realised through the mechanisms discussed in the previous section.

## DISCUSSION

The interventions within this synthesis aimed to reduce DRV and GBV by strengthening relationships within schools, between and among students and staff, between the classroom and the wider school, and between schools and communities, and by increasing belonging with student‐centred learning opportunities. Many of the interventions were delivered by teachers, and some with student involvement, which encouraged greater interaction and collaborative, supportive relationships. A strong focus of the interventions was to change the relationships between students, helping them to set their personal limits and respect others' limits within relationships. Many of the interventions featured interactive and experiential learning enabling student‐centred teaching and engagement with students and teachers. Some interventions had additional components to reach beyond the classroom, such as the whole school and families. Through this set of complex mechanisms, interventions aimed to provide students with knowledge and skills, developed in a context of interactive and supportive learning, promote pro‐social attitudes and values to underpin behaviour change, and encourage a sense of security and connection to the school.

The theory of human functioning and school organisation (Markham & Aveyard, 2003) informed the overarching theory of change: the theory suggests that students will engage in prosocial behaviours when they feel committed to school and its values, while uncommitted students are more likely to engage in anti‐social behaviours such as violence and aggression. The theory also proposes that student commitment to school is achieved by those schools who have policies and practices that are student‐centred and strengthen relationships between teachers and students, the students themselves, and between schools and local communities. The theory of human functioning has been described as a ‘complex, nuanced sociological theory’ that addresses the deeper structural influences on young people's behaviour (Moore & Evans, 2017, p. 133), suggesting that it was a pertinent choice for developing an overarching theory of change and enabling a deeper consideration of underlying mechanisms. Their theory resonates with the WHO definition of health promoting schools (WHO, 1997) and also with ‘ecological systems’ approaches (Hawe et al., 2009), both of which suggest the importance of the school environment (situated within a broader social context) as a setting in which students participate and interact with other settings such as family and community, for promoting student health. Using Markham and Aveyard's (2003) theory helped inform our understanding of the change mechanisms underlying the interventions included in the review.

However, rather than using Markham and Aveyard's language—‘weakening’ the classification (i.e., institutional boundaries) and the framing (i.e., communication and pedagogic practices)—we reframed these and presented them as ‘strengthening relationships’ and ‘increasing belonging’. The concept of boundaries is key to Markham and Aveyard's discussion of classification but in our analysis we found that we needed to be clear on the definition of boundaries, particularly as we examined boundaries in the sense of norms guiding student behaviours. For example, many interventions were concerned with helping young people create and strengthen healthy boundaries with their peers and partners. To use ‘weaken’ in this context was confusing. Additionally, we found that while some school boundaries should be weakened, there were other school boundaries that might promote prosocial norms and behaviours and should be maintained or strengthened. A beneficial school and community boundary could be the gender‐equitable norms and prosocial behaviour promoted by the school in contrast to the local culture of the community, where anti‐social and violent norms abound. So the notion of weakening the boundary between the school and local community may work in many Westernised contexts, but less well in other cultural contexts such as South Africa and El Salvador considered in this synthesis. This reinforces the importance of how mechanisms work are contingent on context and that what works in one country or community setting may not work elsewhere. There were examples of interventions that reached beyond the boundaries of the school to engage with local communities in order to encourage the ‘convergence of values, beliefs, and interests of the school and the wider community’ (Markham & Aveyard, 2003, p. 1216). Arguably, to not engage with the local culture from which students are drawn may mean that students fail to identify with the school to achieve a sense of belonging and commitment. Such students may seek affiliation from alternative sources of identity such as gangs and participate in violence and other disruptive behaviour (Brunson & Miller, 2009). Depending on the context, a potentially ‘balanced’ boundary between a school and the local community is one where schools acknowledge the cultural values of local families and communities and, at the same time, aim to protect students from DRV and GBV.

Where students fail to engage with a school's instructional and regulatory orders, it may be, for the students, a ‘rationally chosen’ course of action. Research has shown that in schools where students feel marginalised and unsafe, they cultivate ‘tough’ identities with aggressive and violent behaviour in order to develop relationships and bond with ‘tough’ peers (Brunson & Miller, 2009; Jamal et al., 2013; Mateu–Gelabert & Lune, 2007). Jamal et al. (2013) have argued that Markham and Aveyard's theory of human function and school organisation can be refined to acknowledge the importance of student agency and their social structures and networks. This could mean that students promote a ‘parallel informal’ student instructional and regulatory order in schools that, as argued by Jamal et al. (2013), in extreme cases functions in opposition to the school's orders and supports the continuation of anti‐social, violent attitudes and behaviours. To some extent, Markham's (2015) work on ‘valued school identities’ and how they can differ from ‘valued identities in school settings’ (e.g., peer groups) accommodates this thinking on the broader social context in which young people develop their behaviours. As Jamal et al. (2013, p. 8) observe, students are engaging in behaviour that is ‘often regarded as antisocial but which is thoroughly social in its origins, rather than stemming from an absence of student's practical reasoning, affiliation and autonomy as Markham & Aveyard suggest’. Thus, violent and aggressive behaviours may be about students enacting and displaying a sense of agency, even if that agency is ‘bounded’ (Evans, 2002) by broader structural influences.

The strengthening of relationships between teachers and students, particularly in terms of the quality of relationships, is widely regarded as important to health‐promoting schools. Research has shown that young people value good relationships with their teachers and appreciate good interpersonal behaviours such as respect and rapport (Gordon & Turner, 2001). However, there is evidence that students can perceive teachers as disconnected from the reality of their lives (Jamal et al., 2013), and in terms of knowledge of sexual harassment and online sexual abuse, children described their teachers as not ‘knowing the reality’ of their lives or being ‘out‐of‐date’ (Ofsted, 2021). The resulting lack of support for students is a clear example of a harmful boundary between teachers and students. Some of the interventions aimed to increase teacher awareness of DRV and GBV and enable greater responsiveness to students, thus attempting to address this aspect of the teacher‐student relationship.

### Limitations of the review

Although this systematic review had a comprehensive search strategy for DRV and GBV school‐based interventions, this synthesis only focuses on studies with a RCT design. We focused on theories of change presented in outcome evaluations and using a meta‐ethnographic approach allowed us to draw ‘synergetic ideas’ across the interventions and engage with authors' descriptions of theories of change. The evaluations on which we based this synthesis was not without its limitations. Authors differed in the level of detail they offered on the theories influencing the interventions; in some reports the detail could be sparse and was often implicit, while in others, the theory of change was very clear. The intervention theories of change were programme‐specific and informed by psychological theories of behaviour change such as the theory of planned behaviour (Ajzen, 1991) and social learning theory (Bandura, 1986). The focus was the individual and deficits in individual knowledge, skills and attitudes, ignoring other structural influences. By using methods of qualitative synthesis and the theory of human functioning and school organisation, we developed a deeper understanding of the mechanisms of the interventions and how they were intended to work. The strength of Markham and Aveyard's (2003) theory is that it is not ‘neatly packaged and ready to use’ (Moore & Evans, 2017, pp. 133–134), but with its concomitant ‘vagueness’ is the risk that it might offer a spurious reciprocal translation. We recognise that there is also the risk of attributing more weight to themes that align with the theory.

## CONCLUSION

This synthesis of theories of change offers insights into the mechanisms through which school‐based interventions for DRV and GBV are intended to work. Most interventions in this review were informed by ‘a limited range of psychological theories of behaviour change’ (Moore & Evans, 2017, p. 133), focusing on the individual and ‘psychological processes’ rather than the ‘deeper influences’ on student behaviour. Drawing on the sociological theory of human functioning and school organisation enabled us to develop an overarching theory of change student behaviour for both DRV and GBV interventions and explain the processes that underpin the interventions. While recognising the value of the theory of human functioning and school organisation in this synthesis, we argue that this theory synthesis on DRV and GBV contributes to the further development of the theory in the context of DRV and GBV.

## FUNDING INFORMATION

This research was supported by grant NIHR 130144 from the National Institute for Health Research (NIHR) Public Health Research Programme. In addition, Vashti Berry and G.J. Melendez‐Torres are part‐supported by the NIHR Applied Research Collaboration South West Peninsula (NIHR PenARC) and Chris Bonell is part‐funded by an NIHR senior investigator award. The findings, opinions and recommendations expressed in this paper are those of the authors and not necessarily of the university or the NIHR.

## CONFLICT OF INTEREST

Chris Bonell was the principal investigator, and Honor Young and G.J. Melendez‐Torres co‐investigators, of one of the trials included in this meta‐analysis.

## ETHICAL APPROVAL

Not applicable.

## PATIENT CONSENT

Not applicable.

## PERMISSION TO REPRODUCE MATERIAL FROM OTHER SOURCES

Not applicable.

## Supporting information


Appendix S1

Appendix S2

Appendix S3

Appendix S4

## Data Availability

The Supplementary Material files have full strategies for the original and supplementary searches, a summary of intervention theories of change, an example coding template and an example of the theory of change models, all referenced in the paper.
