# Supplementary material for: School‐based interventions for preventing dating and relationship violence and gender‐based violence: A systematic review and synthesis of theories of change
Source: Rev Educ. 2022 Dec 15;10(3):e3382. doi: 10.1002/rev3.3382 (PMC10116865; doi:10.1002/rev3.3382)
Supplement: Supplementary file 1 — Appendix S1 [file REV3-10-0-s002.docx]

**Supplementary Material 1**

**Full search terms and strategies: 2020 search**

**Ovid MEDLINE(R) ALL <1946 to June 19, 2020>**

**Search completed: 22/06/2020**

1 exp Intimate Partner Violence/ (9469)

2 Gender-Based Violence/ (188)

3 Stalking/ (203)

4 Rape/ (6264)

5 Sex Offenses/ (8867)

6 Battered Women/ (2620)

7 Spouse abuse/ (7352)

8 Coercion/ (4594)

9 Domestic violence/ (6375)

10 Homophobia/ (518)

11 (stalking or stalker*).ti,ab. (792)

12 rape*.ti,ab. (11749)

13 "intimate partner violence".ti,ab. (7375)

14 IPV.ti,ab. (6263)

15 (gender* adj3 violen*).ti,ab. (1623)

16 GBV.ti,ab. (1120)

17 SRGBV.ti,ab. (1)

18 (domestic adj3 (abuse* or abusive or aggressi* or assault* or attack* or bully* or coerc* or cyberbully* or femicid* or harass* or homicid* or injur* or manipulate* or murder* or rape* or threaten* or violen* or victimi?ation or revictimi?ation or re-victimi?ation)).ti,ab. (6880)

19 "violence against women".ti,ab. (2369)

20 ((date or dating) adj3 (abuse* or abusive or aggressi* or assault* or attack* or bully* or coerc* or cyberbully* or femicid* or harass* or homicid* or injur* or manipulate* or murder* or rape* or threaten* or violen* or victimi?ation or revictimi?ation or re-victimi?ation)).ti,ab. (2532)

21 ((relationship* or partner* or acquaintance* or non-stranger* or nonstranger*) adj3 (abuse* or abusive or aggressi* or assault* or attack* or bully* or coerc* or cyberbully* or femicid* or harass* or homicid* or injur* or manipulate* or murder* or rape* or threaten* or violen* or victimi?ation or revictimi?ation or re-victimi?ation)).ti,ab. (17167)

22 ((boyfriend* or boy-friend* or girlfriend* or girl-friend*) adj3 (abuse* or abusive or aggressi* or assault* or attack* or bully* or coerc* or cyberbully* or femicid* or harass* or homicid* or injur* or manipulate* or murder* or rape* or threaten* or violen* or victimi?ation or revictimi?ation or re-victimi?ation)).ti,ab. (47)

23 (interpersonal adj3 (abuse* or abusive or aggressi* or assault* or attack* or bully* or coerc* or cyberbully* or femicid* or harass* or homicid* or injur* or manipulate* or murder* or rape* or threaten* or violen* or victimi?ation or revictimi?ation or re-victimi?ation)).ti,ab. (2416)

24 (sexual* adj3 (abusive or aggressi* or assault* or attack* or bully* or coerc* or cyberbully* or femicid* or harass* or homicid* or injur* or manipulate* or murder* or rape* or threaten* or violen* or victimi?ation or revictimi?ation or re-victimi?ation)).ti,ab. (15390)

25 ((coerc* or forced or unwanted or nonconsensual or non-consensual) adj2 sex*).ti,ab. (2147)

26 (grope or groped or groping).ti,ab. (144)

27 (sext or sexts or sexting).ti,ab. (206)

28 (homophobi* or transphobi* or biphobi* or homonegativ*).ti,ab. (1662)

29 ((LGB or LGBT* or homosexual* or lesbian* or gay or bisexual* or queer* or transgender* or transsexual*) adj3 (abuse* or abusive or aggressi* or assault* or attack* or bully* or coerc* or cyberbully* or femicid* or harass* or homicid* or injur* or manipulate* or murder* or rape* or threaten* or violen* or victimi?ation or revictimi?ation or re-victimi?ation)).ti,ab. (697)

30 "long live love".ti,ab. (4)

31 (greendot or "green dot").ti,ab. (28)

32 "project respect".ti,ab. (27)

33 ("Media Aware" or mediaaware).ti,ab. (5)

34 TakeCARE.ti,ab. (14)

35 "Fourth R".ti,ab. (24)

36 "Safe Dates".ti,ab. (22)

37 "Shifting boundaries".ti,ab. (49)

38 "Teen choices".ti,ab. (4)

39 "good schools toolkit".ti,ab. (2)

40 "mentors in violence prevention".ti,ab. (5)

41 "Expect Respect".ti,ab. (8)

42 "Second Step".ti,ab. (10890)

43 SS-SSTP.ti,ab. (1)

44 "It's your game".ti,ab. (12)

45 DaVIPoP.ti,ab. (0)

46 (Benzies adj2 Batchies).ti,ab. (1)

47 or/1-46 (80507)

48 Schools/ (37880)

49 exp School Health Services/ (22957)

50 Students/ (58229)

51 Curriculum/ (74944)

52 school*.ti,ab,jw. (290890)

53 (pupil or pupils).ti,ab. (21829)

54 (classroom* or class-room*).ti,ab. (17106)

55 or/48-54 (420474)

56 47 and 55 (5600)

**Ovid Embase (1974 to 2020 June 19)**

**Searched completed: 22/06/2020**

1 exp partner violence/ [NT marital rape] (12187)

2 dating violence/ (501)

3 gender based violence/ (727)

4 exp sexual assault/ [NT drug-facilitated sexual assault, rape, acquaintance rape, attempted rape, sexual abuse, sexual harassment, sexual bullying] (34784)

5 exp stalking/ [NT cyberstalking] (635)

6 sexual violence/ (2425)

7 sexual coercion/ (366)

8 sexual exploitation/ (442)

9 sexual crime/ (11136)

10 battered woman/ (3216)

11 domestic violence/ (8764)

12 sexting/ (201)

13 homophobia/ (1082)

14 (stalking or stalker*).ti,ab. (917)

15 rape*.ti,ab. (12720)

16 IPV.ti,ab. (6697)

17 (gender* adj3 violen*).ti,ab. (1823)

18 GBV.ti,ab. (1308)

19 SRGBV.ti,ab. (0)

20 (domestic adj3 (abuse* or abusive or aggressi* or assault* or attack* or bully* or coerc* or cyberbully* or femicid* or harass* or homicid* or injur* or manipulate* or murder* or rape* or threaten* or violen* or victimi?ation or revictimi?ation or re-victimi?ation)).ti,ab. (8187)

21 "violence against women".ti,ab. (2457)

22 ((date or dating) adj3 (abuse* or abusive or aggressi* or assault* or attack* or bully* or coerc* or cyberbully* or femicid* or harass* or homicid* or injur* or manipulate* or murder* or rape* or threaten* or violen* or victimi?ation or revictimi?ation or re-victimi?ation)).ti,ab. (3100)

23 ((relationship* or partner* or acquaintance* or non-stranger* or nonstranger*) adj3 (abuse* or abusive or aggressi* or assault* or attack* or bully* or coerc* or cyberbully* or femicid* or harass* or homicid* or injur* or manipulate* or murder* or rape* or threaten* or violen* or victimi?ation or revictimi?ation or re-victimi?ation)).ti,ab. (19267)

24 ((boyfriend* or boy-friend* or girlfriend* or girl-friend*) adj3 (abuse* or abusive or aggressi* or assault* or attack* or bully* or coerc* or cyberbully* or femicid* or harass* or homicid* or injur* or manipulate* or murder* or rape* or threaten* or violen* or victimi?ation or revictimi?ation or re-victimi?ation)).ti,ab. (52)

25 (interpersonal adj3 (abuse* or abusive or aggressi* or assault* or attack* or bully* or coerc* or cyberbully* or femicid* or harass* or homicid* or injur* or manipulate* or murder* or rape* or threaten* or violen* or victimi?ation or revictimi?ation or re-victimi?ation)).ti,ab. (2828)

26 (sexual* adj3 (abusive or aggressi* or assault* or attack* or bully* or coerc* or cyberbully* or femicid* or harass* or homicid* or injur* or manipulate* or murder* or rape* or threaten* or violen* or victimi?ation or revictimi?ation or re-victimi?ation)).ti,ab. (18545)

27 ((coerc* or forced or unwanted or nonconsensual or non-consensual) adj2 sex*).ti,ab. (2604)

28 (grope or groped or groping).ti,ab. (236)

29 (sext or sexts or sexting).ti,ab. (286)

30 (homophobi* or transphobi* or biphobi* or homonegativ*).ti,ab. (1816)

31 ((LGB or LGBT* or homosexual* or lesbian* or gay or bisexual* or queer* or transgender* or transsexual) adj3 (abuse* or abusive or aggressi* or assault* or attack* or bully* or coerc* or cyberbully* or femicid* or harass* or homicid* or injur* or manipulate* or murder* or rape* or threaten* or violen* or victimi?ation or revictimi?ation or re-victimi?ation)).ti,ab. (770)

32 "long live love".ti,ab. (5)

33 (greendot or "green dot").ti,ab. (47)

34 "project respect".ti,ab. (30)

35 ("Media Aware" or mediaaware).ti,ab. (4)

36 TakeCARE.ti,ab. (15)

37 "Fourth R".ti,ab. (36)

38 "Safe Dates".ti,ab. (22)

39 "Shifting boundaries".ti,ab. (46)

40 "Teen choices".ti,ab. (2)

41 "good schools toolkit".ti,ab. (2)

42 "mentors in violence prevention".ti,ab. (5)

43 "Expect Respect".ti,ab. (10)

44 "Second Step".ti,ab. (14194)

45 SS-SSTP.ti,ab. (1)

46 "It's your game".ti,ab. (10)

47 DaVIPoP.ti,ab. (0)

48 (Benzies adj2 Batchies).ti,ab. (1)

49 or/1-48 (109958)

50 school/ or high school/ or kindergarten/ or middle school/ or nursery school/ or primary school/ (92210)

51 school health service/ (13288)

52 school health nursing/ (5288)

53 school*.ti,ab,jx. (354704)

54 (pupil or pupils).ti,ab. (27879)

55 (classroom* or class-room*).ti,ab. (20145)

56 or/50-55 (402120)

57 49 and 56 (5944)

**Ovid APA PsycINFO 1806 to June Week 3 2020**

**Search completed: 22/06/2020**

1. intimate partner violence/

2. stalking/

3. exp rape/

4. sex offenses/

5. sexual harassment/

6. battered females/

7. coercion/

8. domestic violence/

9. sexting/

10. (stalking or stalker*).ti,ab.

11. rape*.ti,ab.

12. "intimate partner violence".ti,ab.

13. IPV.ti,ab.

14. (gender* adj3 violen*).ti,ab.

15. GBV.ti,ab.

16. SRGBV.ti,ab.

17. (domestic adj3 (abuse* or abusive or aggressi* or assault* or attack* or bully* or coerc* or cyberbully* or femicid* or harass* or homicid* or injur* or manipulat* or murder* or rape* or threaten* or violen* or victimi?ation or revictimi?ation or re-victimi?ation)).ti,ab.

18. "violence against women".ti,ab.

19. ((date or dating) adj3 (abuse* or abusive or aggressi* or assault* or attack* or bully* or coerc* or cyberbully* or femicid* or harass* or homicid* or injur* or manipulat* or murder* or rape* or threaten* or violen* or victimi?ation or revictimi?ation or re-victimi?ation)).ti,ab.

20. ((relationship* or partner* or acquaintance* or non-stranger* or nonstranger*) adj3 (abuse* or abusive or aggressi* or assault* or attack* or bully* or coerc* or cyberbully* or femicid* or harass* or homicid* or injur* or manipulat* or murder* or rape* or threaten* or violen* or victimi?ation or revictimi?ation or re-victimi?ation)).ti,ab.

21. ((boyfriend* or boy-friend* or girlfriend* or girl-friend*) adj3 (abuse* or abusive or aggressi* or assault* or attack* or bully* or coerc* or cyberbully* or femicid* or harass* or homicid* or injur* or manipulat* or murder* or rape* or threaten* or violen* or victimi?ation or revictimi?ation or re-victimi?ation)).ti,ab.

22. (interpersonal adj3 (abuse* or abusive or aggressi* or assault* or attack* or bully* or coerc* or cyberbully* or femicid* or harass* or homicid* or injur* or manipulat* or murder* or rape* or threaten* or violen* or victimi?ation or revictimi?ation or re-victimi?ation)).ti,ab.

23. (sexual* adj2 (abuse* or abusive or aggressi* or assault* or attack* or bully* or coerc* or cyberbully* or femicid* or harass* or homicid* or injur* or manipulat* or murder* or rape* or threaten* or violen* or victimi?ation or revictimi?ation or re-victimi?ation)).ti,ab.

24. ((coerc* or forced or unwanted or nonconsensual or non-consensual) adj2 sex*).ti,ab.

25. (grope or groped or groping).ti,ab.

26. (sext or sexts or sexting).ti,ab.

27. (homophobi* or transphobi* or biphobi* or homonegativ*).ti,ab.

28. ((LGB or LGBT* or homosexual* or lesbian* or gay or bisexual* or queer* or transgender* or transsexual*) adj3 (abuse* or abusive or aggressi* or assault* or attack* or bully* or coerc* or cyberbully* or femicid* or harass* or homicid* or injur* or manipulate* or murder* or rape* or threaten* or violen* or victimi?ation or revictimi?ation or re-victimi?ation)).ti,ab.

29. "long live love".ti,ab.

30. (greendot or "green dot").ti,ab.

31. "project respect".ti,ab.

32. ("Media Aware" or mediaaware).ti,ab.

33. TakeCARE.ti,ab.

34. "Fourth R".ti,ab.

35. "Safe Dates".ti,ab.

36. "Shifting boundaries".ti,ab.

37. "Teen choices".ti,ab.

38. "good schools toolkit".ti,ab.

39. "mentors in violence prevention".ti,ab.

40. "Expect Respect".ti,ab.

41. "Second Step".ti,ab.

42. SS-SSTP.ti,ab.

43. "It's your game".ti,ab.

44. DaVIPoP.ti,ab.

45. (Benzies adj2 Batchies).ti,ab.

46. or/1-45

47. exp schools/

48. school based intervention/

49. students/ or high school graduates/ or high school students/ or junior high school students/ or kindergarten students/ or middle school students/ or preschool students/

50. exp curriculum/

51. school*.ti,ab,jn.

52. (pupil or pupils).ti,ab.

53. (classroom* or class-room*).ti,ab.

54. or/47-53

55. 46 and 54 (8716)

**Ovid Social Policy and Practice <1890s - 202004>**

**Search completed: 22/06/2020**

1 (stalking or stalker*).ti,ab. (234)

2 rape*.ti,ab. (962)

3 "intimate partner violence".ti,ab. (2013)

4 IPV.ti,ab. (1232)

5 (gender* adj3 violen*).ti,ab. (429)

6 GBV.ti,ab. (32)

7 SRGBV.ti,ab. (0)

8 (domestic adj3 (abuse* or abusive or aggress* or assault* or attack* or bully* or coerc* or cyberbully* or femicid* or harass* or homicid* or injur* or manipulat* or murder* or rape* or threaten* or violen* or victimi?ation or revictimi?ation or re-victimi?ation)).ti,ab. (7229)

9 "violence against women".ti,ab. (857)

10 ((date or dating) adj3 (abuse* or abusive or aggress* or assault* or attack* or bully* or coerc* or cyberbully* or femicid* or harass* or homicid* or injur* or manipulat* or murder* or rape* or threaten* or violen* or victimi?ation or revictimi?ation or re-victimi?ation)).ti,ab. (345)

11 ((relationship* or partner* or acquaintance* or non-stranger* or nonstranger*) adj3 (abuse* or abusive or aggress* or assault* or attack* or bully* or coerc* or cyberbully* or femicid* or harass* or homicid* or injur* or manipulat* or murder* or rape* or threaten* or violen* or victimi?ation or revictimi?ation or re-victimi?ation)).ti,ab. (4871)

12 ((boyfriend* or boy-friend* or girlfriend* or girl-friend*) adj3 (abuse* or abusive or aggress* or assault* or attack* or bully* or coerc* or cyberbully* or femicid* or harass* or homicid* or injur* or manipulat* or murder* or rape* or threaten* or violen* or victimi?ation or revictimi?ation or re-victimi?ation)).ti,ab. (23)

13 (interpersonal adj3 (abuse* or abusive or aggress* or assault* or attack* or bully* or coerc* or cyberbully* or femicid* or harass* or homicid* or injur* or manipulat* or murder* or rape* or threaten* or violen* or victimi?ation or revictimi?ation or re-victimi?ation)).ti,ab. (531)

14 (sexual* adj2 (abuse* or abusive or aggress* or assault* or attack* or bully* or coerc* or cyberbully* or femicid* or harass* or homicid* or injur* or manipulat* or murder* or rape* or threaten* or violen* or victimi?ation or revictimi?ation or re-victimi?ation)).ti,ab. (13400)

15 ((coerc* or forced or unwanted or nonconsensual or non-consensual) adj2 sex*).ti,ab. (357)

16 (grope or groped or groping).ti,ab. (2)

17 (sext or sexts or sexting).ti,ab. (143)

18 (homophobi* or transphobi* or biphobi* or homonegativ*).ti,ab. (698)

19 ((LGB or LGBT* or homosexual* or lesbian* or gay or bisexual* or queer* or transgender* or transsexual) adj3 (abuse* or abusive or aggress* or assault* or attack* or bully* or coerc* or cyberbully* or femicid* or harass* or homicid* or injur* or manipulat* or murder* or rape* or threaten* or violen* or victimi?ation or revictimi?ation or re-victimi?ation)).ti,ab. (279)

20 "long live love".ti,ab. (0)

21 (greendot or "green dot").ti,ab. (1)

22 "project respect".ti,ab. (0)

23 ("Media Aware" or mediaaware).ti,ab. (0)

24 TakeCARE.ti,ab. (0)

25 "Fourth R".ti,ab. (7)

26 "Safe Dates".ti,ab. (3)

27 "Shifting boundaries".ti,ab. (36)

28 "Teen choices".ti,ab. (0)

29 "good schools toolkit".ti,ab. (0)

30 "mentors in violence prevention".ti,ab. (1)

31 "Expect Respect".ti,ab. (6)

32 "Second Step".ti,ab. (34)

33 SS-SSTP.ti,ab. (0)

34 "It's your game".ti,ab. (0)

35 DaVIPoP.ti,ab. (0)

36 (Benzies adj2 Batchies).ti,ab. (0)

37 or/1-36 (25164)

38 school*.ti,ab,jx. (32423)

39 (pupil or pupils).ti,ab. (5107)

40 (classroom* or class-room*).ti,ab. (2299)

41 or/38-40 (34253)

42 37 and 41 (1857)

**EBSCO CINAHL Complete (1937-2020)**

**Search completed: 23/06/2020**

S55 S46 AND S54 3,823

S54 S47 OR S48 OR S49 OR S50 OR S51 OR S52 OR S53 190,926

S53 TI ( classroom* or class-room* ) OR AB ( classroom* or class-room* ) 14,057

S52 TI ( pupil or pupils ) OR AB ( pupil or pupils ) 4,775

S51 TI school* OR AB school* 153,362

S50 SO school* 19,849

S49 (MH "Students, High School") OR (MH "Students, Middle School") OR (MH "Students, Elementary") 20,105

S48 (MH "School Health Services+") 24,085

S47 (MH "Schools") OR (MH "Schools, Elementary") OR (MH "Schools, Middle") OR (MH "Schools, Nursery") OR (MH "Schools, Secondary") 26,668

S46 S1 OR S2 OR S3 OR S4 OR S5 OR S6 OR S7 OR S8 OR S9 OR S10 OR S11 OR S12 OR S13 OR S14 OR S15 OR S16 OR S17 OR S18 OR S19 OR S20 OR S21 OR S22 OR S23 OR S24 OR S25 OR S26 OR S27 OR S28 OR S29 OR S30 OR S31 OR S32 OR S33 OR S34 OR S35 OR S36 OR S37 OR S38 OR S39 OR S40 OR S41 OR S42 OR S43 OR S44 OR S45 48,807

S45 TI (Benzies N2 Batchies) OR AB (Benzies N2 Batchies) 1

S44 TI DaVIPoP OR AB DaVIPoP 0

S43 TI "It's your game" OR AB "It's your game" 13

S42 TI SS-SSTP OR AB SS-SSTP 4

S41 TI "Second Step" OR AB "Second Step" 1,005

S40 TI "Expect Respect" OR AB "Expect Respect" 15

S39 TI "mentors in violence prevention" OR AB "mentors in violence prevention" 6

S38 TI "good schools toolkit" OR AB "good schools toolkit" 2

S37 TI "Teen choices" OR AB "Teen choices" 6

S36 TI "Shifting boundaries" OR AB "Shifting boundaries" 38

S35 TI "Safe Dates" OR AB "Safe Dates" 24

S34 TI "Fourth R" OR AB "Fourth R" 12

S33 TI TakeCARE OR AB TakeCARE 6

S32 TI ( ("Media Aware" or mediaaware) ) OR AB ( ("Media Aware" or mediaaware) ) 5

S31 TI "project respect" OR AB "project respect" 14

S30 TI ( (greendot or "green dot") ) OR AB ( (greendot or "green dot") ) 17

S29 TI "long live love" OR AB "long live love" 5

S28 TI ( ((LGB or LGBT* or homosexual* or lesbian* or gay or bisexual* or queer* or transgender* or transsexual) N3 (abuse* or abusive or aggressi* or assault* or attack* or bully* or coerc* or cyberbully* or Femicid* or harass* or homicid* or injur* or manipulate* or murder* or rape* or threaten* or violen* or victimi?ation or revictimi?ation or re-victimi?ation)) ) OR AB ( ((LGB or LGBT* or homosexual* or lesbian* or gay or bisexual* or queer* or transgender* or transsexual) N3 (abuse* or abusive or aggressi* or assault* or attack* or bully* or coerc* or cyberbully* or Femicid* or harass* or homicid* or injur* or manipulate* or murder* or rape* or threaten* or violen* or victimi?ation or revictimi?ation or re-victimi?ation)) ) 583

S27 TI ( (homophobi* or transphobi* or biphobi* or homonegativ*) ) OR AB ( (homophobi* or transphobi* or biphobi* or homonegativ*) ) 1,343

S26 TI ( (sext or sexts or sexting) ) OR AB ( (sext or sexts or sexting) ) 276

S25 TI ( (grope or groped or groping) ) OR AB ( (grope or groped or groping) ) 50

S24 TI ( ((coerc* or forced or unwanted or nonconsensual or non-consensual) N2 sex*) ) OR AB ( ((coerc* or forced or unwanted or nonconsensual or non-consensual) N2 sex*) ) 1,732

S23 TI ( (sexual* N3 (abusive or aggressi* or assault* or attack* or bully* or coerc* or cyberbully* or femicid* or harass* or homicid* or injur* or manipulate* or murder* or rape* or threaten* or violen* or victimi?ation or revictimi?ation or re-victimi?ation)) ) OR AB ( (sexual* N3 (abusive or aggressi* or assault* or attack* or bully* or coerc* or cyberbully* or femicid* or harass* or homicid* or injur* or manipulate* or murder* or rape* or threaten* or violen* or victimi?ation or revictimi?ation or re-victimi?ation)) ) 11,328

S22 TI ( (interpersonal N3 (abuse* or abusive or aggressi* or assault* or attack* or bully* or coerc* or cyberbully* or femicid* or harass* or homicid* or injur* or manipulate* or murder* or rape* or threaten* or violen* or victimi?ation or revictimi?ation or re-victimi?ation)) ) OR AB ( (interpersonal N3 (abuse* or abusive or aggressi* or assault* or attack* or bully* or coerc* or cyberbully* or femicid* or harass* or homicid* or injur* or manipulate* or murder* or rape* or threaten* or violen* or victimi?ation or revictimi?ation or re-victimi?ation)) ) 1,677

S21 TI ( ((boyfriend* or boy-friend* or girlfriend* or girl-friend*) N3 (abuse* or abusive or aggressi* or assault* or attack* or bully* or coerc* or cyberbully* or femicid* or harass* or homicid* or injur* or manipulate* or murder* or rape* or threaten* or violen* or victimi?ation or revictimi?ation or re-victimi?ation)) ) OR AB ( ((boyfriend* or boy-friend* or girlfriend* or girl-friend*) N3 (abuse* or abusive or aggressi* or assault* or attack* or bully* or coerc* or cyberbully* or femicid* or harass* or homicid* or injur* or manipulate* or murder* or rape* or threaten* or violen* or victimi?ation or revictimi?ation or re-victimi?ation)) ) 31

S20 TI ( ((relationship* or partner* or acquaintance* or non-stranger* or nonstranger*) N3 (abuse* or abusive or aggressi* or assault* or attack* or bully* or coerc* or cyberbully* or Femicid* or harass* or homicid* or injur* or manipulate* or murder* or rape* or threaten* or violen* or victimi?ation or revictimi?ation or re-victimi?ation)) ) OR AB ( ((relationship* or partner* or acquaintance* or non-stranger* or nonstranger*) N3 (abuse* or abusive or aggressi* or assault* or attack* or bully* or coerc* or cyberbully* or Femicid* or harass* or homicid* or injur* or manipulate* or murder* or rape* or threaten* or violen* or victimi?ation or revictimi?ation or re-victimi?ation)) ) 15,614

S19 TI ( ((date or dating) N3 (abuse* or abusive or aggressi* or assault* or attack* or bully* or coerc* or cyberbully* or femicid* or harass* or homicid* or injur* or manipulate* or murder* or rape* or threaten* or violen* or victimi?ation or revictimi?ation or re-victimi?ation)) ) OR AB ( ((date or dating) N3 (abuse* or abusive or aggressi* or assault* or attack* or bully* or coerc* or cyberbully* or femicid* or harass* or homicid* or injur* or manipulate* or murder* or rape* or threaten* or violen* or victimi?ation or revictimi?ation or re-victimi?ation)) ) 2,124

S18 TI "violence against women" OR AB "violence against women" 2,114

S17 TI ( (domestic N3 (abuse* or abusive or aggressi* or assault* or attack* or bully* or coerc* or cyberbully* or femicid* or harass* or homicid* or injur* or manipulate* or murder* or rape* or threaten* or violen* or victimi?ation or revictimi?ation or re-victimi?ation)) ) OR AB ( (domestic N3 (abuse* or abusive or aggressi* or assault* or attack* or bully* or coerc* or cyberbully* or femicid* or harass* or homicid* or injur* or manipulate* or murder* or rape* or threaten* or violen* or victimi?ation or revictimi?ation or re-victimi?ation)) ) 7,162

S16 TI SRGBV OR AB SRGBV 0

S15 TI GBV OR AB GBV 184

S14 TI (gender* N3 violen*) OR AB (gender* N3 violen*) 1,481

S13 TI IPV OR AB IPV 4,415

S12 TI rape* OR AB rape* 3,035

S11 TI ( (stalking or stalker*) ) OR AB ( (stalking or stalker*) ) 515

S10 (MH "Homophobia") 1,342

S9 (MH "Dating Violence") 811

S8 (MH "Domestic Violence") 9,594

S7 (MH "Coercion") 2,295

S6 (MH "Battered Men") 124

S5 (MH "Battered Women") 4,228

S4 (MH "Rape") 4,084

S3 (MH "Stalking") 437

S2 (MH "Gender-Based Violence") 203

S1 (MH "Intimate Partner Violence") 11,419

**EBSCO Child and Adolescent Development (1855-)**

**Search completed: 23/06/2020**

S53 S45 AND S52 2,121

S52 S46 OR S47 OR S48 OR S49 OR S50 OR S51

S51 TI ( classroom* or class-room* ) OR AB ( classroom* or class-room* )

S50 TI ( pupil or pupils ) OR AB ( pupil or pupils )

S49 TI school* OR AB school* OR SO school*

S48 ((((ZU "elementary schools")) or ((ZU "nursery schools") or (ZU "nursery schools (great britain)"))) or ((ZU "middle schools"))) or ((ZU "high schools"))

S47 (ZU "school health services")

S46 (ZU "schools")

S45 S1 OR S2 OR S3 OR S4 OR S5 OR S6 OR S7 OR S8 OR S9 OR S10 OR S11 OR S12 OR S13 OR S14 OR S15 OR S16 OR S17 OR S18 OR S19 OR S20 OR S21 OR S22 OR S23 OR S24 OR S25 OR S26 OR S27 OR S28 OR S29 OR S30 OR S31 OR S32 OR S33 OR S34 OR S35 OR S36 OR S37 OR S38 OR S39 OR S40 OR S41 OR S42 OR S43 OR S44 11,832

S44 TI (Benzies N2 Batchies) OR AB (Benzies N2 Batchies)

S43 TI DaVIPoP OR AB DaVIPoP

S42 TI "It's your game" OR AB "It's your game"

S41 TI SS-SSTP OR AB SS-SSTP

S40 TI "Second Step" OR AB "Second Step"

S39 TI "Expect Respect" OR AB "Expect Respect"

S38 TI "mentors in violence prevention" OR AB "mentors in violence prevention"

S37 TI "good schools toolkit" OR AB "good schools toolkit"

S36 TI "good schools toolkit" OR AB "good schools toolkit"

S35 TI "Teen choices" OR AB "Teen choices"

S34 TI "Shifting boundaries" OR AB "Shifting boundaries"

S33 TI "Safe Dates" OR AB "Safe Dates"

S32 TI "Fourth R" OR AB "Fourth R"

S31 TI TakeCARE OR AB TakeCARE

S30 TI ( ("Media Aware" or mediaaware) ) OR AB ( ("Media Aware" or mediaaware) )

S29 TI "project respect" OR AB "project respect"

S28 TI "project respect" OR AB "project respect"

S27 TI ( (greendot or "green dot") ) OR AB ( (greendot or "green dot") )

S26 TI "long live love" OR AB "long live love"

S25 TI "long live love" OR AB "long live love"

S24 TI ( ((LGB or LGBT* or homosexual* or lesbian* or gay or bisexual* or queer* or transgender* or transsexual) N3 (abuse* or abusive or aggressi* or assault* or attack* or bully* or coerc* or cyberbully* or Femicid* or harass* or homicid* or injur* or manipulate* or murder* or rape* or threaten* or violen* or victimi?ation or revictimi?ation or re-victimi?ation)) ) OR AB ( ((LGB or LGBT* or homosexual* or lesbian* or gay or bisexual* or queer* or transgender* or transsexual) N3 (abuse* or abusive or aggressi* or assault* or attack* or bully* or coerc* or cyberbully* or Femicid* or harass* or homicid* or injur* or manipulate* or murder* or rape* or threaten* or violen* or victimi?ation or revictimi?ation or re-victimi?ation)) )

S23 TI ( (homophobi* or transphobi* or biphobi* or homonegativ*) ) OR AB ( (homophobi* or transphobi* or biphobi* or homonegativ*) )

S22 TI ( (sext or sexts or sexting) ) OR AB ( (sext or sexts or sexting) )

S21 TI ( (grope or groped or groping) ) OR AB ( (grope or groped or groping) )

S20 TI ( ((coerc* or forced or unwanted or nonconsensual or non-consensual) N2 sex*) ) OR AB ( ((coerc* or forced or unwanted or nonconsensual or non-consensual) N2 sex*) )

S19 TI ( (sexual* N3 (abusive or aggressi* or assault* or attack* or bully* or coerc* or cyberbully* or femicid* or harass* or homicid* or injur* or manipulate* or murder* or rape* or threaten* or violen* or victimi?ation or revictimi?ation or re-victimi?ation)) ) OR AB ( (sexual* N3 (abusive or aggressi* or assault* or attack* or bully* or coerc* or cyberbully* or femicid* or harass* or homicid* or injur* or manipulate* or murder* or rape* or threaten* or violen* or victimi?ation or revictimi?ation or re-victimi?ation)) )

S18 TI ( (interpersonal N3 (abuse* or abusive or aggressi* or assault* or attack* or bully* or coerc* or cyberbully* or femicid* or harass* or homicid* or injur* or manipulate* or murder* or rape* or threaten* or violen* or victimi?ation or revictimi?ation or re-victimi?ation)) ) OR AB ( (interpersonal N3 (abuse* or abusive or aggressi* or assault* or attack* or bully* or coerc* or cyberbully* or femicid* or harass* or homicid* or injur* or manipulate* or murder* or rape* or threaten* or violen* or victimi?ation or revictimi?ation or re-victimi?ation)) )

S17 TI ( ((boyfriend* or boy-friend* or girlfriend* or girl-friend*) N3 (abuse* or abusive or aggressi* or assault* or attack* or bully* or coerc* or cyberbully* or femicid* or harass* or homicid* or injur* or manipulate* or murder* or rape* or threaten* or violen* or victimi?ation or revictimi?ation or re-victimi?ation)) ) OR AB ( ((boyfriend* or boy-friend* or girlfriend* or girl-friend*) N3 (abuse* or abusive or aggressi* or assault* or attack* or bully* or coerc* or cyberbully* or femicid* or harass* or homicid* or injur* or manipulate* or murder* or rape* or threaten* or violen* or victimi?ation or revictimi?ation or re-victimi?ation)) )

S16 TI ( ((relationship* or partner* or acquaintance* or non-stranger* or nonstranger*) N3 (abuse* or abusive or aggressi* or assault* or attack* or bully* or coerc* or cyberbully* or Femicid* or harass* or homicid* or injur* or manipulate* or murder* or rape* or threaten* or violen* or victimi?ation or revictimi?ation or re-victimi?ation)) ) OR AB ( ((relationship* or partner* or acquaintance* or non-stranger* or nonstranger*) N3 (abuse* or abusive or aggressi* or assault* or attack* or bully* or coerc* or cyberbully* or Femicid* or harass* or homicid* or injur* or manipulate* or murder* or rape* or threaten* or violen* or victimi?ation or revictimi?ation or re-victimi?ation)) )

S15 TI ( ((date or dating) N3 (abuse* or abusive or aggressi* or assault* or attack* or bully* or coerc* or cyberbully* or femicid* or harass* or homicid* or injur* or manipulate* or murder* or rape* or threaten* or violen* or victimi?ation or revictimi?ation or re-victimi?ation)) ) OR AB ( ((date or dating) N3 (abuse* or abusive or aggressi* or assault* or attack* or bully* or coerc* or cyberbully* or femicid* or harass* or homicid* or injur* or manipulate* or murder* or rape* or threaten* or violen* or victimi?ation or revictimi?ation or re-victimi?ation)) )

S14 TI "violence against women" OR AB "violence against women"

S13 TI ( (domestic N3 (abuse* or abusive or aggressi* or assault* or attack* or bully* or coerc* or cyberbully* or femicid* or harass* or homicid* or injur* or manipulate* or murder* or rape* or threaten* or violen* or victimi?ation or revictimi?ation or re-victimi?ation)) ) OR AB ( (domestic N3 (abuse* or abusive or aggressi* or assault* or attack* or bully* or coerc* or cyberbully* or femicid* or harass* or homicid* or injur* or manipulate* or murder* or rape* or threaten* or violen* or victimi?ation or revictimi?ation or re-victimi?ation)) )

S12 TI SRGBV OR AB SRGBV

S11 TI GBV OR AB GBV

S10 TI (gender* N3 violen*) OR AB (gender* N3 violen*)

S9 TI IPV OR AB IPV

S8 TI rape* OR AB rape*

S7 TI ( (stalking or stalker*) ) OR AB ( (stalking or stalker*) )

S6 ((ZU "dating violence -- prevention")) or ((ZU "dating violence"))

S5 (ZU "homophobia")

S4 (ZU "domestic violence")

S3 (ZU "rape") or (ZU "rape -- prevention")

S2 (ZU "stalking") or (ZU "stalking -- prevention")

S1 (ZU "intimate partner violence") or (ZU "intimate partner violence -- prevention") or (ZU "abused women") or (ZU "sex crimes") or (ZU "sexual harassment") or (ZU "prevention of sexual assault")

**EBSCO British Education Index (1929-)**

**Search completed: 23/06/2020**

S48 S40 AND S47 315

S47 S41 OR S42 OR S43 OR S44 OR S45 OR S46 94,739

S46 TI ( classroom* or class-room* ) OR AB ( classroom* or class-room* ) 13,345

S45 TI ( pupil or pupils ) OR AB ( pupil or pupils ) 6,573

S44 TI school* OR AB school* 54,792

S43 DE "BOARDING school students" OR DE "MIDDLE school students" OR DE "PREPARATORY school students" OR DE "SCHOOL children" OR DE "SECONDARY school students" OR DE "SIXTH form students" 14,480

S42 DE "SCHOOL health services" OR DE "COORDINATED school health programs" 144

S41 DE "SCHOOLS" OR DE "BOARDING schools" OR DE "DISADVANTAGED schools" OR DE "ELEMENTARY schools" OR DE "PRESCHOOLS" OR DE "PRIMARY schools" OR DE "PRIVATE schools" OR DE "PUBLIC schools" OR DE "RURAL schools" OR DE "SINGLE sex schools" OR DE "TRADITIONAL schools" OR DE "URBAN schools" OR DE "HIGH schools" OR DE "MIDDLE schools" OR DE "Secondary Education" 47,092

S40 S1 OR S2 OR S3 OR S4 OR S5 OR S6 OR S7 OR S8 OR S9 OR S10 OR S11 OR S12 OR S13 OR S14 OR S15 OR S16 OR S17 OR S18 OR S19 OR S20 OR S21 OR S22 OR S23 OR S24 OR S25 OR S26 OR S27 OR S28 OR S29 OR S30 OR S31 OR S32 OR S33 OR S34 OR S35 OR S36 OR S37 OR S38 OR S39 713

S39 TI (Benzies N2 Batchies) OR AB (Benzies N2 Batchies) 0

S38 TI DaVIPoP OR AB DaVIPoP 0

S37 TI "It's your game" OR AB "It's your game" 0

S36 TI SS-SSTP OR AB SS-SSTP 0

S35 TI "Second Step" OR AB "Second Step" 35

S34 TI "Expect Respect" OR AB "Expect Respect" 0

S33 TI "mentors in violence prevention" OR AB "mentors in violence prevention" 0

S32 TI "good schools toolkit" OR AB "good schools toolkit" 0

S31 TI "Teen choices" OR AB "Teen choices" 0

S30 TI "Shifting boundaries" OR AB "Shifting boundaries" 12

S29 TI "Safe Dates" OR AB "Safe Dates" 0

S28 TI "Fourth R" OR AB "Fourth R" 7

S27 TI TakeCARE OR AB TakeCARE 0

S26 TI ( ("Media Aware" or mediaaware) ) OR AB ( ("Media Aware" or mediaaware) ) 0

S25 TI "project respect" OR AB "project respect" 0

S24 TI ( (greendot or "green dot") ) OR AB ( (greendot or "green dot") ) 1

S23 TI "long live love" OR AB "long live love" 3

S22 TI ( ((LGB or LGBT* or homosexual* or lesbian* or gay or bisexual* or queer* or transgender* or transsexual) N3 (abuse* or abusive or aggressi* or assault* or attack* or bully* or coerc* or cyberbully* or femicid* or harass* or homicid* or injur* or manipulate* or murder* or rape* or threaten* or violen* or victimi?ation or revictimi?ation or re-victimi?ation)) ) OR AB ( ((LGB or LGBT* or homosexual* or lesbian* or gay or bisexual* or queer* or transgender* or transsexual) N3 (abuse* or abusive or aggressi* or assault* or attack* or bully* or coerc* or cyberbully* or femicid* or harass* or homicid* or injur* or manipulate* or murder* or rape* or threaten* or violen* or victimi?ation or revictimi?ation or re-victimi?ation)) ) 21

S21 TI ( (homophobi* or transphobi* or biphobi* or homonegativ*) ) OR AB ( (homophobi* or transphobi* or biphobi* or homonegativ*) ) 119

S20 TI ( (sext or sexts or sexting) ) OR AB ( (sext or sexts or sexting) ) 16

S19 TI ( (grope or groped or groping) ) OR AB ( (grope or groped or groping) ) 2

S18 TI ( ((coerc* or forced or unwanted or nonconsensual or non-consensual) N2 sex*) ) OR AB ( ((coerc* or forced or unwanted or nonconsensual or non-consensual) N2 sex*) ) 14

S17 TI ( (sexual* N3 (abusive or aggressi* or assault* or attack* or bully* or coerc* or cyberbully* or femicid* or harass* or homicid* or injur* or manipulate* or murder* or rape* or threaten* or violen* or victimi?ation or revictimi?ation or re-victimi?ation)) ) OR AB ( (sexual* N3 (abusive or aggressi* or assault* or attack* or bully* or coerc* or cyberbully* or femicid* or harass* or homicid* or injur* or manipulate* or murder* or rape* or threaten* or violen* or victimi?ation or revictimi?ation or re-victimi?ation)) ) 204

S16 TI ( (interpersonal N3 (abuse* or abusive or aggressi* or assault* or attack* or bully* or coerc* or cyberbully* or femicid* or harass* or homicid* or injur* or manipulate* or murder* or rape* or threaten* or violen* or victimi?ation or revictimi?ation or re-victimi?ation)) ) OR AB ( (interpersonal N3 (abuse* or abusive or aggressi* or assault* or attack* or bully* or coerc* or cyberbully* or femicid* or harass* or homicid* or injur* or manipulate* or murder* or rape* or threaten* or violen* or victimi?ation or revictimi?ation or re-victimi?ation)) ) 15

S15 TI ( ((boyfriend* or boy-friend* or girlfriend* or girl-friend*) N3 (abuse* or abusive or aggressi* or assault* or attack* or bully* or coerc* or cyberbully* or femicid* or harass* or homicid* or injur* or manipulate* or murder* or rape* or threaten* or violen* or victimi?ation or revictimi?ation or re-victimi?ation)) ) OR AB ( ((boyfriend* or boy-friend* or girlfriend* or girl-friend*) N3 (abuse* or abusive or aggressi* or assault* or attack* or bully* or coerc* or cyberbully* or femicid* or harass* or homicid* or injur* or manipulate* or murder* or rape* or threaten* or violen* or victimi?ation or revictimi?ation or re-victimi?ation)) ) 1

S14 TI ( ((relationship* or partner* or acquaintance* or non-stranger* or nonstranger*) N3 (abuse* or abusive or aggressi* or assault* or attack* or bully* or coerc* or cyberbully* or Femicid* or harass* or homicid* or injur* or manipulate* or murder* or rape* or threaten* or violen* or victimi?ation or revictimi?ation or re-victimi?ation)) ) OR AB ( ((relationship* or partner* or acquaintance* or non-stranger* or nonstranger*) N3 (abuse* or abusive or aggressi* or assault* or attack* or bully* or coerc* or cyberbully* or Femicid* or harass* or homicid* or injur* or manipulate* or murder* or rape* or threaten* or violen* or victimi?ation or revictimi?ation or re-victimi?ation)) ) 135

S13 TI ( ((date or dating) N3 (abuse* or abusive or aggressi* or assault* or attack* or bully* or coerc* or cyberbully* or femicid* or harass* or homicid* or injur* or manipulate* or murder* or rape* or threaten* or violen* or victimi?ation or revictimi?ation or re-victimi?ation)) ) OR AB ( ((date or dating) N3 (abuse* or abusive or aggressi* or assault* or attack* or bully* or coerc* or cyberbully* or femicid* or harass* or homicid* or injur* or manipulate* or murder* or rape* or threaten* or violen* or victimi?ation or revictimi?ation or re-victimi?ation)) ) 6

S12 TI "violence against women" OR AB "violence against women" 28

S11 TI ( (domestic N3 (abuse* or abusive or aggressi* or assault* or attack* or bully* or coerc* or cyberbully* or femicid* or harass* or homicid* or injur* or manipulate* or murder* or rape* or threaten* or violen* or victimi?ation or revictimi?ation or re-victimi?ation)) ) OR AB ( (domestic N3 (abuse* or abusive or aggressi* or assault* or attack* or bully* or coerc* or cyberbully* or femicid* or harass* or homicid* or injur* or manipulate* or murder* or rape* or threaten* or violen* or victimi?ation or revictimi?ation or re-victimi?ation)) ) 86

S10 TI SRGBV OR AB SRGBV 2

S9 TI GBV OR AB GBV 5

S8 TI (gender* N3 violen*) OR AB (gender* N3 violen*) 65

S7 TI IPV OR AB IPV 13

S6 TI rape* OR AB rape* 40

S5 TI ( (stalking or stalker*) ) OR AB ( (stalking or stalker*) ) 3

S4 DE "BIPHOBIA in schools" 2

S3 DE "HOMOPHOBIA in schools" OR DE "HOMOPHOBIA in high schools" 14

S2 DE "DOMESTIC violence" 55

S1 DE "INTIMATE violence" 1

**EBSCO Criminal Justice Abstracts**

**Search completed: 23/06/2020**

S64 S52 AND S63 1,999

S63 S53 OR S54 OR S55 OR S56 OR S57 OR S58 OR S59 OR S60 OR S61 OR S62 29,618

S62 TI ( classroom* or class-room* ) OR AB ( classroom* or class-room* ) 2,160

S61 TI ( pupil or pupils ) OR AB ( pupil or pupils ) 604

S60 TI school* OR AB school* 28,098

S59 (ZU "high school athletes") or (ZU "high school boys") or (ZU "high school girls") or (ZU "high school students") or (ZU "high schools") 1,553

S58 (ZU "junior high school students") or (ZU "junior high schools") 39

S57 (ZU "secondary school students") or (ZU "secondary schools") 51

S56 (ZU "middle school education") or (ZU "middle school students") or (ZU "middle schools") 584

S55 (ZU "elementary schools") 272

S54 (ZU "primary schools") 24

S53 (ZU "schools") 1,207

S52 S1 OR S2 OR S3 OR S4 OR S5 OR S6 OR S7 OR S8 OR S9 OR S10 OR S11 OR S12 OR S13 OR S14 OR S15 OR S16 OR S17 OR S18 OR S19 OR S20 OR S21 OR S22 OR S23 OR S24 OR S25 OR S26 OR S27 OR S28 OR S29 OR S30 OR S31 OR S32 OR S33 OR S34 OR S35 OR S36 OR S37 OR S38 OR S39 OR S40 OR S41 OR S42 OR S43 OR S44 OR S45 OR S46 OR S47 OR S48 OR S49 OR S50 OR S51 34,318

S51 TI (Benzies N2 Batchies) OR AB (Benzies N2 Batchies) 0

S50 TI (Benzies N2 Batchies) OR AB (Benzies N2 Batchies) 0

S49 TI DaVIPoP OR AB DaVIPoP 0

S48 TI "It's your game" OR AB "It's your game" 1

S47 TI SS-SSTP OR AB SS-SSTP 0

S46 TI SS-SSTP OR AB SS-SSTP 0

S45 TI "Second Step" OR AB "Second Step" 136

S44 TI "Expect Respect" OR AB "Expect Respect" 7

S43 TI "mentors in violence prevention" OR AB "mentors in violence prevention" 6

S42 TI "good schools toolkit" OR AB "good schools toolkit" 1

S41 TI "Teen choices" OR AB "Teen choices" 1

S40 TI "Shifting boundaries" OR AB "Shifting boundaries" 29

S39 TI "Safe Dates" OR AB "Safe Dates" 15

S38 TI "Fourth R" OR AB "Fourth R" 7

S37 TI TakeCARE OR AB TakeCARE 1

S36 TI ( ("Media Aware" or mediaaware) ) OR AB ( ("Media Aware" or mediaaware) ) 2

S35 TI "project respect" OR AB "project respect" 4

S34 TI ( (greendot or "green dot") ) OR AB ( (greendot or "green dot") ) 12

S33 TI "long live love" OR AB "long live love" 21

S32 TI "long live love" OR AB "long live love" 0

S31 TI ( ((LGB or LGBT* or homosexual* or lesbian* or gay or bisexual* or queer* or transgender* or transsexual) N3 (abuse* or abusive or aggressi* or assault* or attack* or bully* or coerc* or cyberbully* or femicid* or harass* or homicid* or injur* or manipulate* or murder* or rape* or threaten* or violen* or victimi?ation or revictimi?ation or re-victimi?ation)) ) OR AB ( ((LGB or LGBT* or homosexual* or lesbian* or gay or bisexual* or queer* or transgender* or transsexual) N3 (abuse* or abusive or aggressi* or assault* or attack* or bully* or coerc* or cyberbully* or femicid* or harass* or homicid* or injur* or manipulate* or murder* or rape* or threaten* or violen* or victimi?ation or revictimi?ation or re-victimi?ation)) ) 470

S30 TI ( (homophobi* or transphobi* or biphobi* or homonegativ*) ) OR AB ( (homophobi* or transphobi* or biphobi* or homonegativ*) ) 467

S29 TI ( (sext or sexts or sexting) ) OR AB ( (sext or sexts or sexting) ) 113

S28 TI ( (grope or groped or groping) ) OR AB ( (grope or groped or groping) ) 21

S27 TI ( ((coerc* or forced or unwanted or nonconsensual or non-consensual) N2 sex*) ) OR AB ( ((coerc* or forced or unwanted or nonconsensual or non-consensual) N2 sex*) ) 980

S26 TI ( (sexual* N3 (abusive or aggressi* or assault* or attack* or bully* or coerc* or cyberbully* or femicid* or harass* or homicid* or injur* or manipulate* or murder* or rape* or threaten* or violen* or victimi?ation or revictimi?ation or re-victimi?ation)) ) OR AB ( (sexual* N3 (abusive or aggressi* or assault* or attack* or bully* or coerc* or cyberbully* or femicid* or harass* or homicid* or injur* or manipulate* or murder* or rape* or threaten* or violen* or victimi?ation or revictimi?ation or re-victimi?ation)) ) 10,103

S25 TI ( (interpersonal N3 (abuse* or abusive or aggressi* or assault* or attack* or bully* or coerc* or cyberbully* or femicid* or harass* or homicid* or injur* or manipulate* or murder* or rape* or threaten* or violen* or victimi?ation or revictimi?ation or re-victimi?ation)) ) OR AB ( (interpersonal N3 (abuse* or abusive or aggressi* or assault* or attack* or bully* or coerc* or cyberbully* or femicid* or harass* or homicid* or injur* or manipulate* or murder* or rape* or threaten* or violen* or victimi?ation or revictimi?ation or re-victimi?ation)) ) 1,172

S24 TI ( ((boyfriend* or boy-friend* or girlfriend* or girl-friend*) N3 (abuse* or abusive or aggressi* or assault* or attack* or bully* or coerc* or cyberbully* or femicid* or harass* or homicid* or injur* or manipulate* or murder* or rape* or threaten* or violen* or victimi?ation or revictimi?ation or re-victimi?ation)) ) OR AB ( ((boyfriend* or boy-friend* or girlfriend* or girl-friend*) N3 (abuse* or abusive or aggressi* or assault* or attack* or bully* or coerc* or cyberbully* or femicid* or harass* or homicid* or injur* or manipulate* or murder* or rape* or threaten* or violen* or victimi?ation or revictimi?ation or re-victimi?ation)) ) 73

S23 TI ( ((relationship* or partner* or acquaintance* or non-stranger* or nonstranger*) N3 (abuse* or abusive or aggressi* or assault* or attack* or bully* or coerc* or cyberbully* or Femicid* or harass* or homicid* or injur* or manipulate* or murder* or rape* or threaten* or violen* or victimi?ation or revictimi?ation or re-victimi?ation)) ) OR AB ( ((relationship* or partner* or acquaintance* or non-stranger* or nonstranger*) N3 (abuse* or abusive or aggressi* or assault* or attack* or bully* or coerc* or cyberbully* or Femicid* or harass* or homicid* or injur* or manipulate* or murder* or rape* or threaten* or violen* or victimi?ation or revictimi?ation or re-victimi?ation)) ) 10,089

S22 TI ( ((date or dating) N3 (abuse* or abusive or aggressi* or assault* or attack* or bully* or coerc* or cyberbully* or femicid* or harass* or homicid* or injur* or manipulate* or murder* or rape* or threaten* or violen* or victimi?ation or revictimi?ation or re-victimi?ation)) ) OR AB ( ((date or dating) N3 (abuse* or abusive or aggressi* or assault* or attack* or bully* or coerc* or cyberbully* or femicid* or harass* or homicid* or injur* or manipulate* or murder* or rape* or threaten* or violen* or victimi?ation or revictimi?ation or re-victimi?ation)) ) 1,269

S21 TI "violence against women" OR AB "violence against women" 1,959

S20 TI ( (domestic N3 (abuse* or abusive or aggressi* or assault* or attack* or bully* or coerc* or cyberbully* or femicid* or harass* or homicid* or injur* or manipulate* or murder* or rape* or threaten* or violen* or victimi?ation or revictimi?ation or re-victimi?ation)) ) OR AB ( (domestic N3 (abuse* or abusive or aggressi* or assault* or attack* or bully* or coerc* or cyberbully* or femicid* or harass* or homicid* or injur* or manipulate* or murder* or rape* or threaten* or violen* or victimi?ation or revictimi?ation or re-victimi?ation)) ) 6,529

S19 TI SRGBV OR AB SRGBV 0

S18 TI SRGBV OR AB SRGBV 0

S17 TI GBV OR AB GBV 56

S16 TI (gender* N3 violen*) OR AB (gender* N3 violen*) 1,259

S15 TI IPV OR AB IPV 2,487

S14 TI rape* OR AB rape* 4,708

S13 TI ( (stalking or stalker*) ) OR AB ( (stalking or stalker*) ) 963

S12 (ZU "transphobia") or (ZU "transphobia in schools") 19

S11 (ZU "biphobia") 4

S10 (ZU "homophobia") or (ZU "homophobia in high schools") or (ZU "homophobia in schools") 279

S9 (ZU "sexual harassment") or (ZU "sexual harassment in education") or (ZU "sexual harassment in education -- prevention") 844

S8 (ZU "domestic violence") 2,425

S7 (ZU "relationship abuse") 19

S6 (ZU "victims of dating violence") or (ZU "victims of domestic violence") 851

S5 (ZU "dating violence") 724

S4 (ZU "sex crimes") or (ZU "sex crimes -- prevention") 6,457

S3 (ZU "rape") 2,277

S2 (ZU "stalking") or (ZU "stalking -- prevention") 547

S1 (ZU "intimate partner violence") 3,675

**EBSCO Econlit (1886-)**

**Search completed: 23/06/2020**

**S42 S36 AND S41 127**

S41 S37 OR S38 OR S39 OR S40 40,965

S40 (ZW "school") 1,475

S39 AB ( ( classroom* or class-room* ) ) OR TI ( ( classroom* or class-room* ) ) 1,756

S38 AB ( ( pupil or pupils ) ) OR TI ( ( pupil or pupils ) ) 1,012

S37 AB school* OR TI school* OR SO school* 39,699

S36 S1 OR S2 OR S3 OR S4 OR S5 OR S6 OR S7 OR S8 OR S9 OR S10 OR S11 OR S12 OR S13 OR S14 OR S15 OR S16 OR S17 OR S18 OR S19 OR S20 OR S21 OR S22 OR S23 OR S24 OR S25 OR S26 OR S27 OR S28 OR S29 OR S30 OR S31 OR S32 OR S33 OR S34 OR S35 2,729

S35 AB (Benzies N2 Batchies) OR TI (Benzies N2 Batchies) 0

S34 AB DaVIPoP OR TI DaVIPoP 0

S33 AB "It's your game" OR TI "It's your game" 0

S32 AB SS-SSTP OR TI SS-SSTP 0

S31 AB "Second Step" OR TI "Second Step" 968

S30 AB "Expect Respect" OR TI "Expect Respect" 0

S29 AB "mentors in violence prevention" OR TI "mentors in violence prevention" 0

S28 AB "good schools toolkit" OR TI "good schools toolkit" 0

S27 AB "Teen choices" OR TI "Teen choices" 0

S26 AB "Shifting boundaries" OR TI "Shifting boundaries" 41

S25 AB "Safe Dates" OR TI "Safe Dates" 0

S24 AB "Fourth R" OR TI "Fourth R" 3

S23 AB TakeCARE OR TI TakeCARE 0

S22 AB ( ("Media Aware" or mediaaware) ) OR TI ( ("Media Aware" or mediaaware) ) 0

S21 AB "project respect" OR TI "project respect" 0

S20 AB ( (greendot or "green dot") ) OR TI ( (greendot or "green dot") ) 5

S19 AB "long live love" OR TI "long live love" 0

S18 AB ( ((LGB or LGBT* or homosexual* or lesbian* or gay or bisexual* or queer* or transgender* or transsexual) N3 (abuse* or abusive or aggressi* or assault* or attack* or bully* or coerc* or cyberbully* or femicid* or harass* or homicid* or injur* or manipulate* or murder* or rape* or threaten* or violen* or victimi?ation or revictimi?ation or re-victimi?ation)) ) OR TI ( ((LGB or LGBT* or homosexual* or lesbian* or gay or bisexual* or queer* or transgender* or transsexual) N3 (abuse* or abusive or aggressi* or assault* or attack* or bully* or coerc* or cyberbully* or femicid* or harass* or homicid* or injur* or manipulate* or murder* or rape* or threaten* or violen* or victimi?ation or revictimi?ation or re-victimi?ation)) ) 14

S17 AB ( (homophobi* or transphobi* or biphobi* or homonegativ*) ) OR TI ( (homophobi* or transphobi* or biphobi* or homonegativ*) ) 22

S16 AB ( (sext or sexts or sexting) ) OR TI ( (sext or sexts or sexting) ) 1

S15 AB ( (grope or groped or groping) ) OR TI ( (grope or groped or groping) ) 40

S14 AB ( (coerc* or forced or unwanted or nonconsensual or non-consensual) N2 sex*) ) OR TI ( (coerc* or forced or unwanted or nonconsensual or non-consensual) N2 sex*) ) 20

S13 AB ( ( (sexual* N3 (abusive or aggressi* or assault* or attack* or bully* or coerc* or cyberbully* or femicid* or harass* or homicid* or injur* or manipulate* or murder* or rape* or threaten* or violen* or victimi?ation or revictimi?ation or re-victimi?ation)) ) OR TI ( ( (sexual* N3 (abusive or aggressi* or assault* or attack* or bully* or coerc* or cyberbully* or femicid* or harass* or homicid* or injur* or manipulate* or murder* or rape* or threaten* or violen* or victimi?ation or revictimi?ation or re-victimi?ation)) ) 313

S12 AB ( ( (interpersonal N3 (abuse* or abusive or aggressi* or assault* or attack* or bully* or coerc* or cyberbully* or femicid* or harass* or homicid* or injur* or manipulate* or murder* or rape* or threaten* or violen* or victimi?ation or revictimi?ation or re-victimi?ation)) ) OR TI ( ( (interpersonal N3 (abuse* or abusive or aggressi* or assault* or attack* or bully* or coerc* or cyberbully* or femicid* or harass* or homicid* or injur* or manipulate* or murder* or rape* or threaten* or violen* or victimi?ation or revictimi?ation or re-victimi?ation)) ) 36

S11 AB ( ((boyfriend* or boy-friend* or girlfriend* or girl-friend*) N3 (abuse* or abusive or aggressi* or assault* or attack* or bully* or coerc* or cyberbully* or femicid* or harass* or homicid* or injur* or manipulate* or murder* or rape* or threaten* or violen* or victimi?ation or revictimi?ation or re-victimi?ation)) ) OR TI ( ((boyfriend* or boy-friend* or girlfriend* or girl-friend*) N3 (abuse* or abusive or aggressi* or assault* or attack* or bully* or coerc* or cyberbully* or femicid* or harass* or homicid* or injur* or manipulate* or murder* or rape* or threaten* or violen* or victimi?ation or revictimi?ation or re-victimi?ation)) ) 0

S10 AB ( ((relationship* or partner* or acquaintance* or non-stranger* or nonstranger*) N3 (abuse* or abusive or aggressi* or assault* or attack* or bully* or coerc* or cyberbully* or femicid* or harass* or homicid* or injur* or manipulate* or murder* or rape* or threaten* or violen* or victimi?ation or revictimi?ation or re-victimi?ation)) ) OR TI ( ((relationship* or partner* or acquaintance* or non-stranger* or nonstranger*) N3 (abuse* or abusive or aggressi* or assault* or attack* or bully* or coerc* or cyberbully* or femicid* or harass* or homicid* or injur* or manipulate* or murder* or rape* or threaten* or violen* or victimi?ation or revictimi?ation or re-victimi?ation)) ) 421

S9 AB ( ((date or dating) N3 (abuse* or abusive or aggressi* or assault* or attack* or bully* or coerc* or cyberbully* or femicid* or harass* or homicid* or injur* or manipulate* or murder* or rape* or threaten* or violen* or victimi?ation or revictimi?ation or re-victimi?ation)) ) OR TI ( ((date or dating) N3 (abuse* or abusive or aggressi* or assault* or attack* or bully* or coerc* or cyberbully* or femicid* or harass* or homicid* or injur* or manipulate* or murder* or rape* or threaten* or violen* or victimi?ation or revictimi?ation or re-victimi?ation)) ) 16

S8 AB "violence against women" OR TI "violence against women" 168

S7 AB ( (domestic N3 (abuse* or abusive or aggressi* or assault* or attack* or bully* or coerc* or cyberbully* or femicid* or harass* or homicid* or injur* or manipulate* or murder* or rape* or threaten* or violen* or victimi?ation or revictimi?ation or re-victimi?ation)) ) OR TI ( (domestic N3 (abuse* or abusive or aggressi* or assault* or attack* or bully* or coerc* or cyberbully* or femicid* or harass* or homicid* or injur* or manipulate* or murder* or rape* or threaten* or violen* or victimi?ation or revictimi?ation or re-victimi?ation)) ) 483

S6 AB SRGBV OR TI SRGBV 0

S5 AB GBV OR TI GBV 10

S4 AB (gender* N3 violen*) OR TI (gender* N3 violen*) 143

S3 AB (IPV OR intimate partner violence) OR TI (IPV OR intimate partner violence) 160

S2 AB rape* OR TI rape* 300

S1 AB ( stalking or stalker* ) OR TI ( stalking or stalker* ) 19

**EBSCO Education Research Complete**

**Search completed: 23/06/2020**

S46 S38 AND S45 5,809

S45 S39 OR S40 OR S41 OR S42 OR S43 OR S44

S44 TI ( classroom* or class-room* ) OR AB ( classroom* or class-room* )

S43 TI ( pupil or pupils ) OR AB ( pupil or pupils )

S42 TI school* OR AB school* OR SO school*

S41 DE "BOARDING school students" OR DE "MIDDLE school students" OR DE "PRIVATE school students" OR DE "SCHOOL children" OR DE "SECONDARY school students" OR DE "SIXTH form students"

S40 DE "SCHOOL health services"

S39 DE "SCHOOLS" OR DE "BOARDING schools" OR DE "BRITISH schools" OR DE "DAY schools" OR DE "DISADVANTAGED schools" OR DE "ELEMENTARY schools" OR DE "FAILING schools" OR DE "PRIMARY schools" OR DE "PRIVATE schools" OR DE "PUBLIC schools" OR DE "RURAL schools" OR DE "SECONDARY schools" OR DE "SINGLE sex schools" OR DE "TRADITIONAL schools"

S38 S1 OR S2 OR S3 OR S4 OR S5 OR S6 OR S7 OR S8 OR S9 OR S10 OR S11 OR S12 OR S13 OR S14 OR S15 OR S16 OR S17 OR S18 OR S19 OR S20 OR S21 OR S22 OR S23 OR S24 OR S25 OR S26 OR S27 OR S28 OR S29 OR S30 OR S31 OR S32 OR S33 OR S34 OR S35 OR S36 OR S37 27,126

S37 TI (Benzies N2 Batchies) OR AB (Benzies N2 Batchies)

S36 TI DaVIPoP OR AB DaVIPoP

S35 TI "It's your game" OR AB "It's your game"

S34 TI SS-SSTP OR AB SS-SSTP

S33 TI "Second Step" OR AB "Second Step"

S32 TI "Expect Respect" OR AB "Expect Respect"

S31 TI "mentors in violence prevention" OR AB "mentors in violence prevention"

S30 TI "good schools toolkit" OR AB "good schools toolkit"

S29 TI "Teen choices" OR AB "Teen choices"

S28 TI "Shifting boundaries" OR AB "Shifting boundaries"

S27 TI "Safe Dates" OR AB "Safe Dates"

S26 TI "Fourth R" OR AB "Fourth R"

S25 TI TakeCARE OR AB TakeCARE

S24 TI ( ("Media Aware" or mediaaware) ) OR AB ( ("Media Aware" or mediaaware) )

S23 TI "project respect" OR AB "project respect"

S22 TI ( (greendot or "green dot") ) OR AB ( (greendot or "green dot") )

S21 TI "long live love" OR AB "long live love"

S20 TI ( ((LGB or LGBT* or homosexual* or lesbian* or gay or bisexual* or queer* or transgender* or transsexual*) N3 (abuse* or abusive or aggressi* or assault* or attack* or bully* or coerc* or cyberbully* or Femicid* or harass* or homicid* or injur* or manipulate* or murder* or rape* or threaten* or violen* or victimi?ation or revictimi?ation or re-victimi?ation)) ) OR AB ( ((LGB or LGBT* or homosexual* or lesbian* or gay or bisexual* or queer* or transgender* or transsexual*) N3 (abuse* or abusive or aggressi* or assault* or attack* or bully* or coerc* or cyberbully* or Femicid* or harass* or homicid* or injur* or manipulate* or murder* or rape* or threaten* or violen* or victimi?ation or revictimi?ation or re-victimi?ation)) ) 807

S19 TI ( (homophobi* or transphobi* or biphobi* or homonegativ*) ) OR AB ( (homophobi* or transphobi* or biphobi* or homonegativ*) )

S18 TI ( (sext or sexts or sexting) ) OR AB ( (sext or sexts or sexting) )

S17 TI ( (grope or groped or groping) ) OR AB ( (grope or groped or groping) )

S16 TI ( ((coerc* or forced or unwanted or nonconsensual or non-consensual) N2 sex*) ) OR AB ( ((coerc* or forced or unwanted or nonconsensual or non-consensual) N2 sex*) )

S15 TI ( (sexual* N3 (abusive or aggressi* or assault* or attack* or bully* or coerc* or cyberbully* or femicid* or harass* or homicid* or injur* or manipulate* or murder* or rape* or threaten* or violen* or victimi?ation or revictimi?ation or re-victimi?ation)) ) OR AB ( (sexual* N3 (abusive or aggressi* or assault* or attack* or bully* or coerc* or cyberbully* or femicid* or harass* or homicid* or injur* or manipulate* or murder* or rape* or threaten* or violen* or victimi?ation or revictimi?ation or re-victimi?ation)) )

S14 TI ( (interpersonal N3 (abuse* or abusive or aggressi* or assault* or attack* or bully* or coerc* or cyberbully* or femicid* or harass* or homicid* or injur* or manipulate* or murder* or rape* or threaten* or violen* or victimi?ation or revictimi?ation or re-victimi?ation)) ) OR AB ( (interpersonal N3 (abuse* or abusive or aggressi* or assault* or attack* or bully* or coerc* or cyberbully* or femicid* or harass* or homicid* or injur* or manipulate* or murder* or rape* or threaten* or violen* or victimi?ation or revictimi?ation or re-victimi?ation)) )

S13 TI ( ((boyfriend* or boy-friend* or girlfriend* or girl-friend*) N3 (abuse* or abusive or aggressi* or assault* or attack* or bully* or coerc* or cyberbully* or femicid* or harass* or homicid* or injur* or manipulate* or murder* or rape* or threaten* or violen* or victimi?ation or revictimi?ation or re-victimi?ation)) ) OR AB ( ((boyfriend* or boy-friend* or girlfriend* or girl-friend*) N3 (abuse* or abusive or aggressi* or assault* or attack* or bully* or coerc* or cyberbully* or femicid* or harass* or homicid* or injur* or manipulate* or murder* or rape* or threaten* or violen* or victimi?ation or revictimi?ation or re-victimi?ation)) )

S12 TI ( ((relationship* or partner* or acquaintance* or non-stranger* or nonstranger*) N3 (abuse* or abusive or aggressi* or assault* or attack* or bully* or coerc* or cyberbully* or Femicid* or harass* or homicid* or injur* or manipulate* or murder* or rape* or threaten* or violen* or victimi?ation or revictimi?ation or re-victimi?ation)) ) OR AB ( ((relationship* or partner* or acquaintance* or non-stranger* or nonstranger*) N3 (abuse* or abusive or aggressi* or assault* or attack* or bully* or coerc* or cyberbully* or Femicid* or harass* or homicid* or injur* or manipulate* or murder* or rape* or threaten* or violen* or victimi?ation or revictimi?ation or re-victimi?ation)) )

S11 TI ( ((date or dating) N3 (abuse* or abusive or aggressi* or assault* or attack* or bully* or coerc* or cyberbully* or femicid* or harass* or homicid* or injur* or manipulate* or murder* or rape* or threaten* or violen* or victimi?ation or revictimi?ation or re-victimi?ation)) ) OR AB ( ((date or dating) N3 (abuse* or abusive or aggressi* or assault* or attack* or bully* or coerc* or cyberbully* or femicid* or harass* or homicid* or injur* or manipulate* or murder* or rape* or threaten* or violen* or victimi?ation or revictimi?ation or re-victimi?ation)) )

S10 TI "violence against women" OR AB "violence against women"

S9 TI ( (domestic N3 (abuse* or abusive or aggressi* or assault* or attack* or bully* or coerc* or cyberbully* or femicid* or harass* or homicid* or injur* or manipulate* or murder* or rape* or threaten* or violen* or victimi?ation or revictimi?ation or re-victimi?ation)) ) OR AB ( (domestic N3 (abuse* or abusive or aggressi* or assault* or attack* or bully* or coerc* or cyberbully* or femicid* or harass* or homicid* or injur* or manipulate* or murder* or rape* or threaten* or violen* or victimi?ation or revictimi?ation or re-victimi?ation)) )

S8 TI SRGBV OR AB SRGBV

S7 TI GBV OR AB GBV

S6 TI (gender* N3 violen*) OR AB (gender* N3 violen*)

S5 TI IPV OR AB IPV

S4 TI rape* OR AB rape*

S3 TI ( (stalking or stalker*) ) OR AB ( (stalking or stalker*) )

S2 DE "HOMOPHOBIA in schools" OR DE "HOMOPHOBIA in high schools" OR DE "BIPHOBIA in schools"

S1 DE "SEXUAL harassment in education"

**EBSCO ERIC (1966-)**

**Search completed: 23/06/2020**

S45 S37 AND S44 3,795

S44 S38 OR S39 OR S40 OR S41 OR S42 OR S43 703,058

S43 TI ( classroom* or class-room* ) OR AB ( classroom* or class-room* ) 179,174

S42 TI ( pupil or pupils ) OR AB ( pupil or pupils ) 27,575

S41 TI school* OR AB school* OR SO school* 551,810

S40 DE "Elementary School Students" OR DE "Middle School Students" OR DE "Secondary School Students" OR DE "High School Students" OR DE "Junior High School Students" 112,870

S39 DE "School Health Services" 2,396

S38 DE "Schools" OR DE "Boarding Schools" OR DE "Residential Schools" OR DE "Disadvantaged Schools" OR DE "Elementary Schools" OR DE "Middle Schools" OR DE "Nursery Schools" OR DE "Private Schools" OR DE "Public Schools" OR DE "Regional Schools" OR DE "Rural Schools" OR DE "Secondary Schools" OR DE "High Schools" OR DE "Junior High Schools" OR DE "Single Sex Schools" OR DE "Slum Schools" OR DE "Small Schools" OR DE "State Schools" OR DE "Suburban Schools" OR DE "Traditional Schools" OR DE "Urban Schools" 180,906

S37 S1 OR S2 OR S3 OR S4 OR S5 OR S6 OR S7 OR S8 OR S9 OR S10 OR S11 OR S12 OR S13 OR S14 OR S15 OR S16 OR S17 OR S18 OR S19 OR S20 OR S21 OR S22 OR S23 OR S24 OR S25 OR S26 OR S27 OR S28 OR S29 OR S30 OR S31 OR S32 OR S33 OR S34 OR S35 OR S36 10,646

S36 TI (Benzies N2 Batchies) OR AB (Benzies N2 Batchies) 0

S35 TI DaVIPoP OR AB DaVIPoP 0

S34 TI "It's your game" OR AB "It's your game" 2

S33 TI SS-SSTP OR AB SS-SSTP 2

S32 TI "Second Step" OR AB "Second Step" 462

S31 TI "Expect Respect" OR AB "Expect Respect" 9

S30 TI "mentors in violence prevention OR AB "mentors in violence prevention 2

S29 TI "Teen choices" OR AB "Teen choices" 2

S28 TI "Shifting boundaries" OR AB "Shifting boundaries" 33

S27 TI "Safe Dates" OR AB "Safe Dates" 2

S26 TI "Fourth R" OR AB "Fourth R" 78

S25 TI TakeCARE OR AB TakeCARE 1

S24 TI ( ("Media Aware" or mediaaware) ) OR AB ( ("Media Aware" or mediaaware) ) 3

S23 TI "project respect" OR AB "project respect" 5

S22 TI ( (greendot or "green dot" ) OR AB ( (greendot or "green dot" ) 20

S21 TI "long live love" OR AB "long live love" 1

S20 TI ( ((LGB or LGBT* or homosexual* or lesbian* or gay or bisexual* or queer* or transgender* or transsexual) N3 (abuse* or abusive or aggressi* or assault* or attack* or bully* or coerc* or cyberbully* or femicid* or harass* or homicid* or injur* or manipulate* or murder* or rape* or threaten* or violen* or victimi?ation or revictimi?ation or re-victimi?ation)) ) OR AB ( ((LGB or LGBT* or homosexual* or lesbian* or gay or bisexual* or queer* or transgender* or transsexual) N3 (abuse* or abusive or aggressi* or assault* or attack* or bully* or coerc* or cyberbully* or femicid* or harass* or homicid* or injur* or manipulate* or murder* or rape* or threaten* or violen* or victimi?ation or revictimi?ation or re-victimi?ation)) ) 269

S19 TI ( (homophobi* or transphobi* or biphobi* or homonegativ*) ) OR AB ( (homophobi* or transphobi* or biphobi* or homonegativ*) ) 917

S18 TI ( (sext or sexts or sexting) ) OR AB ( (sext or sexts or sexting) ) 62

S17 TI ( (grope or groped or groping) ) OR AB ( (grope or groped or groping) ) 46

S16 TI ( ((coerc* or forced or unwanted or nonconsensual or non-consensual) N2 sex*) ) OR AB ( ((coerc* or forced or unwanted or nonconsensual or non-consensual) N2 sex*) ) 348

S15 TI ( (sexual* N3 (abusive or aggressi* or assault* or attack* or bully* or coerc* or cyberbully* or femicid* or harass* or homicid* or injur* or manipulate* or murder* or rape* or threaten* or violen* or victimi?ation or revictimi?ation or re-victimi?ation)) ) OR AB ( (sexual* N3 (abusive or aggressi* or assault* or attack* or bully* or coerc* or cyberbully* or femicid* or harass* or homicid* or injur* or manipulate* or murder* or rape* or threaten* or violen* or victimi?ation or revictimi?ation or re-victimi?ation)) ) 3,728

S14 TI ( (interpersonal N3 (abuse* or abusive or aggressi* or assault* or attack* or bully* or coerc* or cyberbully* or femicid* or harass* or homicid* or injur* or manipulate* or murder* or rape* or threaten* or violen* or victimi?ation or revictimi?ation or re-victimi?ation)) ) OR AB ( (interpersonal N3 (abuse* or abusive or aggressi* or assault* or attack* or bully* or coerc* or cyberbully* or femicid* or harass* or homicid* or injur* or manipulate* or murder* or rape* or threaten* or violen* or victimi?ation or revictimi?ation or re-victimi?ation)) ) 385

S13 TI ( ((boyfriend* or boy-friend* or girlfriend* or girl-friend*) N3 (abuse* or abusive or aggressi* or assault* or attack* or bully* or coerc* or cyberbully* or femicid* or harass* or homicid* or injur* or manipulate* or murder* or rape* or threaten* or violen* or victimi?ation or revictimi?ation or re-victimi?ation)) ) OR AB ( ((boyfriend* or boy-friend* or girlfriend* or girl-friend*) N3 (abuse* or abusive or aggressi* or assault* or attack* or bully* or coerc* or cyberbully* or femicid* or harass* or homicid* or injur* or manipulate* or murder* or rape* or threaten* or violen* or victimi?ation or revictimi?ation or re-victimi?ation)) ) 28

S12 TI ( ((relationship* or partner* or acquaintance* or non-stranger* or nonstranger*) N3 (abuse* or abusive or aggressi* or assault* or attack* or bully* or coerc* or cyberbully* or Femicid* or harass* or homicid* or injur* or manipulate* or murder* or rape* or threaten* or violen* or victimi?ation or revictimi?ation or re-victimi?ation)) ) OR AB ( ((relationship* or partner* or acquaintance* or non-stranger* or nonstranger*) N3 (abuse* or abusive or aggressi* or assault* or attack* or bully* or coerc* or cyberbully* or Femicid* or harass* or homicid* or injur* or manipulate* or murder* or rape* or threaten* or violen* or victimi?ation or revictimi?ation or re-victimi?ation)) ) 2,659

S11 TI ( ((date or dating) N3 (abuse* or abusive or aggressi* or assault* or attack* or bully* or coerc* or cyberbully* or femicid* or harass* or homicid* or injur* or manipulate* or murder* or rape* or threaten* or violen* or victimi?ation or revictimi?ation or re-victimi?ation)) ) OR AB ( ((date or dating) N3 (abuse* or abusive or aggressi* or assault* or attack* or bully* or coerc* or cyberbully* or femicid* or harass* or homicid* or injur* or manipulate* or murder* or rape* or threaten* or violen* or victimi?ation or revictimi?ation or re-victimi?ation)) ) 570

S10 TI "violence against women" OR AB "violence against women" 255

S9 TI ( (domestic N3 (abuse* or abusive or aggressi* or assault* or attack* or bully* or coerc* or cyberbully* or femicid* or harass* or homicid* or injur* or manipulate* or murder* or rape* or threaten* or violen* or victimi?ation or revictimi?ation or re-victimi?ation)) ) OR AB ( (domestic N3 (abuse* or abusive or aggressi* or assault* or attack* or bully* or coerc* or cyberbully* or femicid* or harass* or homicid* or injur* or manipulate* or murder* or rape* or threaten* or violen* or victimi?ation or revictimi?ation or re-victimi?ation)) ) 1,190

S8 TI SRGBV OR AB SRGBV 8

S7 TI GBV OR AB GBV 12

S6 TI gender* N3 violen* OR AB gender* N3 violen* 300

S5 IPV OR IPV 290

S4 TI rape* OR AB rape* 1,211

S3 TI ( stalking or stalker* ) OR AB ( stalking or stalker* ) 179

S2 DE "Sexual Harassment" 1,667

S1 DE "Rape" 1,275

**CENTRAL and the Cochrane Database of Systematic Reviews via the Cochrane Library (Issue 6 of 12, 2020)**

**Search completed: 23/06/2020**

#1 MeSH descriptor: [Intimate Partner Violence] explode all trees

#2 MeSH descriptor: [Gender-Based Violence] explode all trees

#3 MeSH descriptor: [Stalking] explode all trees

#4 MeSH descriptor: [Rape] explode all trees

#5 MeSH descriptor: [Sex Offenses] explode all trees

#6 MeSH descriptor: [Battered Women] explode all trees

#7 MeSH descriptor: [Spouse Abuse] explode all trees

#8 MeSH descriptor: [Coercion] explode all trees

#9 MeSH descriptor: [Domestic Violence] explode all trees

#10 MeSH descriptor: [Homophobia] explode all trees

#11 (stalking or stalker*):ti,ab,kw

#12 rape*:ti,ab,kw

#13 "intimate partner violence":ti,ab,kw

#14 IPV:ti,ab,kw

#15 (gender* near/3 violen*):ti,ab,kw

#16 GBV:ti,ab,kw

#17 SRGBV:ti,ab,kw

#18 "violence against women":ti,ab,kw

#19 (domestic near/3 (abuse* or abusive or aggressi* or assault* or attack* or bully* or coerc* or cyberbully* or femicid* or harass* or homicid* or injur* or manipulat* or murder* or rape* or threaten* or violen* or victimisation or victimization or revictimisation or revictimization or re-victimisation or re-victimization)):ti,ab,kw

#20 ((date or dating) near/3 (abuse* or abusive or aggressi* or assault* or attack* or bully* or coerc* or cyberbully* or femicid* or harass* or homicid* or injur* or manipulat* or murder* or rape* or threaten* or violen* or victimisation or victimization or revictimisation or revictimization or re-victimisation or re-victimization)):ti,ab,kw

#21 ((relationship* or partner* or acquaintance* or non-stranger* or nonstranger*) near/3 (abuse* or abusive or aggressi* or assault* or attack* or bully* or coerc* or cyberbully* or femicid* or harass* or homicid* or injur* or manipulat* or murder* or rape* or threaten* or violen* or victimisation or victimization or revictimisation or revictimization or re-victimisation or re-victimization)):ti,ab,kw

#22 ((boyfriend* or boy-friend* or girlfriend* or girl-friend*) near/3 (abuse* or abusive or aggressi* or assault* or attack* or bully* or coerc* or cyberbully* or femicid* or harass* or homicid* or injur* or manipulat* or murder* or rape* or threaten* or violen* or victimisation or victimization or revictimisation or revictimization or re-victimisation or re-victimization)):ti,ab,kw

#23 (interpersonal near/3 (abuse* or abusive or aggress* or assault* or attack or coerc* or cyberbully* or femicid* or harass* or homicid* or injur* or manipulat* or murder* or rape* or threaten* or violen* or victimization or revictimisation or revictimization or re-victimisation or re-victimization)):ti,ab,kw

#24 (sexual* near/2 (aggressi* or assault* or attack or coerc* or cyberbully* or femicid* or harass* or homicid* or injur* or manipulat* or murder* or rape* or threaten* or violen* or victimization or revictimisation or revictimization or re-victimisation or re-victimization)):ti,ab,kw

#25 ((coerc* or forced or unwanted or nonconsensual or non-consensual) near/2 sex*):ti,ab,kw

#26 (grope or groped or groping):ti,ab,kw

#27 (sext or sexts or sexting):ti,ab,kw

#28 (homophobi* or transphobi* or biphobi* or homonegativ*):ti,ab,kw

#29 ((LGB or LGBT* or homosexual* or lesbian* or gay or bisexual* or queer* or transgender* or transsexual) near/3 (abuse* or abusive or aggressi* or assault* or attack* or bully* or coerc* or cyberbully* or femicid* or harass* or homicid* or injur* or manipulate* or murder* or rape* or threaten* or violen* or victimisation or victimization or revictimisation or revictimization or re-victimisation or re-victimization)):ti,ab,kw

#30 "long live love":ti,ab,kw

#31 (greendot or "green dot"):ti,ab,kw

#32 "project respect":ti,ab,kw

#33 ("Media Aware" or mediaaware):ti,ab,k**w**

#34 TakeCARE:ti,ab,kw

#35 "Fourth R":ti,ab,kw

#36 "Safe Dates":ti,ab,kw

#37 "Shifting boundaries":ti,ab,kw

#38 "Teen choices":ti,ab,kw

#39 "good schools toolkit":ti,ab,kw

#40 "mentors in violence prevention":ti,ab,kw

#41 "Expect Respect":ti,ab,kw

#42 "Second Step":ti,ab,kw

#43 SS-SSTP:ti,ab,kw

#44 "It's your game":ti,ab,kw

#45 DaVIPoP:ti,ab,kw

#46 (Benzies near/2 Batchies):ti,ab,kw

#47 {OR #1-#46}

#48 MeSH descriptor: [Schools] explode all trees

#49 MeSH descriptor: [School Health Services] explode all trees

#50 MeSH descriptor: [Students] this term only

#51 MeSH descriptor: [Curriculum] explode all trees

#52 (school*):ti,ab,kw

#53 (pupil or pupils):ti,ab,kw

#54 (classroom* or class-room*):ti,ab,kw

#55 {OR #48-#54}

#56 #47 AND #55

**ProQuest ASSIA (Applied Social Sciences Index and Abstracts) (1987-)**

**Search completed: 23/06/2020**

**(**MAINSUBJECT.EXACT("Gender violence") OR MAINSUBJECT.EXACT.EXPLODE("Rape") OR MAINSUBJECT.EXACT("Sexual harassment") OR MAINSUBJECT.EXACT("Sexual violence") OR MAINSUBJECT.EXACT("Stalking") OR MAINSUBJECT.EXACT("Battered women") OR MAINSUBJECT.EXACT("Domestic violence") OR MAINSUBJECT.EXACT("Coercion") OR MAINSUBJECT.EXACT("Homophobia") OR ti(stalking OR stalker* OR rape* OR "intimate partner violence" OR IPV OR (gender NEAR/3 violen*) OR GBV OR SRGBV OR "violence against women" OR ((domestic OR date OR dating OR relationship* OR partner* OR acquaintance* OR non-stranger* OR nonstranger* OR boyfriend* OR boy-friend* OR girlfriend* OR girl-friend* OR interpersonal OR sexual* OR LGB OR LGBT* OR homosexual* OR lesbian* OR gay OR bisexual* OR queer* OR transgender* OR transsexual) NEAR/3 (abuse* OR abusive OR aggressi* OR assault* OR attack* OR bully* OR coerc* OR cyberbully* OR femicid* OR harass* OR homicid* OR injur* OR manipulate* OR murder* OR rape* OR threaten* OR violen* OR victimi?ation OR revictimi?ation OR re-victimi?ation)) OR ((coerc* OR forced OR unwanted OR nonconsensual OR non-consensual) AND sex*) OR grope OR groping OR groped OR sext OR sexts OR sexting OR homophobi* OR transphobi* OR biphobi* OR homonegativ* OR ("long live love" OR "green dot" OR greendot OR "project respect" OR "Media Aware" OR mediaaware OR TakeCARE OR "Fourth R" OR "Safe Dates" OR "Shifting boundaries" OR "Teen choices" OR "mentors in violence prevention" OR "Expect Respect" OR "Second Step" OR SS-SSTP OR "It's your game" OR DaVIPoP OR (Benzies N2 batchies))) OR ab(stalking OR stalker* OR rape* OR "intimate partner violence" OR IPV OR (gender NEAR/3 violen*) OR GBV OR SRGBV OR "violence against women" OR ((domestic OR date OR dating OR relationship* OR partner* OR acquaintance* OR non-stranger* OR nonstranger* OR boyfriend* OR boy-friend* OR girlfriend* OR girl-friend* OR interpersonal OR sexual* OR LGB OR LGBT* OR homosexual* OR lesbian* OR gay OR bisexual* OR queer* OR transgender* OR transsexual) NEAR/3 (abuse* OR abusive OR aggressi* OR assault* OR attack* OR bully* OR coerc* OR cyberbully* OR femicid* OR harass* OR homicid* OR injur* OR manipulate* OR murder* OR rape* OR threaten* OR violen* OR victimi?ation OR revictimi?ation OR re-victimi?ation)) OR ((coerc* OR forced OR unwanted OR nonconsensual OR non-consensual) AND sex*) OR grope OR groping OR groped OR sext OR sexts OR sexting OR homophobi* OR transphobi* OR biphobi* OR homonegativ* OR ("long live love" OR "green dot" OR greendot OR "project respect" OR "Media Aware" OR mediaaware OR TakeCARE OR "Fourth R" OR "Safe Dates" OR "Shifting boundaries" OR "Teen choices" OR "mentors in violence prevention" OR "Expect Respect" OR "Second Step" OR SS-SSTP OR "It's your game" OR DaVIPoP OR (Benzies N2 batchies)))) AND ((MAINSUBJECT.EXACT("Middle schools") OR MAINSUBJECT.EXACT("Junior high schools") OR MAINSUBJECT.EXACT("Grammar schools") OR MAINSUBJECT.EXACT("Primary schools") OR MAINSUBJECT.EXACT("Boarding schools") OR MAINSUBJECT.EXACT("Junior schools") OR MAINSUBJECT.EXACT("Comprehensive schools") OR MAINSUBJECT.EXACT("Elementary schools") OR MAINSUBJECT.EXACT("Secondary schools") OR MAINSUBJECT.EXACT("Preparatory schools") OR MAINSUBJECT.EXACT("Girls' schools") OR MAINSUBJECT.EXACT("High schools") OR MAINSUBJECT.EXACT("Schools") OR MAINSUBJECT.EXACT("Junior secondary schools") OR MAINSUBJECT.EXACT("Classrooms") OR MAINSUBJECT.EXACT("Independent schools")) OR MAINSUBJECT.EXACT("Pupils") OR (ti(school* OR pupil OR pupils OR classroom* OR class-room*) OR ab(school* OR pupil OR pupils OR classroom* OR class-room*)))

**ProQuest Australian Education Index (1977-)**

**Search completed: 23/06/2020**

(MAINSUBJECT.EXACT("Rape") OR MAINSUBJECT.EXACT("Sexual harassment") OR MAINSUBJECT.EXACT("Battered women") OR MAINSUBJECT.EXACT("Homophobia") OR TI(stalking OR stalker* OR rape* OR "intimate partner violence" IPV OR (gender NEAR/3 violen*) OR GBV OR SRGBV OR "violence against women" OR ((domestic OR date OR dating OR relationship* OR partner* OR acquaintance* OR non-stranger* OR nonstranger* OR boyfriend* OR boy-friend* OR girlfriend* OR girl-friend* OR interpersonal OR sexual* OR LGB OR LGBT* OR homosexual* OR lesbian* OR gay OR bisexual* OR queer* OR transgender* OR transsexual) NEAR/3 (abuse* OR abusive OR aggressi* OR assault* OR attack* OR bully* OR coerc* OR cyberbully* OR femicid* OR harass* OR homicid* OR injur* OR manipulate* OR murder* OR rape* OR threaten* OR violen* OR victimi?ation OR revictimi?ation OR re-victimi?ation)) OR ((coerc* OR forced OR unwanted OR nonconsensual OR non-consensual) AND sex*) OR grope OR groping OR groped OR sext OR sexts OR sexting OR homophobi* OR transphobi* OR biphobi* OR homonegativ* OR ("long live love" OR "green dot" OR greendot OR "project respect" OR "Media Aware" OR mediaaware OR TakeCARE OR "Fourth R" OR "Safe Dates" OR "Shifting boundaries" OR "Teen choices" OR "mentors in violence prevention" OR "Expect Respect" OR "Second Step" OR SS-SSTP OR "It's your game" OR DaVIPoP OR (Benzies N2 batchies))) OR AB(stalking OR stalker* OR rape* OR IPV OR "intimate partner violence" OR (gender NEAR/3 violen*) OR GBV OR SRGBV OR "violence against women" OR ((domestic OR date OR dating OR relationship* OR partner* OR acquaintance* OR non-stranger* OR nonstranger* OR boyfriend* OR boy-friend* OR girlfriend* OR girl-friend* OR interpersonal OR sexual* OR LGB OR LGBT* OR homosexual* OR lesbian* OR gay OR bisexual* OR queer* OR transgender* OR transsexual*) NEAR/3 (abuse* OR abusive OR aggressi* OR assault* OR attack* OR bully* OR coerc* OR cyberbully* OR femicid* OR harass* OR homicid* OR injur* OR manipulate* OR murder* OR rape* OR threaten* OR violen* OR victimi?ation OR revictimi?ation OR re-victimi?ation)) OR ((coerc* OR forced OR unwanted OR nonconsensual OR non-consensual) AND sex*) OR grope OR groping OR groped OR sext OR sexts OR sexting OR homophobi* OR transphobi* OR biphobi* OR homonegativ* OR ("long live love" OR "green dot" OR greendot OR "project respect" OR "Media Aware" OR mediaaware OR TakeCARE OR "Fourth R" OR "Safe Dates" OR "Shifting boundaries" OR "Teen choices" OR "mentors in violence prevention" OR "Expect Respect" OR "Second Step" OR SS-SSTP OR "It's your game" OR DaVIPoP OR (Benzies N2 batchies)))) AND ((MAINSUBJECT.EXACT("Disadvantaged schools") OR MAINSUBJECT.EXACT("Nursery schools") OR MAINSUBJECT.EXACT("Schools") OR MAINSUBJECT.EXACT("Primary schools") OR MAINSUBJECT.EXACT("Boarding schools") OR MAINSUBJECT.EXACT("Primary secondary schools") OR MAINSUBJECT.EXACT("Rural schools") OR MAINSUBJECT.EXACT("Middle schools") OR MAINSUBJECT.EXACT("Single sex schools") OR MAINSUBJECT.EXACT("Day schools") OR MAINSUBJECT.EXACT("Secondary schools")) OR MAINSUBJECT.EXACT("School health services") OR (MAINSUBJECT.EXACT("Secondary school students") OR MAINSUBJECT.EXACT("Middle school students") OR MAINSUBJECT.EXACT("Students")) OR (AB(school* OR pupil OR pupils OR classroom* OR class-room*) OR TI(school* OR pupil OR pupils OR classroom* OR class-room*)))

**ProQuest Dissertations & Theses**

**Search completed: 23/06/2020**

(AB(school* OR pupil OR pupils OR classroom* OR class-room*) OR TI(school* OR pupil OR pupils OR classroom* OR class-room*)) AND TI(stalking OR stalker* OR rape* OR IPV OR (gender NEAR/3 violen*) OR GBV OR SRGBV OR "violence against women" OR ((domestic OR date OR dating OR relationship* OR partner* OR acquaintance* OR non-stranger* OR nonstranger* OR boyfriend* OR boy-friend* OR girlfriend* OR girl-friend* OR interpersonal OR sexual* OR LGB OR LGBT* OR homosexual* OR lesbian* OR gay OR bisexual* OR queer* OR transgender* OR transsexual) NEAR/3 (abuse* OR abusive OR aggressi* OR assault* OR attack* OR bully* OR coerc* OR cyberbully* OR femicid* OR harass* OR homicid* OR injur* OR manipulate* OR murder* OR rape* OR threaten* OR violen* OR victimi?ation OR revictimi?ation OR re-victimi?ation)) OR ((coerc* OR forced OR unwanted OR nonconsensual OR non-consensual) AND sex*) OR grope OR groping OR groped OR sext OR sexts OR sexting OR homophobi* OR transphobi* OR biphobi* OR homonegativ* OR ("long live love" OR "green dot" OR greendot OR "project respect" OR "Media Aware" OR mediaaware OR TakeCARE OR "Fourth R" OR "Safe Dates" OR "Shifting boundaries" OR "Teen choices" OR "mentors in violence prevention" OR "Expect Respect" OR "Second Step" OR SS-SSTP OR "It's your game" OR DaVIPoP OR (Benzies N2 batchies))

**ProQuest Sociological Abstracts (1952-)**

**Search completed: 24/06/2020)**

((MAINSUBJECT.EXACT("Stalking") OR MAINSUBJECT.EXACT.EXPLODE("Sexual Assault") OR MAINSUBJECT.EXACT("Battered Women") OR MAINSUBJECT.EXACT("Spouse Abuse") OR MAINSUBJECT.EXACT.EXPLODE("Coercion") OR MAINSUBJECT.EXACT("Homophobia")) OR ti((stalking OR stalker* OR rape* OR IPV OR (gender NEAR/3 violen*) OR GBV OR SRGBV OR "violence against women" OR ((domestic OR date OR dating OR relationship* OR partner* OR acquaintance* OR non-stranger* OR nonstranger* OR boyfriend* OR boy-friend* OR girlfriend* OR girl-friend* OR interpersonal OR sexual* OR LGB OR LGBT* OR homosexual* OR lesbian* OR gay OR bisexual* OR queer* OR transgender* OR transsexual) NEAR/3 (abuse* OR abusive OR aggressi* OR assault* OR attack* OR bully* OR coerc* OR cyberbully* OR femicid* OR harass* OR homicid* OR injur* OR manipulate* OR murder* OR rape* OR threaten* OR violen* OR victimi?ation OR revictimi?ation OR re-victimi?ation))) OR ((coerc* OR forced OR unwanted OR nonconsensual OR non-consensual) AND sex*) OR grope OR groping OR groped OR sext OR sexts OR sexting OR homophobi* OR transphobi* OR biphobi* OR homonegativ* OR ("long live love" OR "green dot" OR greendot OR "project respect" OR "Media Aware" OR mediaaware OR TakeCARE OR "Fourth R" OR "Safe Dates" OR "Shifting boundaries" OR "Teen choices" OR "mentors in violence prevention" OR "Expect Respect" OR "Second Step" OR SS-SSTP OR "It's your game" OR DaVIPoP OR (Benzies N2 batchies)))) AND ((MAINSUBJECT.EXACT("Schools") OR MAINSUBJECT.EXACT("Junior High Schools") OR MAINSUBJECT.EXACT("Secondary Schools") OR MAINSUBJECT.EXACT("Elementary Schools") OR MAINSUBJECT.EXACT("High Schools")) OR (ti(school* OR pupil OR pupils OR classroom* OR class-room*) OR ab(school* OR pupil OR pupils OR classroom* OR class-room*)))

**NHS Economic Evaluation Database (NHS EED) (1994-2015)**

**Search completed: 23/06/2020**

1 MeSH DESCRIPTOR Intimate Partner Violence EXPLODE ALL TREES 28

2 MeSH DESCRIPTOR Stalking EXPLODE ALL TREES 0

3 MeSH DESCRIPTOR Rape EXPLODE ALL TREES 5

4 MeSH DESCRIPTOR Sex Offenses EXPLODE ALL TREES 57

5 MeSH DESCRIPTOR Battered Women EXPLODE ALL TREES 9

6 MeSH DESCRIPTOR Spouse Abuse EXPLODE ALL TREES 28

7 MeSH DESCRIPTOR Coercion EXPLODE ALL TREES 2

8 MeSH DESCRIPTOR Domestic Violence EXPLODE ALL TREES 125

9 MeSH DESCRIPTOR Homophobia EXPLODE ALL TREES 0

10 (stalking or stalker*):TI 0

11 (rape*):TI 2

12 (IPV):TI 1

13 (gender NEAR3 violen*):TI 1

14 (GBV):TI 0

15 (SRGBV):TI 0

16 ((domestic NEAR3 (abuse* or abusive or aggressi* or assault* or attack* or bully* or coerc* or cyberbully* or femicid* or harass* or homicid* or injur* or manipulate* or murder* or rape* or threaten* or violen* or victimization or victimisation or revictimization or revictimisation or re-victimization or re-victimisation))):TI 18

17 ("violence against women"):TI 3

18 (((date or dating) NEAR3 (abuse* or abusive or aggressi* or assault* or attack* or bully* or coerc* or cyberbully* or femicid* or harass* or homicid* or injur* or manipulate* or murder* or rape* or threaten* or violen* or victimization or victimisation or revictimization or revictimisation or re-victimization or re-victimisation))):TI 2

19 (((relationship* or partner* or acquaintance* or non-stranger* or nonstranger*) NEAR3 (abuse* or abusive or aggressi* or assault* or attack* or bully* or coerc* or cyberbully* or femicid* or harass* or homicid* or injur* or manipulate* or murder* or rape* or threaten* or violen* or victimization or victimisation or revictimization or revictimisation or re-victimization or re-victimisation))):TI 25

20 (((boyfriend* or boy-friend* or girlfriend* or girl-friend*) NEAR3 (abuse* or abusive or aggressi* or assault* or attack* or bully* or coerc* or cyberbully* or femicid* or harass* or homicid* or injur* or manipulate* or murder* or rape* or threaten* or violen* or victimization or victimisation or revictimization or revictimisation or re-victimization or re-victimisation))):TI 0

21 (((interpersonal) NEAR3 (abuse* or abusive or aggressi* or assault* or attack* or bully* or coerc* or cyberbully* or femicid* or harass* or homicid* or injur* or manipulate* or murder* or rape* or threaten* or violen* or victimization or victimisation or revictimization or revictimisation or re-victimization or re-victimisation))):TI 1

22 (((sexual*) NEAR3 (abuse* or abusive or aggressi* or assault* or attack* or bully* or coerc* or cyberbully* or femicid* or harass* or homicid* or injur* or manipulate* or murder* or rape* or threaten* or violen* or victimization or victimisation or revictimization or revictimisation or re-victimization or re-victimisation))):TI 35

23 (((coerc* or forced or unwanted or nonconsensual or non-consensual) NEAR2 sex*)):TI 0

24 ((grope or groped or groping)):TI 1

25 (sext or sexts or sexting):TI 0

26 ((homophobi* or transphobi* or biphobi* or homonegativ*)):TI 0

27 (((LGB or LGBT* or homosexual* or lesbian* or gay or bisexual* or queer* or transgender* or transsexual) NEAR3 (abuse* or abusive or aggressi* or assault* or attack* or bully* or coerc* or cyberbully* or Femicid* or harass* or homicid* or injur* or manipulate* or murder* or rape* or threaten* or violen* or victimization or victimisation or revictimization or revictimisation or re-victimization or re-victimisation))):TI 0

28 ((greendot or "green dot")):TI 0

29 ("project respect"):TI 0

30 ( ("Media Aware" or mediaaware)):TI 0

31 (TakeCARE):TI 0

32 ("Fourth R"):TI 0

33 ("Safe Dates"):TI 0

34 ("Shifting boundaries"):TI 0

35 ("Teen choices"):TI 0

36 ("good schools toolkit"):TI 0

37 ("mentors in violence prevention"):TI 0

38 ("expect respect"):TI 0

39 ("second step"):TI 1

40 (SS-SSTP):TI 0

41 (it's your game):TI 0

42 (davipop):TI 0

43 (benzies NEAR2 batchies):TI 0

44 ("long live love"):TI 0

45 #1 OR #2 OR #3 OR #4 OR #5 OR #6 OR #7 OR #8 OR #9 OR #10 OR #11 OR #12 OR #13 OR #14 OR #15 OR #16 OR #17 OR #18 OR #19 OR #20 OR #21 OR #22 OR #23 OR #24 OR #25 OR #26 OR #27 OR #28 OR #29 OR #30 OR #31 OR #32 OR #33 OR #34 OR #35 OR #36 OR #37 OR #38 OR #39 OR #40 OR #41 OR #42 OR #43 OR #44 166

46 MeSH DESCRIPTOR Schools EXPLODE ALL TREES 200

47 MeSH DESCRIPTOR School Health Services EXPLODE ALL TREES 169

48 MeSH DESCRIPTOR Students EXPLODE ALL TREES 88

49 MeSH DESCRIPTOR Curriculum EXPLODE ALL TREES 44

50 (school*):TI 344

51 (pupil or pupils):TI 3

52 (classroom* or class-room*):TI 7

53 #46 OR #47 OR #48 OR #49 OR #50 OR #51 OR #52 524

54 #45 AND #53

**EPPI TRoPHI (Trial Register Promoting Health Interventions)**

**Search completed: 24/06/2020**

1 Freetext (All but Authors): "dating violence" 13

2 Freetext (All but Authors): "relationship violence" 3

3 Freetext (All but Authors): "intimate partner violence" 72

4 Freetext (All but Authors): "gender based violence" 3

5 Freetext (All but Authors): stalking 0

6 Freetext (All but Authors): stalker 0

7 Freetext (All but Authors): rape 8

8 Freetext (All but Authors): "sexual harassment" 4

9 Freetext (All but Authors): battered 1

10 Freetext (All but Authors): coercion 8

11 Freetext (All but Authors): "domestic violence" 19

12 Freetext (All but Authors): IPV 45

13 Freetext (All but Authors): GBV 1

14 Freetext (All but Authors): SRGBV 0

15 Freetext (All but Authors): "violence against women" 8

16 Freetext (All but Authors): homophobia 2

17 Freetext (All but Authors): biphobia 0

18 Freetext (All but Authors): transphobia 0

19 Freetext (All but Authors): "dating abuse" 2

20 Freetext (All but Authors): "relationship abuse" 1

21 Freetext (All but Authors): "partner abuse" 1

22 Freetext (All but Authors): "forced sex" 1

23 Freetext (All but Authors): "forced sexual" 0

24 Freetext (All but Authors): "unwanted sex" 2

25 Freetext (All but Authors): nonconsensual 1

26 Freetext (All but Authors): groping 0

27 Freetext (All but Authors): sexting 0

28 Freetext (All but Authors): sexts 0

29 Freetext (All but Authors): "long live love" 0

30 Freetext (All but Authors): greendot 0

31 Freetext (All but Authors): "green dot" 0

32 Freetext (All but Authors): "project respect" 4

33 Freetext (All but Authors): "media aware" 2

34 Freetext (All but Authors): takecare 1

35 Freetext (All but Authors): "fourth r" 1

36 Freetext (All but Authors): "safe dates" 2

37 Freetext (All but Authors): "shifting boundaries" 2

38 Freetext (All but Authors): "teen choices" 1

39 Freetext (All but Authors): "good schools toolkit" 0

40 Freetext (All but Authors): "good schools toolkit" 0

41 Freetext (All but Authors): "mentors in violence prevention" 0

42 Freetext (All but Authors): "expect respect" 0

43 Freetext (All but Authors): "second step" 5

44 Freetext (All but Authors): SS-SSTP 1

45 Freetext (All but Authors): "it's your game" 5

46 Freetext (All but Authors): davipop 0

47 Freetext (All but Authors): "benzies and batchies" 0

48 1 OR 2 OR 3 OR 4 OR 5 OR 6 OR 7 OR 8 OR 9 OR 10 OR 11 OR 12 OR 13 OR 14 OR 15 OR 16 OR 17 OR 18 OR 19 OR 20 OR 21 OR 22 OR 23 OR 24 OR 25 OR 26 OR 27 OR 28 OR 29 OR 30 OR 31 OR 32 OR 33 OR 34 OR 35 OR 36 OR 37 OR 38 OR 39 OR 40 OR 41 OR 42 OR 44 OR 45 OR 46 OR 47 143

49 Freetext (All but Authors): school 2669

50 Freetext (All but Authors): schools 1829

51 Freetext (All but Authors): pupil 24

52 Freetext (All but Authors): pupils 152

53 Freetext (All but Authors): classroom 384

54 Freetext (All but Authors): classrooms 105

55 Freetext (All but Authors): class-room 3

56 Freetext (All but Authors): class-rooms 0

57 49 OR 50 OR 51 OR 52 OR 53 OR 54 OR 55 OR 56 3080

58 48 AND 57 34

**Web of Science Social Science Citation Index (SSCI) (1956-)**

**Search completed: 23/06/2020**

#21 #20 AND #19

#20 TOPIC: (school* or pupil or pupils or classroom* or class-room*)

#19 #18 OR #17 OR #16 OR #15 OR #14 OR #13 OR #12 OR #11 OR #10 OR #9 OR #8 OR #7 OR #6 OR #5 OR #4 OR #3 OR #2 OR #1

#18 TOPIC: ("long live love" or "green dot" or greendot or "project respect" or "Media Aware" or mediaaware or TakeCARE or "Fourth R" or "Safe Dates" or "Shifting boundaries" or "Teen choices" or "mentors in violence prevention" or "Expect Respect" or "Second Step" or SS-SSTP or "It's your game" or DaVIPoP or (Benzies N2 batchies) )

#17 TI=(((LGB or LGBT* or homosexual* or lesbian* or gay or bisexual* or queer* or transgender* or transsexual) NEAR/3 (abuse* or abusive or aggressi* or assault* or attack* or bully* or coerc* or cyberbully* or femicid* or harass* or homicid* or injur* or manipulate* or murder* or rape* or threaten* or violen* or victimi?ation or revictimi?ation or re-victimi?ation) ))

#16 TI=(homophobi* or transphobi* or biphobi* or homonegativ*)

#15 TI=((grope or groped or groping or sext or sexts or sexting))

#14 TI=(((coerc* or forced or unwanted or nonconsensual or non-consensual) NEAR/2 sex*))

#13 TI=((sexual* NEAR/3 (abusive or aggressi* or assault* or attack* or bully* or coerc* or cyberbully* or femicid* or harass* or homicid* or injur* or manipulate* or murder* or rape* or threaten* or violen* or victimi?ation or revictimi?ation or re-victimi?ation) ))

#12 TI=((interpersonal NEAR/3 (abuse* or abusive or aggressi* or assault* or attack* or bully* or coerc* or cyberbully* or femicid* or harass* or homicid* or injur* or manipulate* or murder* or rape* or threaten* or violen* or victimi?ation or revictimi?ation or re-victimi?ation) ))

#11 TI=(((boyfriend* or boy-friend* or girlfriend* or girl-friend*) NEAR/3 (abuse* or abusive or aggressi* or assault* or attack* or bully* or coerc* or cyberbully* or femicid* or harass* or homicid* or injur* or manipulate* or murder* or rape* or threaten* or violen* or victimi?ation or revictimi?ation or re-victimi?ation) ))

#10 TI=(((relationship* or partner* or acquaintance* or non-stranger* or nonstranger*) NEAR/3 (abuse* or abusive or aggressi* or assault* or attack* or bully* or coerc* or cyberbully* or Femicid* or harass* or homicid* or injur* or manipulate* or murder* or rape* or threaten* or violen* or victimi?ation or revictimi?ation or re-victimi?ation) ))

#9 TI=(((date or dating) NEAR/3 (abuse* or abusive or aggressi* or assault* or attack* or bully* or coerc* or cyberbully* or femicid* or harass* or homicid* or injur* or manipulate* or murder* or rape* or threaten* or violen* or victimi?ation or revictimi?ation or re-victimi?ation) ))

#8 TI=("violence against women")

#7 TI=((domestic NEAR/3 (abuse* or abusive or aggressi* or assault* or attack* or bully* or coerc* or cyberbully* or femicid* or harass* or homicid* or injur* or manipulate* or murder* or rape* or threaten* or violen* or victimi?ation or revictimi?ation or re-victimi?ation) ))

#6 TI=(SRGBV)

#5 TI=(GBV)

#4 TI=((gender NEAR/3 violen*))

#3 TI=(IPV)

#2 TI=(rape*)

#1 TI=(stalking or stalker*)

**Full search terms and strategies: 2021 search update**

**Ovid MEDLINE (R) ALL <1946 to May 28, 2021>**

Search completed: 01/06/2021

1 exp Intimate Partner Violence/ 10281

2 Gender-Based Violence/ 295

3 Stalking/ 220

4 Rape/ 6396

5 Sex Offenses/ 9443

6 Spouse abuse/ 7444

7 Coercion/ 4743

8 Domestic violence/ 6695

9 Homophobia/ 585

10 rape*.ti. 5089

11 (rape adj myth*).ab. 311

12 (rape adj1 acquaintance).ab. 82

13 (date adj rape).ab. 189

14 "intimate partner violence".ti,ab. 8292

15 "intimate partner abuse".ti,ab. 224

16 (gender* adj3 violen*).ti,ab. 1897

17 (domestic adj3 (abuse* or abusive or aggressi* or assault* or attack* or bully* or coerc* or cyberbully* or femicid* or harass* or homicid* or injur* or manipulate* or murder* or rape* or threaten* or violen* or victimi?ation or revictimi?ation or re-victimi?ation)).ti,ab. 7451

18 "violence against women".ti,ab. 2626

19 (dating adj3 (abuse* or abusive or aggressi* or assault* or attack* or bully* or coerc* or cyberbully* or femicid* or harass* or homicid* or injur* or manipulate* or murder* or rape* or threaten* or violen* or victimi?ation or revictimi?ation or re-victimi?ation)).ti,ab. 1392

20 (relationship* adj (abuse* or abusive or aggressi* or assault* or attack* or bully* or coerc* or cyberbully* or femicid* or harass* or homicid* or injur* or manipulate* or murder* or rape* or threaten* or violen* or victimi?ation or revictimi?ation or re-victimi?ation)).ti,ab. 386

21 ((boyfriend* or boy-friend* or girlfriend* or girl-friend*) adj3 (abuse* or abusive or aggressi* or assault* or attack* or bully* or coerc* or cyberbully* or femicid* or harass* or homicid* or injur* or manipulate* or murder* or rape* or threaten* or violen* or victimi?ation or revictimi?ation or re-victimi?ation)).ti,ab. 48

22 (sexual* adj1 (abusive or aggressi* or assault* or attack* or bully* or coerc* or cyberbully* or femicid* or harass* or homicid* or injur* or manipulate* or murder* or rape* or threaten* or violen* or victimi?ation or revictimi?ation or re-victimi?ation)).ti,ab. 13290

23 ((coerc* or forced or unwanted or nonconsensual or non-consensual) adj1 sex*).ti,ab. 2050

24 (homophobi* or transphobi* or biphobi* or homonegativ*).ti,ab. 1814

25 ((LGB or LGBT* or homosexual* or lesbian* or gay or bisexual* or queer* or transgender* or transsexual*) adj3 (abuse* or abusive or aggressi* or assault* or attack* or bully* or coerc* or cyberbully* or femicid* or harass* or homicid* or injur* or manipulate* or murder* or rape* or threaten* or violen* or victimi?ation or revictimi?ation or re-victimi?ation)).ti,ab. 742

26 "5 W's of bullying intervention".tw. 0

27 "alberta healthy youth relationships".tw. 1

28 "athletes as leaders".tw. 2

29 "architects of their own brain".tw. 1

30 (Benzies adj2 Batchies).ti,ab. 1

31 ("break the cycle" and (end* adj2 violence)).tw. 0

32 ("bringing in the bystander" and "high school").tw. 4

33 BITB-HSC.tw. 3

34 "building relationships in greater harmony together".tw. 0

35 ("challenging violence" adj2 "changing lives").tw. 0

36 "change up project".tw. 0

37 "chesterfield relate".tw. 0

38 "connect with respect".tw. 0

39 (Connections and "dating and emotions curriculum").tw. 0

40 "coaching boys into men".tw. 10

41 "dat-e adolescence".tw. 3

42 "dating matters".tw. 13

43 "Expect Respect".tw. 8

44 ("familias en nuestra escuela" or "families in our school").tw. 1

45 ("filles et garcons" adj2 "en route pour l'egalite").tw. 0

46 "Fourth R".ti,ab. 25

47 "gender equity movement in schools".tw. 0

48 (go adj girls* adj initiative).tw. 0

49 "good schools toolkit".ti,ab. 2

50 "green acres high".tw. 0

51 (greendot or "green dot").ti,ab. 36

52 (healthy adj relationships).tw. 298

53 "human relationships education".tw. 0

54 IMPower.tw. 23

55 "Juntos opuestos a la violence entre novios".tw. 1

56 "katie brown educational program".tw. 1

57 "La máscara del amor".tw. 0

58 ("long live love" or "long live love+").tw. 4

59 "let us protect our future".tw. 4

60 lights4violence.tw. 5

61 "love doesn't hurt".tw. 0

62 "love hurts".tw. 14

63 "mask of love".tw. 0

64 ("Media Aware" or mediaaware).ti,ab. 6

65 "mentors in violence prevention".ti,ab. 5

66 "my voice, my choice".tw. 1

67 "papa reto".tw. 0

68 "power up, speak out!".tw. 0

69 (prepare and "promoting sexual and reproductive health" and "eastern africa").tw. 2

70 "eliminating coercion and pressure in adolescent relationships".tw. 1

71 "previo program".tw. 0

72 "project connect".tw. 19

73 "project pride".tw. 6

74 "project respect".tw. 29

75 R4Respect.ti,ab. 0

76 "reduction of stigma in schools".tw. 0

77 "relaciones romanticas constructivas".tw. 0

78 "relationships without fear".tw. 0

79 "respectful relationships".tw. 78

80 "Safe Dates".ti,ab. 26

81 "safe schools".ti,ab. 30

82 "school health center healthy adolescent relationship program".tw. 0

83 "Shifting boundaries".ti,ab. 52

84 ("Second Step" and (program* or intervention*)).ti,ab. 772

85 "skillz street".tw. 2

86 skhokho.tw. 3

87 SS-SSTP.ti,ab. 1

88 "start strong initiative".tw. 1

89 ("stay in love" or "stay in love+").tw. 1

90 TakeCARE.ti,ab. 15

91 "Teen choices".ti,ab. 4

92 "trust education project".tw. 0

93 DaVIPoP.ti,ab. 0

94 "young men initiative".tw. 1

95 ("zero tolerance" and respect and project).tw. 0

96 or/1-95 55259

97 Schools/ 41336

98 exp School Health Services/ 23545

99 school*.ti,ab,jw. 309262

100 or/97-99 320025

101 96 and 100 3192

102 (intervention* or program* or prevent* or instruct* or strateg* or curricul* or project* or initiative*).tw. 4353306

103 101 and 102 1932 [These results were deduplicated against the original search results set from June 2020. 268 records after deduplication]

### **Ovid Embase <1974 to 2021 May 28>**

**Search completed: 01/06/2021**

1 exp partner violence/ [NT marital rape] 13810

2 dating violence/ 666

3 gender based violence/ 1014

4 exp sexual assault/ [NT drug-facilitated sexual assault, rape, acquaintance rape, attempted rape, sexual abuse, sexual harassment, sexual bullying] 36908

5 exp stalking/ [NT cyberstalking] 722

6 sexual violence/ 2968

7 sexual coercion/ 416

8 sexual exploitation/ 496

9 sexual crime/ 11678

10 battered woman/ 3309

11 domestic violence/ 9601

12 homophobia/ 1219

13 (stalking or stalker*).ti,ab. 1009

14 (rape adj myth*).ab. 352

15 (rape adj1 acquaintance).ab. 84

16 (date adj rape).ab. 245

17 (gender* adj3 violen*).ti,ab. 2153

18 "intimate partner violence".ti,ab. 8943

19 "intimate partner abuse".ti,ab. 243

20 GBV.ti,ab. 1373

21 (domestic adj3 (abuse* or abusive or aggressi* or assault* or attack* or bully* or coerc* or cyberbully* or femicid* or harass* or homicid* or injur* or manipulate* or murder* or rape* or threaten* or violen* or victimi?ation or revictimi?ation or re-victimi?ation)).ti,ab. 8987

22 "violence against women".ti,ab. 2777

23 (dating adj3 (abuse* or abusive or aggressi* or assault* or attack* or bully* or coerc* or cyberbully* or femicid* or harass* or homicid* or injur* or manipulate* or murder* or rape* or threaten* or violen* or victimi?ation or revictimi?ation or re-victimi?ation)).ti,ab. 1584

24 (relationship* adj (abuse* or abusive or aggressi* or assault* or attack* or bully* or coerc* or cyberbully* or femicid* or harass* or homicid* or injur* or manipulate* or murder* or rape* or threaten* or violen* or victimi?ation or revictimi?ation or re-victimi?ation)).ti,ab. 445

25 ((boyfriend* or boy-friend* or girlfriend* or girl-friend*) adj3 (abuse* or abusive or aggressi* or assault* or attack* or bully* or coerc* or cyberbully* or femicid* or harass* or homicid* or injur* or manipulate* or murder* or rape* or threaten* or violen* or victimi?ation or revictimi?ation or re-victimi?ation)).ti,ab. 59

26 (sexual* adj3 (abusive or aggressi* or assault* or attack* or bully* or coerc* or cyberbully* or femicid* or harass* or homicid* or injur* or manipulate* or murder* or rape* or threaten* or violen* or victimi?ation or revictimi?ation or re-victimi?ation)).ti,ab. 20484

27 ((coerc* or forced or unwanted or nonconsensual or non-consensual) adj1 sex*).ti,ab. 2495

28 (homophobi* or transphobi* or biphobi* or homonegativ*).ti,ab. 2022

29 ((LGB or LGBT* or homosexual* or lesbian* or gay or bisexual* or queer* or transgender* or transsexual) adj3 (abuse* or abusive or aggressi* or assault* or attack* or bully* or coerc* or cyberbully* or femicid* or harass* or homicid* or injur* or manipulate* or murder* or rape* or threaten* or violen* or victimi?ation or revictimi?ation or re-victimi?ation)).ti,ab. 846

30 "5 W's of bullying intervention".tw. 0

31 "alberta healthy youth relationships".tw. 1

32 "athletes as leaders".tw. 2

33 "architects of their own brain".tw. 0

34 (Benzies adj2 Batchies).ti,ab. 1

35 ("break the cycle" and (end* adj2 violence)).tw. 0

36 ("bringing in the bystander" and "high school").tw. 4

37 BITB-HSC.tw. 3

38 "building relationships in greater harmony together".tw. 0

39 ("challenging violence" adj2 "changing lives").tw. 0

40 "change up project".tw. 0

41 "chesterfield relate".tw. 0

42 "connect with respect".tw. 0

43 (Connections and "dating and emotions curriculum").tw. 0

44 "coaching boys into men".tw. 14

45 "dat-e adolescence".tw. 2

46 "dating matters".tw. 11

47 "Expect Respect".tw. 10

48 ("familias en nuestra escuela" or "families in our school").tw. 1

49 ("filles et garcons" adj2 "en route pour l'egalite").tw. 0

50 "Fourth R".ti,ab. 37

51 "gender equity movement in schools".tw. 0

52 (go adj girls* adj initiative).tw. 0

53 "good schools toolkit".ti,ab. 2

54 "green acres high".tw. 0

55 (greendot or "green dot").ti,ab. 54

56 (healthy adj relationships).tw. 393

57 "human relationships education".tw. 0

58 IMPower.tw. 73

59 "Juntos opuestos a la violence entre novios".tw. 1

60 "katie brown educational program".tw. 1

61 "La máscara del amor".tw. 0

62 ("long live love" or "long live love+").tw. 5

63 "let us protect our future".tw. 4

64 lights4violence.tw. 5

65 "love doesn't hurt".tw. 0

66 "love hurts".tw. 15

67 "mask of love".tw. 0

68 ("Media Aware" or mediaaware).ti,ab. 6

69 "mentors in violence prevention".ti,ab. 5

70 "my voice, my choice".tw. 3

71 "papa reto".tw. 0

72 "power up, speak out!".tw. 0

73 (prepare and "promoting sexual and reproductive health" and "eastern africa").tw. 2

74 "eliminating coercion and pressure in adolescent relationships".tw. 3

75 "previo program".tw. 0

76 "project connect".tw. 45

77 "project pride".tw. 8

78 "project respect".tw. 34

79 R4Respect.ti,ab. 0

80 "reduction of stigma in schools".tw. 0

81 "relaciones romanticas constructivas".tw. 0

82 "relationships without fear".tw. 0

83 "respectful relationships".tw. 96

84 "Safe Dates".ti,ab. 25

85 "safe schools".ti,ab. 37

86 "school health center healthy adolescent relationship program".tw. 0

87 "Shifting boundaries".ti,ab. 49

88 ("Second Step" and (program* or intervention*)).ti,ab. 1214

89 "skillz street".tw. 1

90 skhokho.tw. 3

91 SS-SSTP.ti,ab. 1

92 "start strong initiative".tw. 2

93 ("stay in love" or "stay in love+").tw. 1

94 TakeCARE.ti,ab. 17

95 "Teen choices".ti,ab. 2

96 "trust education project".tw. 0

97 DaVIPoP.ti,ab. 0

98 "young men initiative".tw. 1

99 ("zero tolerance" and respect and project).tw. 1

100 or/1-99 83840

101 school/ or high school/ or kindergarten/ or middle school/ or nursery school/ or primary school/ 98560

102 school health service/ 13851

103 school health nursing/ 5449

104 school*.ti,ab,jx. 379658

105 or/101-104 396879

106 (intervention* or program* or prevent* or instruct* or strateg* or curricul* or project* or initiative*).tw. 5714007

107 100 and 105 and 106 3153 [deduplicated against previous result set – 449 records]

### **Ovid APA PsycINFO <1806 to May Week 4 2021>**

**Search completed: 01/06/2021**

1 intimate partner violence/ 12392

2 stalking/ 863

3 exp rape/ 6151

4 sex offenses/ 11012

5 sexual harassment/ 2716

6 battered females/ 3227

7 coercion/ 2499

8 domestic violence/ 11955

9 sexting/ 312

10 (stalking or stalker*).ti,ab. 1435

11 rape*.ti. 3530

12 (rape adj myth*).ab. 879

13 (rape adj1 acquaintance).ab. 283

14 (date adj rape).ab. 357

15 "intimate partner violence".ti,ab. 8855

16 "intimate partner abuse".ti,ab. 349

17 (gender* adj3 violen*).ti,ab. 2482

18 (domestic adj3 (abuse* or abusive or aggressi* or assault* or attack* or bully* or coerc* or cyberbully* or femicid* or harass* or homicid* or injur* or manipulat* or murder* or rape* or threaten* or violen* or victimi?ation or revictimi?ation or re-victimi?ation)).ti,ab. 10984

19 "violence against women".ti,ab. 3123

20 (dating adj3 (abuse* or abusive or aggressi* or assault* or attack* or bully* or coerc* or cyberbully* or femicid* or harass* or homicid* or injur* or manipulat* or murder* or rape* or threaten* or violen* or victimi?ation or revictimi?ation or re-victimi?ation)).ti,ab. 2209

21 (relationship* adj (abuse* or abusive or aggressi* or assault* or attack* or bully* or coerc* or cyberbully* or femicid* or harass* or homicid* or injur* or manipulat* or murder* or rape* or threaten* or violen* or victimi?ation or revictimi?ation or re-victimi?ation)).ti,ab. 775

22 ((boyfriend* or boy-friend* or girlfriend* or girl-friend*) adj3 (abuse* or abusive or aggressi* or assault* or attack* or bully* or coerc* or cyberbully* or femicid* or harass* or homicid* or injur* or manipulat* or murder* or rape* or threaten* or violen* or victimi?ation or revictimi?ation or re-victimi?ation)).ti,ab. 81

23 (sexual* adj1 (abusive or aggressi* or assault* or attack* or bully* or coerc* or cyberbully* or femicid* or harass* or homicid* or injur* or manipulat* or murder* or rape* or threaten* or violen* or victimi?ation or revictimi?ation or re-victimi?ation)).ti,ab. 19354

24 ((coerc* or forced or unwanted or nonconsensual or non-consensual) adj1 sex*).ti,ab. 2842

25 (homophobi* or transphobi* or biphobi* or homonegativ*).ti,ab. 4857

26 ((LGB or LGBT* or homosexual* or lesbian* or gay or bisexual* or queer* or transgender* or transsexual*) adj3 (abuse* or abusive or aggressi* or assault* or attack* or bully* or coerc* or cyberbully* or femicid* or harass* or homicid* or injur* or manipulate* or murder* or rape* or threaten* or violen* or victimi?ation or revictimi?ation or re-victimi?ation)).ti,ab. 1518

27 "5 W's of bullying intervention".tw. 1

28 "alberta healthy youth relationships".tw. 1

29 "athletes as leaders".tw. 7

30 "architects of their own brain".tw. 1

31 (Benzies adj2 Batchies).ti,ab. 0

32 ("break the cycle" and (end* adj2 violence)).tw. 2

33 ("bringing in the bystander" and "high school").tw. 3

34 BITB-HSC.tw. 2

35 "building relationships in greater harmony together".tw. 0

36 ("challenging violence" adj2 "changing lives").tw. 0

37 "change up project".tw. 1

38 "chesterfield relate".tw. 1

39 "coaching boys into men".tw. 11

40 "connect with respect".tw. 0

41 (Connections and "dating and emotions curriculum").tw. 0

42 "dat-e adolescence".tw. 1

43 "dating matters".tw. 12

44 "expect respect".tw. 19

45 ("familias en nuestra escuela" or "families in our school").tw. 1

46 ("filles et garcons" adj2 "en route pour l'egalite").tw. 0

47 "Fourth R".ti,ab. 32

48 "gender equity movement in schools".tw. 0

49 (go adj girls* adj initiative).tw. 0

50 "good schools toolkit".ti,ab. 1

51 "long live love".ti,ab. 5

52 "green acres high".tw. 1

53 (greendot or "green dot").ti,ab. 33

54 (healthy adj relationships).tw. 823

55 "human relationships education".tw. 2

56 IMPower.tw. 4

57 "Juntos opuestos a la violence entre novios".tw. 1

58 "katie brown educational program".tw. 0

59 "La máscara del amor".tw. 0

60 "let us protect our future".tw. 5

61 ("long live love" or "long live love+").tw. 5

62 "love doesn't hurt".tw. 1

63 "love hurts".tw. 22

64 lights4violence.tw. 2

65 "mask of love".tw. 0

66 ("Media Aware" or mediaaware).ti,ab. 9

67 "mentors in violence prevention".ti,ab. 10

68 "my voice, my choice".tw. 1

69 "papa reto".tw. 0

70 "power up, speak out!".tw. 0

71 (prepare and "promoting sexual and reproductive health" and "eastern africa").tw. 0

72 "previo program".tw. 1

73 "project connect".tw. 29

74 "project pride".tw. 12

75 "project respect".tw. 15

76 R4Respect.ti,ab. 0

77 "reduction of stigma in schools".tw. 1

78 "relaciones romanticas constructivas".tw. 0

79 "relationships without fear".tw. 3

80 "respectful relationships".tw. 130

81 TakeCARE.ti,ab. 6

82 "Safe Dates".ti,ab. 30

83 "safe schools".ti,ab. 192

84 "school health center healthy adolescent relationship program".tw. 0

85 "Shifting boundaries".ti,ab. 87

86 "Teen choices".ti,ab. 4

87 "trust education project".tw. 0

88 ("Second Step" and (program* or intervention*)).ti,ab. 300

89 SS-SSTP.ti,ab. 4

90 "It's your game".ti,ab. 9

91 DaVIPoP.ti,ab. 1

92 "young men initiative".tw. 1

93 ("zero tolerance" and respect and project).tw. 3

94 or/1-93 67794

95 exp schools/ 72222

96 high school graduates/ or high school students/ or junior high school students/ or kindergarten students/ or middle school students/ or preschool students/ 65741

97 school*.ti,ab,jn. 402969

98 or/95-97 442927

99 (intervention* or program* or prevent* or instruct* or strateg* or curricul* or project* or initiative*).tw. 1365288

100 school based intervention/ 19849

101 99 or 100 1366583

102 94 and 98 and 101 3103 [deduplicated against previous result set – 771 records]

### **CENTRAL via the Cochrane Library**

**Search completed: 01/06/2021**

#1 MeSH descriptor: [Intimate Partner Violence] explode all trees

#2 MeSH descriptor: [Gender-Based Violence] explode all trees

#3 MeSH descriptor: [Stalking] explode all trees

#4 MeSH descriptor: [Rape] explode all trees

#5 MeSH descriptor: [Sex Offenses] explode all trees

#6 MeSH descriptor: [Battered Women] explode all trees

#7 MeSH descriptor: [Spouse Abuse] explode all trees

#8 MeSH descriptor: [Coercion] explode all trees

#9 MeSH descriptor: [Domestic Violence] explode all trees

#10 MeSH descriptor: [Homophobia] explode all trees

#11 rape*:ti

#12 ("rape myth" or "acquaintance rape" or "date rape"):ab,kw

#13 "intimate partner violence":ti,ab,kw

#14 IPV:ti,ab,kw

#15 (gender* near/3 violen*):ti,ab,kw

#16 GBV:ti,ab,kw

#17 SRGBV:ti,ab,kw

#18 "violence against women":ti,ab,kw

#19 (domestic near/3 (abuse* or abusive or aggressi* or assault* or attack* or bully* or coerc* or cyberbully* or femicid* or harass* or homicid* or injur* or manipulat* or murder* or rape* or threaten* or violen* or victimisation or victimization or revictimisation or revictimization or re-victimisation or re-victimization)):ti,ab,kw

#20 ((dating) near/3 (abuse* or abusive or aggressi* or assault* or attack* or bully* or coerc* or cyberbully* or femicid* or harass* or homicid* or injur* or manipulat* or murder* or rape* or threaten* or violen* or victimisation or victimization or revictimisation or revictimization or re-victimisation or re-victimization)):ti,ab,kw

#21 ((relationship*) near/1 (abuse* or abusive or aggressi* or assault* or attack* or bully* or coerc* or cyberbully* or femicid* or harass* or homicid* or injur* or manipulat* or murder* or rape* or threaten* or violen* or victimisation or victimization or revictimisation or revictimization or re-victimisation or re-victimization)):ti,ab,kw

#22 ((boyfriend* or boy-friend* or girlfriend* or girl-friend*) near/3 (abuse* or abusive or aggressi* or assault* or attack* or bully* or coerc* or cyberbully* or femicid* or harass* or homicid* or injur* or manipulat* or murder* or rape* or threaten* or violen* or victimisation or victimization or revictimisation or revictimization or re-victimisation or re-victimization)):ti,ab,kw

#23 (sexual* near/1 (aggressi* or assault* or attack or coerc* or cyberbully* or femicid* or harass* or homicid* or injur* or manipulat* or murder* or rape* or threaten* or violen* or victimization or revictimisation or revictimization or re-victimisation or re-victimization)):ti,ab,kw

#24 ((coerc* or forced or unwanted or nonconsensual or non-consensual) near/1 sex*):ti,ab,kw

#25 (homophobi* or transphobi* or biphobi* or homonegativ*):ti,ab,kw

#26 ((LGB or LGBT* or homosexual* or lesbian* or gay or bisexual* or queer* or transgender* or transsexual) near/3 (abuse* or abusive or aggressi* or assault* or attack* or bully* or coerc* or cyberbully* or femicid* or harass* or homicid* or injur* or manipulate* or murder* or rape* or threaten* or violen* or victimisation or victimization or revictimisation or revictimization or re-victimisation or re-victimization)):ti,ab,kw

#27 ("5 W's of bullying intervention" OR "alberta healthy youth relationships" OR "athletes as leaders" OR "architects of their own brain" OR (Benzies NEAR/2 Batchies) OR ("break the cycle" and (end* NEAR/2 violence) ) OR ("bringing in the bystander" and "high school") OR BITB-HSC OR "building relationships in greater harmony together" OR ("challenging violence" NEAR/2 "changing lives") OR "change up project" OR "chesterfield relate" OR "connect with respect" OR (Connections and "dating and emotions curriculum") OR "coaching boys into men" OR "dat-e adolescence" OR "dating matters" OR "Expect Respect" OR ("familias en nuestra escuela" or "families in our school") OR ("filles et garcons" NEAR/2 "en route pour l'egalite") OR "Fourth R" OR "gender equity movement in schools" OR (“go girls initiative”) OR "good schools toolkit" OR "green acres high" OR (greendot or "green dot") OR ("healthy relationships") OR "human relationships education" OR IMPower OR "Juntos opuestos a la violence entre novios" OR "katie brown educational program" OR "La máscara del amor" OR ("long live love" or "long live love+") OR "let us protect our future" OR lights4violence OR "love doesn't hurt" OR "love hurts" OR "mask of love" OR ("Media Aware" or mediaaware) OR "mentors in violence prevention" OR "my voice, my choice" OR “papa reto" OR “power up, speak out!" OR (prepare and "promoting sexual and reproductive health" and "eastern africa") OR "eliminating coercion and pressure in adolescent relationships" OR "previo program" OR "project connect" OR "project pride" OR "project respect" OR R4Respect OR "reduction of stigma in schools" OR "relaciones romanticas constructivas" OR "relationships without fear" OR "respectful relationships" OR "Safe Dates" OR "safe schools" OR "school health center healthy adolescent relationship program" OR "Shifting boundaries" OR ("Second Step" and (program* or intervention*) ) OR "skillz street" OR skhokho OR SS-SSTP OR "start strong initiative" OR ("stay in love" or "stay in love+") OR TakeCARE OR "Teen choices" OR "trust education project" OR DaVIPoP OR "young men initiative" OR ("zero tolerance" and respect and project)):ti,ab

#28 {OR #1-#27}

#29 MeSH descriptor: [Schools] explode all trees

#30 MeSH descriptor: [School Health Services] explode all trees

#31 (school*):ti,ab,kw

#32 {OR #29-#31}

#33 #28 AND #32 [464]

### Web of Science Social Citation Index

Search completed: 1/06/2021

#20 (1,568) #19 AND #18 Indexes=SSCI, CPCI-SSH Timespan=All years

#19 (1,721,313) TI=(intervention* or program* or prevent* or instruct* or strateg* or curricul* or project* or initiative*) OR AB=(intervention* or program* or prevent* or instruct* or strateg* or curricul* or project* or initiative*) Indexes=SSCI, CPCI-SSH Timespan=All years

#18 (2,694) #17 AND #16 Indexes=SSCI, CPCI-SSH Timespan=All years

#17 (398,432) TS=(school*) or SO=(school*) Indexes=SSCI, CPCI-SSH Timespan=All years

#16 (38,031) #15 OR #14 OR #13 OR #12 OR #11 OR #10 OR #9 OR #8 OR #7 OR #6 OR #5 OR #4 OR #3 OR #2 OR #1 Indexes=SSCI, CPCI-SSH Timespan=All years

#15 (1,738) TS=("5 W's of bullying intervention" OR "alberta healthy youth relationships" OR "athletes as leaders" OR "architects of their own brain" OR (Benzies NEAR/2 Batchies) OR ("break the cycle" and (end* NEAR/2 violence) ) OR ("bringing in the bystander" and "high school") OR BITB-HSC OR "building relationships in greater harmony together" OR ("challenging violence" NEAR/2 "changing lives") OR "change up project" OR "chesterfield relate" OR "connect with respect" OR (Connections and "dating and emotions curriculum") OR "coaching boys into men" OR "dat-e adolescence" OR "dating matters" OR "Expect Respect" OR ("familias en nuestra escuela" or "families in our school") OR ("filles et garcons" NEAR/2 "en route pour l'egalite") OR "Fourth R" OR "gender equity movement in schools" OR (“go girls initiative”) OR "good schools toolkit" OR "green acres high" OR (greendot or "green dot") OR ("healthy relationships") OR "human relationships education" OR IMPower OR "Juntos opuestos a la violence entre novios" OR "katie brown educational program" OR "La máscara del amor" OR ("long live love" or "long live love+") OR "let us protect our future" OR lights4violence OR "love doesn't hurt" OR "love hurts" OR "mask of love" OR ("Media Aware" or mediaaware) OR "mentors in violence prevention" OR "my voice, my choice" OR “papa reto" OR “power up, speak out!" OR (prepare and "promoting sexual and reproductive health" and "eastern africa") OR "eliminating coercion and pressure in adolescent relationships" OR "previo program" OR "project connect" OR "project pride" OR "project respect" OR R4Respect OR "reduction of stigma in schools" OR "relaciones romanticas constructivas" OR "relationships without fear" OR "respectful relationships" OR "Safe Dates" OR "safe schools" OR "school health center healthy adolescent relationship program" OR "Shifting boundaries" OR ("Second Step" and (program* or intervention*) ) OR "skillz street" OR skhokho OR SS-SSTP OR "start strong initiative" OR ("stay in love" or "stay in love+") OR TakeCARE OR "Teen choices" OR "trust education project" OR DaVIPoP OR "young men initiative" OR ("zero tolerance" and respect and project) ) Indexes=SSCI, CPCI-SSH Timespan=All years

#14 (485) TI=(((LGB or LGBT* or homosexual* or lesbian* or gay or bisexual* or queer* or transgender* or transsexual) NEAR/3 (abuse* or abusive or aggressi* or assault* or attack* or bully* or coerc* or cyberbully* or femicid* or harass* or homicid* or injur* or manipulate* or murder* or rape* or threaten* or violen* or victimi?ation or revictimi?ation or re-victimi?ation) )) Indexes=SSCI, CPCI-SSH Timespan=All years

#13 (1,083) TI=(homophobi* or transphobi* or biphobi* or homonegativ*) Indexes=SSCI, CPCI-SSH Timespan=All years

#12 (779) TI=(((coerc* or forced or unwanted or nonconsensual or non-consensual) NEAR/1 sex*)) Indexes=SSCI, CPCI-SSH Timespan=All years

#11 (9,222) TI=((sexual* NEAR/1 (abusive or aggressi* or assault* or attack* or bully* or coerc* or cyberbully* or femicid* or harass* or homicid* or injur* or manipulate* or murder* or rape* or threaten* or violen* or victimi?ation or revictimi?ation or re-victimi?ation) )) Indexes=SSCI, CPCI-SSH Timespan=All years

#10 (8) TI=(((boyfriend* or boy-friend* or girlfriend* or girl-friend*) NEAR/3 (abuse* or abusive or aggressi* or assault* or attack* or bully* or coerc* or cyberbully* or femicid* or harass* or homicid* or injur* or manipulate* or murder* or rape* or threaten* or violen* or victimi?ation or revictimi?ation or re-victimi?ation) )) Indexes=SSCI, CPCI-SSH Timespan=All years

#9 (1,107) TI=(((relationship*) NEAR/1 (abuse* or abusive or aggressi* or assault* or attack* or bully* or coerc* or cyberbully* or femicid* or harass* or homicid* or injur* or manipulate* or murder* or rape* or threaten* or violen* or victimi?ation or revictimi?ation or re-victimi?ation) )) Indexes=SSCI, CPCI-SSH Timespan=All years

#8 (1,404) TI=(((dating) NEAR/2 (abuse* or abusive or aggressi* or assault* or attack* or bully* or coerc* or cyberbully* or femicid* or harass* or homicid* or injur* or manipulate* or murder* or rape* or threaten* or violen* or victimi?ation or revictimi?ation or re-victimi?ation) )) Indexes=SSCI, CPCI-SSH Timespan=All years

#7 (1,644) TI=("violence against women") Indexes=SSCI, CPCI-SSH Timespan=All years

#6 (4,649) TI=((domestic NEAR/1 (abuse* or abusive or aggressi* or assault* or attack* or bully* or coerc* or cyberbully* or femicid* or harass* or homicid* or injur* or manipulate* or murder* or rape* or threaten* or violen* or victimi?ation or revictimi?ation or re-victimi?ation) )) Indexes=SSCI, CPCI-SSH Timespan=All years

#5 (4) TS=(SRGBV) Indexes=SSCI, CPCI-SSH Timespan=All years

#4 (237) TS=(GBV) Indexes=SSCI, CPCI-SSH Timespan=All years

#3 (1,495) TI=((gender* NEAR/2 violen*)) Indexes=SSCI, CPCI-SSH Timespan=All years

#2 (15,077) TI=("partner violence" or "partner abuse") OR TS=("intimate partner violence" OR "intimate partner abuse") Indexes=SSCI, CPCI-SSH Timespan=All years

#1 (4,868) TI=(rape*) OR AB=(rape myth) OR AB=(acquaintance rape) OR AB=("date rape") Indexes=SSCI, CPCI-SSH Timespan=All years

### **EBSCO CINAHL Complete (1937-2020)**

**Search completed: 1/06/2021**

S103 S101 AND S102 (1562)

S102 TI ( (intervention* or program* or prevent* or instruct* or strateg* or curricul* or project* or initiative*) ) OR AB ( (intervention* or program* or prevent* or instruct* or strateg* or curricul* or project* or initiative*) )

S101 S94 AND S100

S100 S95 OR S96 OR S97 OR S98 OR S99

S99 TI school* OR AB school*

S98 SO school*

S97 (MH "Students, High School") OR (MH "Students, Middle School") OR (MH "Students, Elementary")

S96 (MH "School Health Services+")

S95 (MH "Schools") OR (MH "Schools, Elementary") OR (MH "Schools, Middle") OR (MH "Schools, Nursery") OR (MH "Schools, Secondary")

S94 S1 OR S2 OR S3 OR S4 OR S5 OR S6 OR S7 OR S8 OR S9 OR S10 OR S11 OR S12 OR S13 OR S14 OR S15 OR S16 OR S17 OR S18 OR S19 OR S20 OR S21 OR S22 OR S23 OR S24 OR S25 OR S26 OR S27 OR S28 OR S29 OR S30 OR S31 OR S32 OR S33 OR S34 OR S35 OR S36 OR S37 OR S38 OR S39 OR S40 OR S41 OR S42 OR S43 OR S44 OR S45 OR S46 OR S47 OR S48 OR S49 OR S50 OR S51 OR S52 OR S53 OR S54 OR S55 OR S56 OR S57 OR S58 OR S59 OR S60 OR S61 OR S62 OR S63 OR S64 OR S65 OR S66 OR S67 OR S68 OR S69 OR S70 OR S71 OR S72 OR S73 OR S74 OR S75 OR S76 OR S77 OR S78 OR S79 OR S80 OR S81 OR S82 OR S83 OR S84 OR S85 OR S86 OR S87 OR S88 OR S89 OR S93

S93 TI ( ("zero tolerance" and respect and project) ) OR AB ( ("zero tolerance" and respect and project) )

S92 TI "young men initiative" OR AB "young men initiative"

S91 TI DaVIPoP OR AB DaVIPoP

S90 TI "trust education project" OR AB "trust education project"

S89 TI "Teen choices" OR AB "Teen choices"

S88 TI TakeCARE OR AB TakeCARE

S87 TI ( ("stay in love" or "stay in love+") ) OR AB ( ("stay in love" or "stay in love+") )

S86 TI "start strong initiative" OR AB "start strong initiative"

S85 TI SS-SSTP OR AB SS-SSTP

S84 TI skhokho OR AB skhokho

S83 TI "skillz street" OR AB "skillz street"

S82 TI ( ("Second Step" and (program* or intervention*)) ) OR AB ( ("Second Step" and (program* or intervention*)) )

S81 TI "Shifting boundaries" OR AB "Shifting boundaries"

S80 TI "school health center healthy adolescent relationship program" OR AB "school health center healthy adolescent relationship program"

S79 TI "safe schools" OR AB "safe schools"

S78 TI "Safe Dates" OR AB "Safe Dates"

S77 TI "respectful relationships" OR AB "respectful relationships"

S76 TI "relationships without fear" OR AB "relationships without fear"

S75 TI "relaciones romanticas constructivas" OR AB "relaciones romanticas constructivas"

S74 TI "reduction of stigma in schools" OR AB "reduction of stigma in schools"

S73 TI R4Respect OR AB R4Respect

S72 TI "project respect" OR AB "project respect"

S71 TI "project pride" OR AB "project pride"

S70 TI "project connect" OR AB "project connect"

S69 TI "previo program" OR AB "previo program"

S68 TI ( "eliminating coercion and pressure in adolescent relationships" ) OR AB ( "eliminating coercion and pressure in adolescent relationships" )

S67 TI ( (prepare and "promoting sexual and reproductive health" and "eastern africa") ) OR AB ( (prepare and "promoting sexual and reproductive health" and "eastern africa") )

S66 TI "power up, speak out" OR AB "power up, speak out"

S65 TI "papa reto" OR AB "papa reto"

S64 TI "my voice, my choice" OR AB "my voice, my choice"

S63 TI "mentors in violence prevention" OR AB "mentors in violence prevention"

S62 TI ( ("Media Aware" or mediaaware) ) OR AB ( ("Media Aware" or mediaaware) )

S61 TI "mask of love" OR AB "mask of love"

S60 TI "love hurts" OR AB "love hurts"

S59 TI "love doesn't hurt" OR AB "love doesn't hurt"

S58 TI lights4violence OR AB lights4violence

S57 TI "let us protect our future" OR AB "let us protect our future"

S56 TI ( ("long live love" or "long live love+") ) OR AB ( ("long live love" or "long live love+") )

S55 TI "La máscara del amor" OR AB "La máscara del amor"

S54 TI "katie brown educational program" OR AB "katie brown educational program"

S53 TI "Juntos opuestos a la violence entre novios" OR AB "Juntos opuestos a la violence entre novios"

S52 TI IMPower OR AB IMPower

S51 TI "human relationships education" OR AB "human relationships education"

S50 TI ("healthy relationships") OR AB ("healthy relationships")

S49 TI ( (greendot or "green dot") ) OR AB ( (greendot or "green dot") )

S48 TI "green acres high" OR AB "green acres high"

S47 TI "good schools toolkit" OR AB "good schools toolkit"

S46 TI go girls initiative OR AB go girls initiative

S45 TI "gender equity movement in schools" OR AB "gender equity movement in schools"

S44 TI "Fourth R" OR AB "Fourth R"

S43 TI ("filles et garcons" N2 "en route pour l'egalite") OR AB ("filles et garcons" N2 "en route pour l'egalite")

S42 TI ( ("familias en nuestra escuela" or "families in our school") ) OR AB ( ("familias en nuestra escuela" or "families in our school") )

S41 TI "Expect Respect" OR AB "Expect Respect"

S40 TI "dating matters" OR AB "dating matters"

S39 TI "dat-e adolescence" OR AB "dat-e adolescence"

S38 TI "coaching boys into men" OR AB "coaching boys into men"

S37 TI ( (Connections and "dating and emotions curriculum") ) OR AB ( (Connections and "dating and emotions curriculum") )

S36 TI "connect with respect" OR AB "connect with respect"

S35 TI "chesterfield relate" OR AB "chesterfield relate"

S34 TI "change up project" OR AB "change up project"

S33 TI ("challenging violence" N2 "changing lives") OR AB ("challenging violence" N2 "changing lives")

S32 TI "building relationships in greater harmony together" OR AB "building relationships in greater harmony together"

S31 TI BITB-HSC OR AB BITB-HSC

S30 TI ( ("bringing in the bystander" and "high school") ) OR AB ( ("bringing in the bystander" and "high school") )

S29 TI ( ("break the cycle" AND (end N2 violence)) ) OR AB ( ("break the cycle" AND (end N2 violence)) )

S28 TI (Benzies N2 Batchies) OR AB (Benzies N2 Batchies)

S27 TI "architects of their own brain" OR AB "architects of their own brain"

S26 TI "athletes as leaders" OR AB "athletes as leaders"

S25 TI "alberta healthy youth relationships" OR AB "alberta healthy youth relationships"

S24 TI "5 W's of bullying intervention" OR AB "5 W's of bullying intervention"

S23 TI ( ((LGB or LGBT* or homosexual* or lesbian* or gay or bisexual* or queer* or transgender* or transsexual) N3 (abuse* or abusive or aggressi* or assault* or attack* or bully* or coerc* or cyberbully* or Femicid* or harass* or homicid* or injur* or manipulate* or murder* or rape* or threaten* or violen* or victimi?ation or revictimi?ation or re-victimi?ation)) ) OR AB ( ((LGB or LGBT* or homosexual* or lesbian* or gay or bisexual* or queer* or transgender* or transsexual) N3 (abuse* or abusive ...

S22 TI ( (homophobi* or transphobi* or biphobi* or homonegativ*) ) OR AB ( (homophobi* or transphobi* or biphobi* or homonegativ*) )

S21 TI ( ((coerc* or forced or unwanted or nonconsensual or non-consensual) N1 sex*) ) OR AB ( ((coerc* or forced or unwanted or nonconsensual or non-consensual) N1 sex*) )

S20 TI ( (sexual* N1 (abusive or aggressi* or assault* or attack* or bully* or coerc* or cyberbully* or femicid* or harass* or homicid* or injur* or manipulate* or murder* or rape* or threaten* or violen* or victimi?ation or revictimi?ation or re-victimi?ation)) ) OR AB ( (sexual* N1 (abusive or aggressi* or assault* or attack* or bully* or coerc* or cyberbully* or femicid* or harass* or homicid* or injur* or manipulate* or murder* or rape* or threaten* or violen* or victimi?ation or revictimi?ation ))

S19 TI ( ((boyfriend* or boy-friend* or girlfriend* or girl-friend*) N3 (abuse* or abusive or aggressi* or assault* or attack* or bully* or coerc* or cyberbully* or femicid* or harass* or homicid* or injur* or manipulate* or murder* or rape* or threaten* or violen* or victimi?ation or revictimi?ation or re-victimi?ation)) ) OR AB ( ((boyfriend* or boy-friend* or girlfriend* or girl-friend*) N3 (abuse* or abusive or aggressi* or assault* or attack* or bully* or coerc* or cyberbully* or femicid* or harass* or homicid* or injur* or manipulate* or murder* or rape* or threaten* or violen* or victimi?ation or revictimi?ation ))

S18 TI ( ((relationship*) N0 (abuse* or abusive or aggressi* or assault* or attack* or bully* or coerc* or cyberbully* or femicid* or harass* or homicid* or injur* or manipulate* or murder* or rape* or threaten* or violen* or victimi?ation or revictimi?ation or re-victimi?ation)) ) OR AB ( ((relationship*) N0 (abuse* or abusive or aggressi* or assault* or attack* or bully* or coerc* or cyberbully* or Femicid* or harass* or homicid* or injur* or manipulate* or murder* or rape* or threaten* or violen* or victimi?ation or revictimi?ation ))

S17 TI ( ((dating) N3 (abuse* or abusive or aggressi* or assault* or attack* or bully* or coerc* or cyberbully* or femicid* or harass* or homicid* or injur* or manipulate* or murder* or rape* or threaten* or violen* or victimi?ation or revictimi?ation or re-victimi?ation)) ) OR AB ( ((dating) N3 (abuse* or abusive or aggressi* or assault* or attack* or bully* or coerc* or cyberbully* or femicid* or harass* or homicid* or injur* or manipulate* or murder* or rape* or threaten* or violen* or victimi?at OR revictimi?ation ))

S16 TI "violence against women" OR AB "violence against women"

S15 TI ( (domestic N3 (abuse* or abusive or aggressi* or assault* or attack* or bully* or coerc* or cyberbully* or femicid* or harass* or homicid* or injur* or manipulate* or murder* or rape* or threaten* or violen* or victimi?ation or revictimi?ation or re-victimi?ation)) ) OR AB ( (domestic N3 (abuse* or abusive or aggressi* or assault* or attack* or bully* or coerc* or cyberbully* or femicid* or harass* or homicid* or injur* or manipulate* or murder* or rape* or threaten* or violen* or victimi?at OR revictimi?ation ))

S14 TI GBV OR AB GBV

S13 TI (gender* N3 violen*) OR AB (gender* N3 violen*)

S12 TI ("intimate partner violence" or "intimate partner abuse") OR AB ("intimate partner violence" or "intimate partner abuse")

S11 TI rape* OR AB (rape N0 myth*) OR AB (rape N1 acquaintance) OR AB (date N0 rape)

S10 (MH "Homophobia")

S9 (MH "Dating Violence")

S8 (MH "Domestic Violence")

S7 (MH "Coercion")

S6 (MH "Battered Men")

S5 (MH "Battered Women")

S4 (MH "Rape")

S3 (MH "Stalking")

S2 (MH "Gender-Based Violence")

S1 (MH "Intimate Partner Violence")

### **EBSCO ERIC (1966-)**

**Search completed: 01/06/2021**

S27 S25 AND S26 (2004)

S26 TI ( (intervention* or program* or prevent* or instruct* or strateg* or curricul* or project* or initiative*) ) OR AB ( (intervention* or program* or prevent* or instruct* or strateg* or curricul* or project* or initiative*) ) (883,243)

S25 S19 AND S24 (3,247)

S24 S20 OR S21 OR S22 OR S23 (628,251)

S23 TI school* OR AB school* OR SO school* (569,669)

S22 DE "Elementary School Students" OR DE "Middle School Students" OR DE "Secondary School Students" OR DE "High School Students" OR DE "Junior High School Students" (123,560)

S21 DE "School Health Services" (2,517)

S20 DE "Schools" OR DE "Boarding Schools" OR DE "Residential Schools" OR DE "Disadvantaged Schools" OR DE "Elementary Schools" OR DE "Middle Schools" OR DE "Nursery Schools" OR DE "Private Schools" OR DE "Public Schools" OR DE "Regional Schools" OR DE "Rural Schools" OR DE "Secondary Schools" OR DE "High Schools" OR DE "Junior High Schools" OR DE "Single Sex Schools" OR DE "Slum Schools" OR DE "Small Schools" OR DE "State Schools" OR DE "Suburban Schools" OR DE "Traditional Schools" OR DE "Urban Schools" (189,510)

S19 S1 OR S2 OR S3 OR S4 OR S5 OR S6 OR S7 OR S8 OR S9 OR S10 OR S11 OR S12 OR S13 OR S14 OR S15 OR S16 OR S17 OR S18 (9,157)

S18 TI ( ("5 W's of bullying intervention" OR "alberta healthy youth relationships" OR "athletes as leaders" OR "architects of their own brain" OR (Benzies NEAR/2 Batchies) OR ("break the cycle" and (end* NEAR/2 violence) ) OR ("bringing in the bystander" and "high school") OR BITB-HSC OR "building relationships in greater harmony together" OR ("challenging violence" NEAR/2 "changing lives") OR "change up project" OR "chesterfield relate" OR "connect with respect" OR (Connections and "dating and emotions curriculum") OR "coaching boys into men" OR "dat-e adolescence" OR "dating matters" OR "Expect Respect" OR ("familias en nuestra escuela" or "families in our school") OR ("filles et garcons" NEAR/2 "en route pour l'egalite") OR "Fourth R" OR "gender equity movement in schools" OR (“go girls initiative”) OR "good schools toolkit" OR "green acres high" OR (greendot or "green dot") OR ("healthy relationships") OR "human relationships education" OR IMPower OR "Juntos opuestos a la violence entre novios" OR "katie brown educational program" OR "La máscara del amor" OR ("long live love" or "long live love+") OR "let us protect our future" OR lights4violence OR "love doesn't hurt" OR "love hurts" OR "mask of love" OR ("Media Aware" or mediaaware) OR "mentors in violence prevention" OR "my voice, my choice" OR “papa reto" OR “power up, speak out!" OR (prepare and "promoting sexual and reproductive health" and "eastern africa") OR "eliminating coercion and pressure in adolescent relationships" OR "previo program" OR "project connect" OR "project pride" OR "project respect" OR R4Respect OR "reduction of stigma in schools" OR "relaciones romanticas constructivas" OR "relationships without fear" OR "respectful relationships" OR "Safe Dates" OR "safe schools" OR "school health center healthy adolescent relationship program" OR "Shifting boundaries" OR ("Second Step" and (program* or intervention*) ) OR "skillz street" OR skhokho OR SS-SSTP OR "start strong initiative" OR ("stay in love" or "stay in love+") OR TakeCARE OR "Teen choices" OR "trust education project" OR DaVIPoP OR "young men initiative" OR ("zero tolerance" and respect and project)) ) OR AB ( ("5 W's of bullying intervention" OR "alberta healthy youth relationships" OR "athletes as leaders" OR "architects of their own brain" OR (Benzies NEAR/2 Batchies) OR ("break the cycle" and (end* NEAR/2 violence) ) OR ("bringing in the bystander" and "high school") OR BITB-HSC OR "building relationships in greater harmony together" OR ("challenging violence" NEAR/2 "changing lives") OR "change up project" OR "chesterfield relate" OR "connect with respect" OR (Connections and "dating and emotions curriculum") OR "coaching boys into men" OR "dat-e adolescence" OR "dating matters" OR "Expect Respect" OR ("familias en nuestra escuela" or "families in our school") OR ("filles et garcons" NEAR/2 "en route pour l'egalite") OR "Fourth R" OR "gender equity movement in schools" OR (“go girls initiative”) OR "good schools toolkit" OR "green acres high" OR (greendot or "green dot") OR ("healthy relationships") OR "human relationships education" OR IMPower OR "Juntos opuestos a la violence entre novios" OR "katie brown educational program" OR "La máscara del amor" OR ("long live love" or "long live love+") OR "let us protect our future" OR lights4violence OR "love doesn't hurt" OR "love hurts" OR "mask of love" OR ("Media Aware" or mediaaware) OR "mentors in violence prevention" OR "my voice, my choice" OR “papa reto" OR “power up, speak out!" OR (prepare and "promoting sexual and reproductive health" and "eastern africa") OR "eliminating coercion and pressure in adolescent relationships" OR "previo program" OR "project connect" OR "project pride" OR "project respect" OR R4Respect OR "reduction of stigma in schools" OR "relaciones romanticas constructivas" OR "relationships without fear" OR "respectful relationships" OR "Safe Dates" OR "safe schools" OR "school health center healthy adolescent relationship program" OR "Shifting boundaries" OR ("Second Step" and (program* or intervention*) ) OR "skillz street" OR skhokho OR SS-SSTP OR "start strong initiative" OR ("stay in love" or "stay in love+") OR TakeCARE OR "Teen choices" OR "trust education project" OR DaVIPoP OR "young men initiative" OR ("zero tolerance" and respect and project)) ) (1,116)

S17 TI ( ((LGB or LGBT* or homosexual* or lesbian* or gay or bisexual* or queer* or transgender* or transsexual) N3 (abuse* or abusive or aggressi* or assault* or attack* or bully* or coerc* or cyberbully* or femicid* or harass* or homicid* or injur* or manipulate* or murder* or rape* or threaten* or violen* or victimi?ation or revictimi?ation or re-victimi?ation)) ) OR AB ( ((LGB or LGBT* or homosexual* or lesbian* or gay or bisexual* or queer* or transgender* or transsexual) N3 (abuse* or abusive or aggressi* or assault* or attack* or bully* or coerc* or cyberbully* or femicid* or harass* or homicid* or injur* or manipulate* or murder* or rape* or threaten* or violen* or victimi?ation or revictimi?ation or re-victimi?ation)) ) (281)

S16 TI ( (homophobi* or transphobi* or biphobi* or homonegativ*) ) OR AB ( (homophobi* or transphobi* or biphobi* or homonegativ*) ) (967)

S15 TI ( ((coerc* or forced or unwanted or nonconsensual or non-consensual) N1 sex*) ) OR AB ( ((coerc* or forced or unwanted or nonconsensual or non-consensual) N1 sex*) ) (316)

S14 TI ( (sexual* N1 (abusive or aggressi* or assault* or attack* or bully* or coerc* or cyberbully* or femicid* or harass* or homicid* or injur* or manipulate* or murder* or rape* or threaten* or violen* or victimi?ation or revictimi?ation or re-victimi?ation)) ) OR AB ( (sexual* N1 (abusive or aggressi* or assault* or attack* or bully* or coerc* or cyberbully* or femicid* or harass* or homicid* or injur* or manipulate* or murder* or rape* or threaten* or violen* or victimi?ation or revictimi?ation )) (3,429)

S13 TI ( ((boyfriend* or boy-friend* or girlfriend* or girl-friend*) N3 (abuse* or abusive or aggressi* or assault* or attack* or bully* or coerc* or cyberbully* or femicid* or harass* or homicid* or injur* or manipulate* or murder* or rape* or threaten* or violen* or victimi?ation or revictimi?ation or re-victimi?ation)) ) OR AB ( ((boyfriend* or boy-friend* or girlfriend* or girl-friend*) N3 (abuse* or abusive or aggressi* or assault* or attack* or bully* or coerc* or cyberbully* or femicid* or harass* or homicid* or injur* or manipulate* or murder* or rape* or threaten* or violen* or victimi?ation or revictimi?ation )) (28)

S12 TI ( ((relationship*) N0 (abuse* or abusive or aggressi* or assault* or attack* or bully* or coerc* or cyberbully* or Femicid* or harass* or homicid* or injur* or manipulate* or murder* or rape* or threaten* or violen* or victimi?ation or revictimi?ation or re-victimi?ation)) ) OR AB ( ((relationship*) N0 (abuse* or abusive or aggressi* or assault* or attack* or bully* or coerc* or cyberbully* or Femicid* or harass* or homicid* or injur* or manipulate* or murder* or rape* or threaten* or violen* or victimi?ation or revictimi?ation )) (276)

S11 TI ( ((dating) N3 (abuse* or abusive or aggressi* or assault* or attack* or bully* or coerc* or cyberbully* or femicid* or harass* or homicid* or injur* or manipulate* or murder* or rape* or threaten* or violen* or victimi?ation or revictimi?ation or re-victimi?ation)) ) OR AB ( ((dating) N3 (abuse* or abusive or aggressi* or assault* or attack* or bully* or coerc* or cyberbully* or femicid* or harass* or homicid* or injur* or manipulate* or murder* or rape* or threaten* or violen* or victimi?ation or revictimi?ation )) (409)

S10 TI "violence against women" OR AB "violence against women" (261)

S9 TI ( (domestic N3 (abuse* or abusive or aggressi* or assault* or attack* or bully* or coerc* or cyberbully* or femicid* or harass* or homicid* or injur* or manipulate* or murder* or rape* or threaten* or violen* or victimi?ation or revictimi?ation or re-victimi?ation)) ) OR AB ( (domestic N3 (abuse* or abusive or aggressi* or assault* or attack* or bully* or coerc* or cyberbully* or femicid* or harass* or homicid* or injur* or manipulate* or murder* or rape* or threaten* or violen* or victimi?ation or revictimi?ation )) (1,222)

S8 TI SRGBV OR AB SRGBV (10)

S7 TI GBV OR AB GBV (16)

S6 TI gender* N3 violen* OR AB gender* N3 violen* (342)

S5 TI ("intimate partner violence" or "intimate partner abuse") OR AB ("intimate partner violence" or "intimate partner abuse") (523)

S4 TI rape* OR AB (rape N0 myth*) OR AB (rape N1 acquaintance) OR AB (date N0 rape) (618)

S3 TI ( stalking or stalker* ) OR AB ( stalking or stalker* ) (183)

S2 DE "Sexual Harassment" (1,742)

S1 DE "Rape"

### **EBSCO Education Research Complete**

**Search completed: 01/06/2021**

S26 S24 AND S25 (1939)

S25 TI ( (intervention* or program* or prevent* or instruct* or strateg* or curricul* or project* or initiative*) ) OR AB ( (intervention* or program* or prevent* or instruct* or strateg* or curricul* or project* or initiative*) ) (939,147)

S24 S18 AND S23 (4,714)

S23 S19 OR S20 OR S21 OR S22 (969,570)

S22 TI school* OR AB school* OR SO school* (955,501)

S21 DE "BOARDING school students" OR DE "MIDDLE school students" OR DE "PRIVATE school students" OR DE "SCHOOL children" OR DE "SECONDARY school students" OR DE "SIXTH form students" (36,836)

S20 DE "SCHOOL health services" (2,520)

S19 DE "SCHOOLS" OR DE "BOARDING schools" OR DE "BRITISH schools" OR DE "DAY schools" OR DE "DISADVANTAGED schools" OR DE "ELEMENTARY schools" OR DE "FAILING schools" OR DE "PRIMARY schools" OR DE "PRIVATE schools" OR DE "PUBLIC schools" OR DE "RURAL schools" OR DE "SECONDARY schools" OR DE "SINGLE sex schools" OR DE "TRADITIONAL schools" (89,187)

S18 S1 OR S2 OR S3 OR S4 OR S5 OR S6 OR S7 OR S8 OR S9 OR S10 OR S11 OR S12 OR S13 OR S14 OR S15 OR S16 or S17 (20,290)

S17 TI ( ("5 W's of bullying intervention" OR "alberta healthy youth relationships" OR "athletes as leaders" OR "architects of their own brain" OR (Benzies NEAR/2 Batchies) OR ("break the cycle" and (end* NEAR/2 violence) ) OR ("bringing in the bystander" and "high school") OR BITB-HSC OR "building relationships in greater harmony together" OR ("challenging violence" NEAR/2 "changing lives") OR "change up project" OR "chesterfield relate" OR "connect with respect" OR (Connections and "dating and emotions curriculum") OR "coaching boys into men" OR "dat-e adolescence" OR "dating matters" OR "Expect Respect" OR ("familias en nuestra escuela" or "families in our school") OR ("filles et garcons" NEAR/2 "en route pour l'egalite") OR "Fourth R" OR "gender equity movement in schools" OR (“go girls initiative”) OR "good schools toolkit" OR "green acres high" OR (greendot or "green dot") OR ("healthy relationships") OR "human relationships education" OR IMPower OR "Juntos opuestos a la violence entre novios" OR "katie brown educational program" OR "La máscara del amor" OR ("long live love" or "long live love+") OR "let us protect our future" OR lights4violence OR "love doesn't hurt" OR "love hurts" OR "mask of love" OR ("Media Aware" or mediaaware) OR "mentors in violence prevention" OR "my voice, my choice" OR “papa reto" OR “power up, speak out!" OR (prepare and "promoting sexual and reproductive health" and "eastern africa") OR "eliminating coercion and pressure in adolescent relationships" OR "previo program" OR "project connect" OR "project pride" OR "project respect" OR R4Respect OR "reduction of stigma in schools" OR "relaciones romanticas constructivas" OR "relationships without fear" OR "respectful relationships" OR "Safe Dates" OR "safe schools" OR "school health center healthy adolescent relationship program" OR "Shifting boundaries" OR ("Second Step" and (program* or intervention*) ) OR "skillz street" OR skhokho OR SS-SSTP OR "start strong initiative" OR ("stay in love" or "stay in love+") OR TakeCARE OR "Teen choices" OR "trust education project" OR DaVIPoP OR "young men initiative" OR ("zero tolerance" and respect and project)) ) OR AB ( ("5 W's of bullying intervention" OR "alberta healthy youth relationships" OR "athletes as leaders" OR "architects of their own brain" OR (Benzies NEAR/2 Batchies) OR ("break the cycle" and (end* NEAR/2 violence) ) OR ("bringing in the bystander" and "high school") OR BITB-HSC OR "building relationships in greater harmony together" OR ("challenging violence" NEAR/2 "changing lives") OR "change up project" OR "chesterfield relate" OR "connect with respect" OR (Connections and "dating and emotions curriculum") OR "coaching boys into men" OR "dat-e adolescence" OR "dating matters" OR "Expect Respect" OR ("familias en nuestra escuela" or "families in our school") OR ("filles et garcons" NEAR/2 "en route pour l'egalite") OR "Fourth R" OR "gender equity movement in schools" OR (“go girls initiative”) OR "good schools toolkit" OR "green acres high" OR (greendot or "green dot") OR ("healthy relationships") OR "human relationships education" OR IMPower OR "Juntos opuestos a la violence entre novios" OR "katie brown educational program" OR "La máscara del amor" OR ("long live love" or "long live love+") OR "let us protect our future" OR lights4violence OR "love doesn't hurt" OR "love hurts" OR "mask of love" OR ("Media Aware" or mediaaware) OR "mentors in violence prevention" OR "my voice, my choice" OR “papa reto" OR “power up, speak out!" OR (prepare and "promoting sexual and reproductive health" and "eastern africa") OR "eliminating coercion and pressure in adolescent relationships" OR "previo program" OR "project connect" OR "project pride" OR "project respect" OR R4Respect OR "reduction of stigma in schools" OR "relaciones romanticas constructivas" OR "relationships without fear" OR "respectful relationships" OR "Safe Dates" OR "safe schools" OR "school health center healthy adolescent relationship program" OR "Shifting boundaries" OR ("Second Step" and (program* or intervention*) ) OR "skillz street" OR skhokho OR SS-SSTP OR "start strong initiative" OR ("stay in love" or "stay in love+") OR TakeCARE OR "Teen choices" OR "trust education project" OR DaVIPoP OR "young men initiative" OR ("zero tolerance" and respect and project)) ) (1,367)

S16 TI ( ((LGB or LGBT* or homosexual* or lesbian* or gay or bisexual* or queer* or transgender* or transsexual*) N3 (abuse* or abusive or aggressi* or assault* or attack* or bully* or coerc* or cyberbully* or Femicid* or harass* or homicid* or injur* or manipulate* or murder* or rape* or threaten* or violen* or victimi?ation or revictimi?ation or re-victimi?ation)) ) OR AB ( ((LGB or LGBT* or homosexual* or lesbian* or gay or bisexual* or queer* or transgender* or transsexual*) N3 (abuse* or abusive or aggressi* or assault* or attack* or bully* or coerc* or cyberbully* or femicid* or harass* or homicid* or injur* or manipulate* or murder* or rape* or threaten* or violen* or victimi?ation or revictimi?ation)) (804)

S15 TI ( (homophobi* or transphobi* or biphobi* or homonegativ*) ) OR AB ( (homophobi* or transphobi* or biphobi* or homonegativ*) ) (1,874)

S14 TI ( ((coerc* or forced or unwanted or nonconsensual or non-consensual) N1 sex*) ) OR AB ( ((coerc* or forced or unwanted or nonconsensual or non-consensual) N1 sex*) ) (796)

S13 TI ( (sexual* N1 (abusive or aggressi* or assault* or attack* or bully* or coerc* or cyberbully* or femicid* or harass* or homicid* or injur* or manipulate* or murder* or rape* or threaten* or violen* or victimi?ation or revictimi?ation or re-victimi?ation)) ) OR AB ( (sexual* N1 (abusive or aggressi* or assault* or attack* or bully* or coerc* or cyberbully* or femicid* or harass* or homicid* or injur* or manipulate* or murder* or rape* or threaten* or violen* or victimi?ation or revictimi?ation)) (8,651)

S12 TI ( ((boyfriend* or boy-friend* or girlfriend* or girl-friend*) N3 (abuse* or abusive or aggressi* or assault* or attack* or bully* or coerc* or cyberbully* or femicid* or harass* or homicid* or injur* or manipulate* or murder* or rape* or threaten* or violen* or victimi?ation or revictimi?ation or re-victimi?ation)) ) OR AB ( ((boyfriend* or boy-friend* or girlfriend* or girl-friend*) N3 (abuse* or abusive or aggressi* or assault* or attack* or bully* or coerc* or cyberbully* or femicid* or harass* or homicid* or injur* or manipulate* or murder* or rape* or threaten* or violen* or victimi?ation or revictimi?ation)) (53)

S11 TI ( ((relationship*) N0 (abuse* or abusive or aggressi* or assault* or attack* or bully* or coerc* or cyberbully* or Femicid* or harass* or homicid* or injur* or manipulate* or murder* or rape* or threaten* or violen* or victimi?ation or revictimi?ation or re-victimi?ation)) ) OR AB ( ((relationship*) N0 (abuse* or abusive or aggressi* or assault* or attack* or bully* or coerc* or cyberbully* or Femicid* or harass* or homicid* or injur* or manipulate* or murder* or rape* or threaten* or violen* or victimi?ation or revictimi?ation)) (744)

S10 TI ( ((dating) N3 (abuse* or abusive or aggressi* or assault* or attack* or bully* or coerc* or cyberbully* or femicid* or harass* or homicid* or injur* or manipulate* or murder* or rape* or threaten* or violen* or victimi?ation or revictimi?ation or re-victimi?ation)) ) OR AB ( ((dating) N3 (abuse* or abusive or aggressi* or assault* or attack* or bully* or coerc* or cyberbully* or femicid* or harass* or homicid* or injur* or manipulate* or murder* or rape* or threaten* or violen* or victimi?at or revictimi?ation)) (998)

S9 TI "violence against women" OR AB "violence against women" (805)

S8 TI ( (domestic N3 (abuse* or abusive or aggressi* or assault* or attack* or bully* or coerc* or cyberbully* or femicid* or harass* or homicid* or injur* or manipulate* or murder* or rape* or threaten* or violen* or victimi?ation or revictimi?ation or re-victimi?ation)) ) OR AB ( (domestic N3 (abuse* or abusive or aggressi* or assault* or attack* or bully* or coerc* or cyberbully* or femicid* or harass* or homicid* or injur* or manipulate* or murder* or rape* or threaten* or violen* or victimi?at or revictimi?ation)) (2,927)

S7 TI SRGBV OR AB SRGBV (3)

S6 TI GBV OR AB GBV (33)

S5 TI (gender* N3 violen*) OR AB (gender* N3 violen*) (879)

S4 TI ("intimate partner violence" or "intimate partner abuse") OR AB ("intimate partner violence" or "intimate partner abuse") (2,304)

S3 TI rape* OR AB (rape N0 myth*) OR AB (rape N1 acquaintance) OR AB (date N0 rape) (1,643)

S2 DE "HOMOPHOBIA in schools" OR DE "HOMOPHOBIA in high schools" OR DE "BIPHOBIA in schools" (176)

S1 DE "SEXUAL harassment in education" (719)

### **ProQuest Dissertations and Theses**

**Search completed: 2/06/2021**

(AB(school*) OR TI(school*)) AND (TI(intervention* OR program* OR prevent* OR instruct* OR strateg* OR curricul* OR project* OR initiative*) OR AB(intervention* OR program* OR prevent* OR instruct* OR strateg* OR curricul* OR project* OR initiative*)) AND (ti(rape* OR "partner violence" OR "partner abuse" OR "violence against women" OR (gender* NEAR/3 violen*)) OR ti(((domestic OR dating OR relationship* OR sexual* OR LGB OR LGBT OR homosexual* OR lesbian* OR gay OR bisexual* OR queer* OR transgender* OR transsexual*) NEAR/3 (abuse OR abusive OR aggressi* OR assault* OR attack* OR bully* OR coerc* OR cyberbully* OR femicid* OR harass* OR homicid* OR injur* OR manipulat* OR murder* OR threaten* OR violen* OR victimi?ation OR revictimi?ation OR re-victimi?ation))) OR (ti(homophobi* OR biphobi* OR transphobi* OR homonegativ*) OR ti((coerc* OR forced OR unwanted OR nonconsensual OR non-consensual) NEAR/1 sex*)) OR (TI("5 W's of bullying intervention" OR "alberta healthy youth relationships" OR "athletes as leaders" OR "architects of their own brain" OR (Benzies NEAR/2 Batchies) OR ("break the cycle" AND (end* NEAR/2 violence)) OR ("bringing in the bystander" AND "high school") OR BITB-HSC OR "building relationships in greater harmony together" OR ("challenging violence" NEAR/2 "changing lives") OR "change up project" OR "chesterfield relate" OR "connect with respect" OR (Connections AND "dating and emotions curriculum") OR "coaching boys into men" OR "dat-e adolescence" OR "dating matters" OR "Expect Respect" OR ("familias en nuestra escuela" OR "families in our school") OR ("filles et garcons" NEAR/2 "en route pour l'egalite") OR "Fourth R" OR "gender equity movement in schools" OR ("go girls initiative") OR "good schools toolkit" OR "green acres high" OR (greendot OR "green dot") OR ("healthy relationships") OR "human relationships education" OR IMPower OR "Juntos opuestos a la violence entre novios" OR "katie brown educational program" OR "La máscara del amor" OR ("long live love" OR "long live love+") OR "let us protect our future" OR lights4violence OR "love doesn't hurt" OR "love hurts" OR "mask of love" OR ("Media Aware" OR mediaaware) OR "mentors in violence prevention" OR "my voice, my choice" OR "papa reto" OR "power up, speak out!" OR (prepare AND "promoting sexual and reproductive health" AND "eastern africa") OR "eliminating coercion and pressure in adolescent relationships" OR "previo program" OR "project connect" OR "project pride" OR "project respect" OR R4Respect OR "reduction of stigma in schools" OR "relaciones romanticas constructivas" OR "relationships without fear" OR "respectful relationships" OR "Safe Dates" OR "safe schools" OR "school health center healthy adolescent relationship program" OR "Shifting boundaries" OR ("Second Step" AND (program* OR intervention*)) OR "skillz street" OR skhokho OR SS-SSTP OR "start strong initiative" OR ("stay in love" OR "stay in love+") OR TakeCARE OR "Teen choices" OR "trust education project" OR DaVIPoP OR "young men initiative" OR ("zero tolerance" AND respect AND project)) OR TI("5 W's of bullying intervention" OR "alberta healthy youth relationships" OR "athletes as leaders" OR "architects of their own brain" OR (Benzies NEAR/2 Batchies) OR ("break the cycle" AND (end* NEAR/2 violence)) OR ("bringing in the bystander" AND "high school") OR BITB-HSC OR "building relationships in greater harmony together" OR ("challenging violence" NEAR/2 "changing lives") OR "change up project" OR "chesterfield relate" OR "connect with respect" OR (Connections AND "dating and emotions curriculum") OR "coaching boys into men" OR "dat-e adolescence" OR "dating matters" OR "Expect Respect" OR ("familias en nuestra escuela" OR "families in our school") OR ("filles et garcons" NEAR/2 "en route pour l'egalite") OR "Fourth R" OR "gender equity movement in schools" OR ("go girls initiative") OR "good schools toolkit" OR "green acres high" OR (greendot OR "green dot") OR ("healthy relationships") OR "human relationships education" OR IMPower OR "Juntos opuestos a la violence entre novios" OR "katie brown educational program" OR "La máscara del amor" OR ("long live love" OR "long live love+") OR "let us protect our future" OR lights4violence OR "love doesn't hurt" OR "love hurts" OR "mask of love" OR ("Media Aware" OR mediaaware) OR "mentors in violence prevention" OR "my voice, my choice" OR "papa reto" OR "power up, speak out!" OR (prepare AND "promoting sexual and reproductive health" AND "eastern africa") OR "eliminating coercion and pressure in adolescent relationships" OR "previo program" OR "project connect" OR "project pride" OR "project respect" OR R4Respect OR "reduction of stigma in schools" OR "relaciones romanticas constructivas" OR "relationships without fear" OR "respectful relationships" OR "Safe Dates" OR "safe schools" OR "school health center healthy adolescent relationship program" OR "Shifting boundaries" OR ("Second Step" AND (program* OR intervention*)) OR "skillz street" OR skhokho OR SS-SSTP OR "start strong initiative" OR ("stay in love" OR "stay in love+") OR TakeCARE OR "Teen choices" OR "trust education project" OR DaVIPoP OR "young men initiative" OR ("zero tolerance" AND respect AND project))))

### **ProQuest Australian Education Index (1977-)**

**Search completed: 2/06/2021**

(((ab(rape* OR "partner violence" OR "partner abuse" OR "violence against women" OR (gender* NEAR/3 violen*)) OR ti(rape* OR "partner violence" OR "partner abuse" OR "violence against women" OR (gender* NEAR/3 violen*))) OR (ab(((domestic OR dating OR relationship* OR sexual* OR LGB OR LGBT OR homosexual* OR lesbian* OR gay OR bisexual* OR queer* OR transgender* OR transsexual*) NEAR/2 (abuse OR abusive OR aggressi* OR assault* OR attack* OR bully* OR coerc* OR cyberbully* OR femicid* OR harass* OR homicid* OR injur* OR manipulat* OR murder* OR threaten* OR violen* OR victimi?ation OR revictimi?ation OR re-victimi?ation))) OR ti(((domestic OR dating OR relationship* OR sexual* OR LGB OR LGBT OR homosexual* OR lesbian* OR gay OR bisexual* OR queer* OR transgender* OR transsexual*) NEAR/3 (abuse OR abusive OR aggressi* OR assault* OR attack* OR bully* OR coerc* OR cyberbully* OR femicid* OR harass* OR homicid* OR injur* OR manipulat* OR murder* OR threaten* OR violen* OR victimi?ation OR revictimi?ation OR re-victimi?ation)))) OR (ab(homophobi* OR biphobi* OR transphobi* OR homonegativ*) OR ti(homophobi* OR biphobi* OR transphobi* OR homonegativ*)) OR (ab(((coerc* OR forced OR unwanted OR nonconsensual OR non-consensual) NEAR/1 sex*)) OR ti(((coerc* OR forced OR unwanted OR nonconsensual OR non-consensual) NEAR/1 sex*))) OR (MAINSUBJECT.EXACT("Rape") OR MAINSUBJECT.EXACT("Sexual harassment") OR MAINSUBJECT.EXACT("Battered women") OR MAINSUBJECT.EXACT("Homophobia"))) OR (ab(("5 W's of bullying intervention" OR "alberta healthy youth relationships" OR "athletes as leaders" OR "architects of their own brain" OR (Benzies NEAR/2 Batchies) OR ("break the cycle" AND (end* NEAR/2 violence)) OR ("bringing in the bystander" AND "high school") OR BITB-HSC OR "building relationships in greater harmony together" OR ("challenging violence" NEAR/2 "changing lives") OR "change up project" OR "chesterfield relate" OR "connect with respect" OR (Connections AND "dating and emotions curriculum") OR "coaching boys into men" OR "dat-e adolescence" OR "dating matters" OR "Expect Respect" OR ("familias en nuestra escuela" OR "families in our school") OR ("filles et garcons" NEAR/2 "en route pour l'egalite") OR "Fourth R" OR "gender equity movement in schools" OR ("go girls initiative") OR "good schools toolkit" OR "green acres high" OR (greendot OR "green dot") OR ("healthy relationships") OR "human relationships education" OR IMPower OR "Juntos opuestos a la violence entre novios" OR "katie brown educational program" OR "La máscara del amor" OR ("long live love" OR "long live love+") OR "let us protect our future" OR lights4violence OR "love doesn't hurt" OR "love hurts" OR "mask of love" OR ("Media Aware" OR mediaaware) OR "mentors in violence prevention" OR "my voice, my choice" OR "papa reto" OR "power up, speak out!" OR (prepare AND "promoting sexual and reproductive health" AND "eastern africa") OR "eliminating coercion and pressure in adolescent relationships" OR "previo program" OR "project connect" OR "project pride" OR "project respect" OR R4Respect OR "reduction of stigma in schools" OR "relaciones romanticas constructivas" OR "relationships without fear" OR "respectful relationships" OR "Safe Dates" OR "safe schools" OR "school health center healthy adolescent relationship program" OR "Shifting boundaries" OR ("Second Step" AND (program* OR intervention*)) OR "skillz street" OR skhokho OR SS-SSTP OR "start strong initiative" OR ("stay in love" OR "stay in love+") OR TakeCARE OR "Teen choices" OR "trust education project" OR DaVIPoP OR "young men initiative" OR ("zero tolerance" AND respect AND project))) OR ti(("5 W's of bullying intervention" OR "alberta healthy youth relationships" OR "athletes as leaders" OR "architects of their own brain" OR (Benzies NEAR/2 Batchies) OR ("break the cycle" AND (end* NEAR/2 violence)) OR ("bringing in the bystander" AND "high school") OR BITB-HSC OR "building relationships in greater harmony together" OR ("challenging violence" NEAR/2 "changing lives") OR "change up project" OR "chesterfield relate" OR "connect with respect" OR (Connections AND "dating and emotions curriculum") OR "coaching boys into men" OR "dat-e adolescence" OR "dating matters" OR "Expect Respect" OR ("familias en nuestra escuela" OR "families in our school") OR ("filles et garcons" NEAR/2 "en route pour l'egalite") OR "Fourth R" OR "gender equity movement in schools" OR ("go girls initiative") OR "good schools toolkit" OR "green acres high" OR (greendot OR "green dot") OR ("healthy relationships") OR "human relationships education" OR IMPower OR "Juntos opuestos a la violence entre novios" OR "katie brown educational program" OR "La máscara del amor" OR ("long live love" OR "long live love+") OR "let us protect our future" OR lights4violence OR "love doesn't hurt" OR "love hurts" OR "mask of love" OR ("Media Aware" OR mediaaware) OR "mentors in violence prevention" OR "my voice, my choice" OR "papa reto" OR "power up, speak out!" OR (prepare AND "promoting sexual and reproductive health" AND "eastern africa") OR "eliminating coercion and pressure in adolescent relationships" OR "previo program" OR "project connect" OR "project pride" OR "project respect" OR R4Respect OR "reduction of stigma in schools" OR "relaciones romanticas constructivas" OR "relationships without fear" OR "respectful relationships" OR "Safe Dates" OR "safe schools" OR "school health center healthy adolescent relationship program" OR "Shifting boundaries" OR ("Second Step" AND (program* OR intervention*)) OR "skillz street" OR skhokho OR SS-SSTP OR "start strong initiative" OR ("stay in love" OR "stay in love+") OR TakeCARE OR "Teen choices" OR "trust education project" OR DaVIPoP OR "young men initiative" OR ("zero tolerance" AND respect AND project))))) AND ((ab(school*) OR ti(school*)) OR (MAINSUBJECT.EXACT("Disadvantaged schools") OR MAINSUBJECT.EXACT("Nursery schools") OR MAINSUBJECT.EXACT("Schools") OR MAINSUBJECT.EXACT("Primary schools") OR MAINSUBJECT.EXACT("Boarding schools") OR MAINSUBJECT.EXACT("Primary secondary schools") OR MAINSUBJECT.EXACT("Rural schools") OR MAINSUBJECT.EXACT("Middle schools") OR MAINSUBJECT.EXACT("Single sex schools") OR MAINSUBJECT.EXACT("Day schools") OR MAINSUBJECT.EXACT("Secondary schools"))) AND (ab((intervention* OR program* OR prevent* OR instruct* OR strateg* OR curricul* OR project* OR initiative*)) OR ti((intervention* OR program* OR prevent* OR instruct* OR strateg* OR curricul* OR project* OR initiative*)))

### **ProQuest Sociological Abstracts (1952-)**

**Search completed: 2/06/2021**

(((ab(rape* OR "partner violence" OR "partner abuse" OR "violence against women" OR (gender* NEAR/3 violen*)) OR ti(rape* OR "partner violence" OR "partner abuse" OR "violence against women" OR (gender* NEAR/3 violen*))) OR (ab(((domestic OR dating OR relationship* OR sexual* OR LGB OR LGBT OR homosexual* OR lesbian* OR gay OR bisexual* OR queer* OR transgender* OR transsexual*) NEAR/2 (abuse OR abusive OR aggressi* OR assault* OR attack* OR bully* OR coerc* OR cyberbully* OR femicid* OR harass* OR homicid* OR injur* OR manipulat* OR murder* OR threaten* OR violen* OR victimi?ation OR revictimi?ation OR re-victimi?ation))) OR ti(((domestic OR dating OR relationship* OR sexual* OR LGB OR LGBT OR homosexual* OR lesbian* OR gay OR bisexual* OR queer* OR transgender* OR transsexual*) NEAR/3 (abuse OR abusive OR aggressi* OR assault* OR attack* OR bully* OR coerc* OR cyberbully* OR femicid* OR harass* OR homicid* OR injur* OR manipulat* OR murder* OR threaten* OR violen* OR victimi?ation OR revictimi?ation OR re-victimi?ation)))) OR (ab(homophobi* OR biphobi* OR transphobi* OR homonegativ*) OR ti(homophobi* OR biphobi* OR transphobi* OR homonegativ*)) OR (ab(((coerc* OR forced OR unwanted OR nonconsensual OR non-consensual) NEAR/1 sex*)) OR ti(((coerc* OR forced OR unwanted OR nonconsensual OR non-consensual) NEAR/1 sex*))) OR (MAINSUBJECT.EXACT("Stalking") OR MAINSUBJECT.EXACT("Sexual assault") OR MAINSUBJECT.EXACT("Battered women") OR MAINSUBJECT.EXACT("Spouse abuse") OR MAINSUBJECT.EXACT("Homophobia"))) OR (ab(("5 W's of bullying intervention" OR "alberta healthy youth relationships" OR "athletes as leaders" OR "architects of their own brain" OR (Benzies NEAR/2 Batchies) OR ("break the cycle" AND (end* NEAR/2 violence)) OR ("bringing in the bystander" AND "high school") OR BITB-HSC OR "building relationships in greater harmony together" OR ("challenging violence" NEAR/2 "changing lives") OR "change up project" OR "chesterfield relate" OR "connect with respect" OR (Connections AND "dating and emotions curriculum") OR "coaching boys into men" OR "dat-e adolescence" OR "dating matters" OR "Expect Respect" OR ("familias en nuestra escuela" OR "families in our school") OR ("filles et garcons" NEAR/2 "en route pour l'egalite") OR "Fourth R" OR "gender equity movement in schools" OR ("go girls initiative") OR "good schools toolkit" OR "green acres high" OR (greendot OR "green dot") OR ("healthy relationships") OR "human relationships education" OR IMPower OR "Juntos opuestos a la violence entre novios" OR "katie brown educational program" OR "La máscara del amor" OR ("long live love" OR "long live love+") OR "let us protect our future" OR lights4violence OR "love doesn't hurt" OR "love hurts" OR "mask of love" OR ("Media Aware" OR mediaaware) OR "mentors in violence prevention" OR "my voice, my choice" OR "papa reto" OR "power up, speak out!" OR (prepare AND "promoting sexual and reproductive health" AND "eastern africa") OR "eliminating coercion and pressure in adolescent relationships" OR "previo program" OR "project connect" OR "project pride" OR "project respect" OR R4Respect OR "reduction of stigma in schools" OR "relaciones romanticas constructivas" OR "relationships without fear" OR "respectful relationships" OR "Safe Dates" OR "safe schools" OR "school health center healthy adolescent relationship program" OR "Shifting boundaries" OR ("Second Step" AND (program* OR intervention*)) OR "skillz street" OR skhokho OR SS-SSTP OR "start strong initiative" OR ("stay in love" OR "stay in love+") OR TakeCARE OR "Teen choices" OR "trust education project" OR DaVIPoP OR "young men initiative" OR ("zero tolerance" AND respect AND project))) OR ti(("5 W's of bullying intervention" OR "alberta healthy youth relationships" OR "athletes as leaders" OR "architects of their own brain" OR (Benzies NEAR/2 Batchies) OR ("break the cycle" AND (end* NEAR/2 violence)) OR ("bringing in the bystander" AND "high school") OR BITB-HSC OR "building relationships in greater harmony together" OR ("challenging violence" NEAR/2 "changing lives") OR "change up project" OR "chesterfield relate" OR "connect with respect" OR (Connections AND "dating and emotions curriculum") OR "coaching boys into men" OR "dat-e adolescence" OR "dating matters" OR "Expect Respect" OR ("familias en nuestra escuela" OR "families in our school") OR ("filles et garcons" NEAR/2 "en route pour l'egalite") OR "Fourth R" OR "gender equity movement in schools" OR ("go girls initiative") OR "good schools toolkit" OR "green acres high" OR (greendot OR "green dot") OR ("healthy relationships") OR "human relationships education" OR IMPower OR "Juntos opuestos a la violence entre novios" OR "katie brown educational program" OR "La máscara del amor" OR ("long live love" OR "long live love+") OR "let us protect our future" OR lights4violence OR "love doesn't hurt" OR "love hurts" OR "mask of love" OR ("Media Aware" OR mediaaware) OR "mentors in violence prevention" OR "my voice, my choice" OR "papa reto" OR "power up, speak out!" OR (prepare AND "promoting sexual and reproductive health" AND "eastern africa") OR "eliminating coercion and pressure in adolescent relationships" OR "previo program" OR "project connect" OR "project pride" OR "project respect" OR R4Respect OR "reduction of stigma in schools" OR "relaciones romanticas constructivas" OR "relationships without fear" OR "respectful relationships" OR "Safe Dates" OR "safe schools" OR "school health center healthy adolescent relationship program" OR "Shifting boundaries" OR ("Second Step" AND (program* OR intervention*)) OR "skillz street" OR skhokho OR SS-SSTP OR "start strong initiative" OR ("stay in love" OR "stay in love+") OR TakeCARE OR "Teen choices" OR "trust education project" OR DaVIPoP OR "young men initiative" OR ("zero tolerance" AND respect AND project))))) AND ((ab(school*) OR ti(school*)) OR (MAINSUBJECT.EXACT("Junior High Schools") OR MAINSUBJECT.EXACT("Secondary Schools") OR MAINSUBJECT.EXACT("Schools") OR MAINSUBJECT.EXACT("Elementary Schools") OR MAINSUBJECT.EXACT("High Schools"))) AND (ab((intervention* OR program* OR prevent* OR instruct* OR strateg* OR curricul* OR project* OR initiative*)) OR ti((intervention* OR program* OR prevent* OR instruct* OR strateg* OR curricul* OR project* OR initiative*)))

### Google Scholar searches

All searches conducted via Publish or Perish (Harzing) in the ‘keywords’ field. Searches were conducted between 29/04/2021 and 12/05/2021.

| **Search terms** | **Date searched** | **Number of records retrieved** | **No of records screened** | **No of records retained** |
| --- | --- | --- | --- | --- |
| 50:50 “no means no” intervention | 29/04/2021 | 73 | 73 | 0 |
| “Alberta Healthy Youth Relationships” AHYR Strategy | 29/04/2021 | 5 | 5 | 0 |
| “Athletes as leaders” school | 29/04/2021 | 119 | 119 | 0 |
| “Architects of their own brain” | 29/04/2021 | 23 | 23 | 1 |
| “Benzies & Batchies” | 29/04/2021 | 17 | 17 | 2 |
| “Break the cycle” “ending violence” program school | 29/04/2021 | 387 | (200 records - 20 pages) | 1 |
| “Building relationships in greater harmony together” | 29/04/2021 | 2 | 2 | 0 |
| “Challenging violence, changing lives” | 29/04/2021 | 29 | 29 | 3 |
| “Change up project” School | 29/04/2021 | 12 | 12 | 2 |
| “Chesterfield Relate” | 29/04/2021 | 4 | 4 | 1 |
| “Choices & consequences” school violence | 29/04/2021 | 43 | 43 | 0 |
| “Connect with respect” school gender-based violence | 29/04/2021 | 2 | 2 | 0 |
| Connections “Dating and emotions curriculum” | 29/04/2021 | 7 | 7 | 0 |
| “coaching boys into men” school | 29/04/2021 | 594 | 200 (20 pages) | 5 |
| “Dat-e adolescence” | 04/05/2021 | 34 | 34 | 0 |
| “Dating matters” school | 04/05/2021 | 371 | 200 (20 pages) | 7 |
| “dating violence prevention project curriculum” | 04/05/2021 | 2 | 2 | 0 |
| “expect respect” school prevention dating or sexual | 04/05/2021 | 1,180 | 200 (20 pages) | 6 |
| “familias en nuestra escuela” | 05/05/2021 | 10 | 10 | 0 |
| “filles et garcons en route pour l’egalite” | 05/05/2021 | 8 | 8 | 0 |
| “fourth R” dating violence OR healthy relationships | 05/05/2021 | 2380 | 200 (20 pages) | 3 |
| GEMS “gender equity movement in schools” | 05/05/2021 | 76 | 76 | 1 |
| “go for the gold” “relationship education” school | 05/05/2021 | 11 | 11 | 0 |
| “go girls! Initiative” | 05/05/2021 | 28 | 28 | 0 |
| “good schools toolkit” sexual violence | 05/05/2021 | 77 | 77 | 0 |
| “green acres high” | 05/05/2021 | 53 | 53 | 0 |
| “green dot” violence school | 05/05/2021 | 2200 | 200 (20 pages) | 2 |
| “healthy relationships programme” school | 05/05/2021 | 112 | 112 | 2 |
| “h and m programs” | 05/05/2021 | 3 | 3 | 0 |
| “human relationships education” HRE curriculum school | 05/05/2021 | 61 | 61 | 0 |
| “healthy relationships program” LGBTQ+ youth | 05/05/2021 | 48 | 48 | 1 |
| “Impower” school “no means no” | 5/05/2021 | 20 | 20 | 1 |
| “inter-ministerial national structural prevention trial” (INSTRUCT) | 05/05/2021 | 1 | 1 | 0 |
| “it’s your game” “keep it real” | 05/05/2021 | 147 | 147 | 0 |
| “Jesse” “video game” “violence prevention” school | 05/05/2021 | 107 | 107 | 0 |
| “Juntos opuestos a la violence entre novios” | 05/05/2021 | 3 | 3 | 0 |
| “together against dating violence” | 05/05/2021 | 6 | 6 | 0 |
| “Katie Brown educational program” | 05/05/2021 | 27 | 27 | 0 |
| “La Mascara del Amor” | 05/05/2021 | 191 | 191 | 0 |
| “Let us protect our future” | 05/05/2021 | 64 | 64 | 0 |
| Lights4violence | 05/05/2021 | 44 | 44 | 0 |
| “love doesn’t hurt” program | 06/05/2021 | 49 | 49 | 0 |
| “media aware” “media literacy education” school | 06/05/2021 | 54 | 54 | 1 |
| “Me & You” “dating violence” | 06/05/2021 | 18 | 18 | 1 |
| “mentors in violence prevention” school | 06/05/2021 | 1010 | 200 (20 pages) | 0 |
| “my voice, my choice” school | 06/05/2021 | 80 | 80 | 0 |
| “oficina namoro a primera vista” | 06/05/2021 | 2 | 2 | 0 |
| “papa reto” | 06/05/2021 | 3 | 3 | 0 |
| “parallel retreat” violence | 06/05/2021 | 10 | 10 | 0 |
| “pass it on” violence prevention program school | 06/05/2021 | 5720 | 200 (20 pages) | 0 |
| “power up, speak out” | 06/05/2021 | 1 | 1 | 0 |
| PREPARE promoting sexual and reproductive health among adolescents in south africa | 06/05/2021 | 2 | 2 | 0 |
| Pr:Epare (positive relationships eliminating coercion and pressure in adolescent relationships | 06/05/2021 | 34 | 34 | 0 |
| Previo program school “dating violence” | 06/05/2021 | 393 | 200 (20 pages) | 0 |
| “project connect” school nurse violence | 06/05/2021 | 259 | 200 (20 pages) | 0 |
| “project pride” school “dating violence” | 06/05/2021 | 16 | 16 | 0 |
| “project respect” school relationships education (limited to 2019-2021) | 06/05/2021 | 166 | 166 | 0 |
| R4Respect | 06/05/2021 | 60 | 60 | 1 |
| “Reduction of Stigma in Schools” | 06/05/2021 | 153 | 153 | 0 |
| “relacioners romanticas constructivas” | 10/05/2021 | 7 | 7 | 0 |
| “relationships without fear” school | 10/05/2021 | 297 | 200 (first 20 pages) | 2 |
| “respectful relationships” education school australia | 10/05/2021 | 8200 | 200 (first 20 pages) | 1 |
| “Rhode island teen dating violence prevention program” | 10/05/2021 | 8 | 8 | 0 |
| “safe dates” school “dating violence | 10/05/2021 | 2260 | 200 (first 20 pages) | 0 |
| “safe dates theater project” | 10/05/2021 | 4 | 4 | 1 |
| “safe schools” program lesbian gay | 10/05/2021 | 4710 | 200 (first 20 pages) | 1 |
| Saisir program “dating violence” school | 10/05/2021 | 74 | 74 | 0 |
| “school health center health adolescent relationships program” | 10/05/2021 | 2 | 2 | 0 |
| “school without violence” programme | 10/05/2021 | 114 | 114 | 0 |
| “scientist-practitioner program” “sexual violence” school | 10/05/2021 | 4 | 4 | 0 |
| “second step” “middle school” program sexual | 1005/2021 | 14,000 | 200 (first 20 pages) | 1 |
| “shifting boundaries” program “dating violence” school | 10/05/2021 | 477 | 200 (first 20 pages) | 1 |
| “skillz street” | 10/05/2021 | 23 | 23 | 1 |
| Skhokho violence | 11/05/2021 | 84 | 84 | 0 |
| “sources of strength” program violence school | 11/05/2021 | 7000 | 200 (first 20 pages) | 2 |
| Speak “rape myth acceptance” malo-juvera school | 11/05/2021 | 58 | 58 | 0 |
| “start strong” initiative dating violence | 11/05/2021 | 148 | 148 | 1 |
| “stay in love+” | 11/05/2021 | 5 | 5 | 1 |
| Takecare “bystander program” school | 11/05/2021 | 106 | 106 | 0 |
| “teen choices” “dating violence” school | 12/05/2021 | 87 | 87 | 0 |
| Tender “healthy relationships” education school | 12/05/2021 | 3890 | 200 (20 pages) | 1 |
| Texas Team’s “teen dating violence awareness” | 12/05/2021 | 40 | 40 | 0 |
| “trust education project” | 12/05/2021 | 12 | 12 | 0 |
| Twilight “true love” and you bibliotherapy dating school | 12/05/2021 | 19 | 19 | 0 |
| “Young men’s initiative” violence | 12/05/2021 | 53 | 53 | 0 |
| “zero tolerance” respect project school violence scotland | 12/05/2021 | 5000 | 200 (first 20 pages) | 1 |

### **Web site searches**

**VAWnet**[**https://vawnet.org**](https://vawnet.org) **Searched: 27/05/2021**
Browsed site: Content Topics > Intervention & Prevention. Filtered to Program Evaluation.
0 records to screen

USAID
https//www.usaid.gov/gbv
Searched 26/04/2021
Browsed reports.
0 records to screen

**Together for Girls**[**https://www.togetherforgirls.org/svsolutions-resource-hub**](https://www.togetherforgirls.org/svsolutions-resource-hub) **Searched: 26/04/2021**
Checked publications in SVSolutions Resource Hub (58 records). 0 records selected for screening.

**Global Working Group to Prevent DRGBV**[**https://www.ungei.org/knowledge-hub/school-related-gender-based-violence-srgbv**](https://www.ungei.org/knowledge-hub/school-related-gender-based-violence-srgbv) **Searched: 26/04/2021**
Reviewed publications in the Knowledge Hub:
School-related gender based violence > Case Studies (18). 4 records selected to screen.
School-related gender based violence > Monitoring and Evaluation Resources (22). 4 records selected to screen.

**Raising Voices**[**http://raisingvoices.org/innovation/generating-evidence/**](http://raisingvoices.org/innovation/generating-evidence/) **Checked for any publications: 26/04/2021**
0 records selected to screen

**Irish Consortium on Gender Based Violence**[**https://www.gbv.ie/**](https://www.gbv.ie/) **Searched: 26/04/2021**
Browsed Learning > Publications (20)
0 records selected to screen

**Break the Cycle
https://www.breakthecycle.org/
Searched: 26/04/2021**
Break the Cycle has ended its programs, and no publications are listed on the website.

**Equally Safe at Schools**[**https://www.rapecrisisscotland.org.uk/equally-safe-at-school**](https://www.rapecrisisscotland.org.uk/equally-safe-at-school) **Searched: 26/04/2021**0 records selected to screen

**National Criminal Justice Reference Service**[**https://www.ncjrs.gov/teendatingviolence/publications.html**](https://www.ncjrs.gov/teendatingviolence/publications.html) **Searched 26/04/2021**
Browsed teen dating violence section.
2 records selected to screen.
 **Crime Solutions**[**https://www.crimesolutions.gov/TopicDetails.aspx?ID=403**](https://www.crimesolutions.gov/TopicDetails.aspx?ID=403) **Searched 26/04/2021**
Programs filtered by Setting (Deliver): School; by Topic: Victims & Victimization. 0 selected to screen from 27 entries.
 **World Health Organization
http:///www.who.int
Searched 27/05/2021**
Browsed publications:

Health topics > Violence against women
Health topics > Child maltreatment
Health topics > Violence
Health topics > Violence against women
Health topics > Violence against children
Health topics > Youth violence
Health topics > Intimate partner and sexual violence
0 records selected to screen

**AVA Against Violence and Abuse**[**https://avaproject.org.uk/**](https://avaproject.org.uk/) **Searched: 26/04/2021**
Browsed Evaluations and Reports. 1 record to screen from evaluations

**RAND**[**http://rand.org**](http://rand.org) **Searched: 26/01/2021**
Browsed: Topics > Violence > Dating Violence; Topics > Violence > Domestic Violence; Topics > Violence > Sexual assault.
2 selected to screen from dating violence.

**Sexual Violence Research Initiative**[**https://www.svri.org/documents/prevention-research-and-programmes**](https://www.svri.org/documents/prevention-research-and-programmes) **Searched: 27/5/2021**
0 records selected to screen

### **ClinicalTrials.gov**

**Searched 7/07/2021**

| **Search** | **Number of records** |
| --- | --- |
| Condition/disease: dating violence; Other terms: school | 14 |
| Condition/disease: dating abuse; Other terms: school | 0 |
| Condition/disease: relationship violence; Other terms: school | 26 |
| Condition/disease: intimate partner violence; Other terms: school | 23 |
| Condition/disease: domestic violence; Other terms: school | 15 |
| Condition/disease: gender based violence; Other terms: school | 10 |
| Condition/disease: gender violence; Other terms: school | 13 |
| Condition/disease: sexual harassment; Other terms: school | 5 |
| Condition/disease: sexual assault; other terms: school | 11 |
| Condition/disease: rape and sexual assault; other terms: school | 3 |
| Condition/disease: “relationship abuse”; other terms: school | 2 |
| Condition/disease: Bullying; other terms: homophobic AND school | 2 |
| Condition/disease: Bullying; other terms: transphobic AND school | 2 |
| Condition: violence against girls; other terms: school | 16 |
| Condition: violence against women; other terms: school | 3 |
| Condition: healthy relationships; other terms: school | 7 |
| Condition: “sexual violence”; other terms: school | 16 |

### **WHO ICTRP**

**Searched 7/07/2021**

| **Search term** | **Number of records** |
| --- | --- |
| Dating violence AND school | 4 |
| Dating abuse AND school | 2 |
| Relationship violence AND school | 6 |
| Intimate partner violence AND school | 10 |
| Domestic violence AND school | 9 |
| Gender based violence AND school | 9 |
| Gender violence AND school | 11 |
| Sexual harassment AND school | 6 |
| Sexual assault AND school | 3 |
| Rape AND school | 2 |
| Relationship abuse AND school | 3 |
| Homophobic bullying AND school | 0 |
| Transphobic bullying AND school | 0 |
| Violence against girls AND school | 1 |
| “Healthy relationships” AND school | 6 |
| Sexual violence AND school | 19 |

### **Experts contacted**

| 1 | Dr Bruce Taylor | University of Chicago |
| --- | --- | --- |
| 2 | Dr Sidnei Rinaldo Priolo Filho | Universidade Tuiuti do Paraná (UTP) |
| 3 | Professor Pat Mahony | King’s College, London |
| 4 | Dr Christoph Muck | University of Munster |
| 5 | Dr Jo Bell | University of Hull |
| 6 | Dr Paul Schewe | University of Illinois, Chicago |
| 7 | Associate Professor Katie Edwards | University of Nebraska-Lincoln |
| 8 | Professor Patricia Kerig | University of Utah |
| 9 | Professor Peter Jaffe | Western University |
| 10 | Professor Daniel Whitaker | Georgia State University |
| 11 | Dr Anastasia J Gage | Tulane University |
| 12 | Dr Claire Fox | Manchester Metropolitan University |
| 13 | Kristin Ward | Clarus Research |
| 14 | Professor Nicky Stanley | University of Central Lancashire |
