# Supplementary material for: School‐based interventions for preventing dating and relationship violence and gender‐based violence: A systematic review and synthesis of theories of change
Source: Rev Educ. 2022 Dec 15;10(3):e3382. doi: 10.1002/rev3.3382 (PMC10116865; doi:10.1002/rev3.3382)
Supplement: Supplementary file 2 — Appendix S2 [file REV3-10-0-s003.docx]

**Supplementary Material 2**

**Table 1 Summary of intervention theories of change**

| **Intervention** | **Studies (Author, Year, Country)** | **Summary of theory of change with description of intervention** | **Existing theories drawn on** |
| --- | --- | --- | --- |
| 5W’s Bullying Intervention (GBV) | Merrell (2004)  USA | The ‘5 W’s’ intervention is a drama-based social skills program, targeting the bystander in the bully and victim relationship. It aims to teach the bystander how to effectively interact with school officials, victims and bullies in the school environment to help break the ‘code of silence’ that surrounds school bullying.  The intervention focuses on the who, what, where, when and why of bullying. Five scenarios are designed to answer the questions: Who to report bullying to; Why report bullying; What to report; Where to report; and When to report bullying. Role-playing as a form of drama, is used to teach social skills, giving students the opportunity to practise and observe such skills, and reflective write-ups enables students to reflect on the actions of the characters and assess their feelings and attitudes. The author suggests that the mechanisms of change are the peer interaction and the opportunity to practise skills. Other suggestions include the development of empathy and conflict resolution skills.  Ultimately, the intervention aims to ensure that students develop effective social skills and feel empowered to communicate effectively and provide support for others. | Social competence (e.g. Schneider, 1993); Olweus (1993) bully prevention and intervention model |
| Acquaintance Rape Education Program (GBV & DRV) | Fay & Medway (2006)  USA | This intervention is a school-based acquaintance rape prevention program that aims to reduce students’ acceptance of rape myths and dating violence. It comprises six activities that are modified from the original Parrot program (1991): (i) assertive behaviour; (ii) sexual pressure and mixed messages; (iii) communication, gender expectations and dating and drinking; (iv) rape myths, victim blaming and sexual violence in the media; and (v, vi) date rape definition and problem solving, rape prevention and what to do if rape occurs. The activities in the original program were designed to encourage critical thinking, reflection and discussion, and based on extensive pretesting, the activities selected for this modified program are practical and aim to elicit student participation. The activities help students assess their own risk of acquaintance rape, teach specific prevention strategies and inform about community counselling and support services.  The two hour intervention, delivered over two days, was relatively intensive and was fully integrated into the school curriculum. Each session was taught by a male and female facilitator whose age was within 10 years of the targeted students. They modelled appropriate communication and used active teaching techniques to promote discussion.  The primary mechanism of change appears to be in using the interactive learning activities to increase awareness of dating violence and prevention strategies, encourage student self-exploration and increase communication. | None mentioned |
| Adolescent Rape Prevention Program (GBV) | Kershner (1995)  USA | This intervention is a one-week (five sessions) stranger and acquaintance rape prevention program. The mechanisms of change are increased knowledge, and changed attitudes, about rape. The format of the program comprises a socio-cultural model and a prevention/safety component. The societal issues informing the program includes (i) gender-role socialisation patterns; (ii) conservatively held beliefs that tend to blame the victim; (iii) social-sexual interaction relative to dating. Through examining the causal factors that contribute to rape, students learn that rape is an unacceptable crime of violence. The students learn to identify potentially dangerous situations that may be a prelude to rape and assertiveness skills to help decrease the risk of rape. The program uses lectures, films, class discussion/participation, and games, with opportunities for discussion about gender stereotypes and dating issues. | Burkhart & Fromuth (1991) |
| *Battered Women: Violence Behind Closed Doors* (GBV) | Walther (1986)  USA | This intervention uses a film which contains some of the myths surrounding wife beating and information on wife abuse. It aims to change attitudes toward wife abuse. The primary mechanism of change seems to be that knowledge on the extent of wife abuse would change attitudes to wife abuse and its justification. | None mentioned |
| *Benzies & Batchies* (GBV) | de Lijster *et al.* (2016)  Netherlands | This interactive school-based intervention aims to prevent male and female adolescent sexual harassment behaviour, both in victims and perpetrators, in secondary school students by combining a play with skills lessons and peer education. There are four elements to the intervention; (i) an introductory lesson, an educational peer-performed play followed by a peer-led discussion, (iii) three classroom lessons to teach skills and resilience regarding social and sexual behaviour, and (iv) a closing lesson. The play comprises short scenes in which male and female peer educators performed examples of sexual harassment (both victimisation and perpetration) and reactions to them. The play lasts 30 minutes and is followed by a 60 minute discussion. The introductory and closing lessons are given in the classroom by the students’ teacher and the three lessons on student skills and resilience are delivered by experienced and trained social skills instructors from outside the school.  The theory of change focuses on the five determinants of sexual harassment behaviour: attitude, perceived social norms, self-efficacy, intention and prototype. It is underpinned by the understanding that peer-educators can use modelling to influence students’ perceptions of other people’s behaviour (social normative behaviour). To change the five behavioural determinants, modelling, planning coping responses, resistance to social pressure and guided practice are used during the three skills lessons. Peer-led group discussion, worksheets, films and role-play supported the interactive and skills lessons of the program. | Theory of Planned Behaviour (Ajzen, 1991), Reasoned Action Approach (Fishbein & Ajzen, 2010), and Prototype Willingness Model (Gerrard *et al.*, 2008). Social learning theory (Bandura, 1986). |
| BITB-HSC - Bringing in the Bystander – High School Curriculum (GBV & DRV) | Edwards *et al.* (2019)  USA | BITB-HSC is bystander-focused, classroom-delivered curriculum intervention that aims to reduce rates of interpersonal violence among high school students. It is a seven session curriculum delivered to a mixed sex audience and co-delivered by one facilitator who identifies as male and one who identifies as female. Modules 1-3 educate students about stalking, sexual harassment, sexual assault, dating violence and how these behaviours negatively impact communities. Modules 4-5 introduces a bystander framework, emphasises participants’ roles in creating a healthy community, and teaches participants how to recognise interpersonal violence. Modules 6-7 teaches students how to intervene safely and effectively.  The mechanisms of change are through increasing knowledge and decreasing myths of interpersonal violence, and developing skills on how to safely and effectively intervene before, during and after situations of relationship abuse and sexual assault, as well as supporting victims in the aftermath of these experiences. Lectures, large and small group discussions, hands-on and experiential exercises and skills-building activities are used.  Beyond the knowledge and practical skills that the classroom curriculum aims to develop, there is a 60 minute School Personnel Workshop that trains teachers and other school staff to be positive bystanders in situations of adolescent inter-personal violence. This mechanism of change works on school personnel modelling bystander behaviour and reinforcing the information and skills taught in the curriculum. BITB-HSC aims to train **all** members of a community to play a role in ending relationship abuse and sexual assault. | Health Belief Model (Rosenstock, 1974), Transtheoretical Model of Change (Prochaska & DiClemente, 1984), Theory of Planned Behavior (Ajzen, 1991), Diffusion of Innovation Theory (Rogers, 2002) |
| Brief intervention based on an Incremental Theory of Personality (DRV) | Fernández-González & Sánchez-Álvarez (2020)  Spain | This brief single-session (one hour) intervention is based on implicit theories of personality (ITP) to decrease dating violence perpetration and victimisation. The intervention sits within the context of brief interventions based on ITP in emotional wellbeing of adolescents and young people (such as depression and peer conflict relationships). ITP interventions aim at changing entity theories of personality (i.e. the belief that personal characteristics are fixed and cannot be changed) for an incremental theory of personality (i.e. the belief that people do have potential to change). ITP interventions use strategies derived from research on persuasion and attitude change, such as a focus on students’ perspectives and scientific information. The intervention’s intended mechanism of change lies in the development of prosocial behaviours, increasing empathy, promoting self-esteem and self-confidence to handle stressful situations, and decreasing hostility and angry feelings. The authors argue that strategies aimed at preventing peer conflict may also prevent dating aggression.  The intervention has three main parts that are presented as a writing assignment to be completed by students in about 50-60 minutes. 1. Students read scientific evidence that individuals have potential to change; they read studies showing that behaviours are controlled by ‘thoughts and feelings in brains’ and that pathways in the brain have potential to be changed. Students are then asked to write three sentences in their own words, explaining why scientific evidence shows it is true that people have potential to change. 2. Students read several normative quotes purportedly written by other students who had previously read the scientific information and endorsed its conclusions (‘descriptive norms’). 3. Students write their own version of such a narrative to share with future students: they have to imagine a time when they felt withdrawn, rejected or disappointed by another person at school; and they then have to imagine that the same event has happened to another student and write one to three paragraphs describing what s/he can say to help the student understand that people can change and that the things that are happening to the student can also change. The aim of the activity is to facilitate the internalisation of the ITP message. |  |
| Bystander intervention (DRV) | dos Santos *et al.* (2019)  Brazil | This peer- and bystander approach-based intervention aims to improve the intent to offer help, empathy and bystander attitude. It is delivered in three weekly sessions of 90 minutes and focuses on healthy vs unhealthy relationships, quality of friendships in the peer network and role of bystanders. The sessions comprises five steps; homework discussion (sessions 2 & 3), the day’s thematic approach, activity discussion, homework (sessions 1 & 2) and session evaluation. Each participant receives an intervention support guide. The activities includes a comic book story, mapping the network of close friendships, and video debate about bystander approach encourages engagement and the exercise of empathy. The intervention’s short-term outcomes are considered to be precursors to medium-term (help-seeking and help-offering between friends in dating violence situations and friendship quality) and long-term (number of friends who are perpetrators and victims of dating violence, and victimisation and perpetration of dating violence) outcomes.  The assumptions underpinning the theory of change are that as friends play a crucial role in the emergence, development and maintenance in young people’s dating relationships, teaching them to help their friends by competently intervening when they witness dating violence is a promising preventive goal. The mechanisms of action are raising awareness of dating violence, boosting the modelling of helping behaviour (seeking and offering help) within the friendship network, and developing empathy skills to increase empathetic communication and ability to see the other’s perspective. | Bioecological model: Bronfenbrenner (1996); Bronfenbrenner (2004); Bronfenbrenner & Evans (2000); Poletto & Koller (2008); Cognitive Social Theory Bandura (1986), Social Network Theory (Sluzki, 1997), Bystander Intervention Model (Latané & Darley, 1970) |
| Bystander intervention curriculum (GBV) | Lee *et al.* (2018)  Taiwan | This bystander intervention course aims to strengthen high school students’ ability to improve protection and help victims when they witness sexual harassment or bullying incidents. It is modelled on the Take Care online bystander intervention program (Kleinsasser et al, 2015). The curriculum comprises three stages and six units. The curriculum content includes understanding the concept of sexual harassment, intimacy and boundaries, responsibility of bystanders and bystander intervention strategies. The mechanisms of action are that by increasing knowledge and understanding of sexual harassment, and by learning effective intervention strategies, students will be able to act as bystanders and intervene in sexual harassment incidents. | None mentioned |
| CD-ROM Educational Program on Sexual Knowledge (GBV) | Yom & Lee (2005)  South Korea | This intervention aims to improve students’ knowledge and attitudes towards sexual violence, decrease recurring sexual violence, and enhance the coping skills of the victims of sexual violence. It is delivered as part of regular classes by the school nurse for one hour.  The CD-ROM has four primary menu selections or domains and each domain divides into sub-menus. 1. Understanding sexual harassment and violence which deals with definition, types, causes, misconceptions and complications. 2. Cases of sexual harassment and violence: this domain has nine videos. 3. Prevention of sexual violence and coping with the situation which deals with prevention, action and follow-up. 4. General information about sex which deals with physiology, expression, role, environment and equality. Each domain has objectives and three of the domains have quizzes and feedback is given for correct and incorrect answers. The authors indicate that the CD-ROM capitalises on the possibilities of multimedia using text, hypertext, graphics, cartoon, sound and video and is engaging for students. It is ‘familiar, accessible and friendly’ and enables the student or learner to be the lead actor in the learning process.  The primary mechanism of action seems to be that increasing knowledge about sex and sexual violence reduces the rate of occurrence of sexual violence. | None mentioned |
| Coaching Boys Into Men (CBIM) (DRV) | Miller *et al.* (2012)  Miller *et al.* (2013)  Miller *et al.* (2020)  USA | This intervention is an athletic coach-delivered dating violence (DV) perpetration prevention program for both high and middle school male students. It builds upon social norms theory to increase bystander behaviour related to DV prevention. It posits that DV perpetration often emerges in the context of male peers and therefore, prevention requires addressing perpetrator attitudes and behaviours. The assumption is that because athletes demonstrate great leadership abilities, the intervention with them may diffuse through the student population, and that coaches are a ‘natural ally’ to the intervention, in that they have an influential, non-parental role model to positively impact student thinking and behaviour. Coaches act as positive role models and lead brief weekly discussions with athletes about DV prevention during the sporting season to change athletes’ attitudes and behaviours. It is theorised that athletes will have increased awareness and recognition of abusive behaviours, increased positive gender-equitable attitudes, and demonstrate intention to intervene as bystanders with peers. Another possible mechanism of action is increasing student self-efficacy to intervene. | Social norms theory (Berkowitz, 2002) |
| ‘Dat-e Adolescence’ (DRV & GRV) | Munoz-Fernandez *et al.* (2019)  Sanchez-Jimenez *et al.* (2018)  Spain | This intervention is a multicomponent prevention program for the reduction of dating aggression and victimisation and bullying in adolescents. It uses the Dynamic Developmental Systems Model (DDSM) as a theoretical framework; the DDSM acknowledges that dating violence is associated with multiple risk factors which are addressed in Dat-e-Adolescence. The authors theorise that if adolescents reflect upon violence, gender norms, how the peer network influences their behaviour, and they develop socio-emotional skills to be confident within their dating relationships, they will be capable of building healthy relationships free of dating violence and gender-based violence.  The intervention comprises seven one-hour sessions: the first five are led by researchers and the final two are led by peers. They aim to increase awareness about healthy behaviours in relationships, encourage emotional regulation, promote self-esteem, improve communication skills, promote coping and conflict-resolution strategies and increase awareness of bystander influences. Interactive and experiential learning techniques are used such as discussions, debates, role-playing and decision-making games. | Dynamic Developmental Systems Model (Capaldi *et al.*, 2005) |
| Dating and Sexual Responsibility Curriculum (DRV) | Pacifici *et al.* (2001)  USA | This intervention is a psycho-educational intervention for high school teenagers on preventing sexual coercive behaviour in dating situations. The underpinning assumption is that young people are not aware of how their attitudes contribute to coercive behaviour.  The multimedia curriculum is organised into three 80-minute periods of class instruction and an additional period in which students individually viewed an interactive video, *Virtual Date*. The curriculum focuses on increasing awareness of sexual coercion, understanding the underlying thoughts and feelings that contribute to coercive behaviour, and building positive social skills.  The curriculum is participatory and class activities integrate the use of video, role play and discussion. The video material is designed to be credible for students by depicting peer discussion groups, dramatized stories and a series of brief dating scenarios to identify and analyse behaviour. The *Virtual Date* is an interactive video story with two versions, one told from the male perspective and the other told from a female perspective. Varying levels of coercion are depicted in the story, depending on choices made by the viewer. At the end of the story, the student is presented with an educational discussion by a peer that reflects on the choices that s/he made on the date. In addition to the knowledge and skills that the curriculum aims to develop, one of the intended mechanisms of action is the modelling of more appropriate behaviour (i.e. less coercive behaviour) in the video and interactive activities. | None mentioned |
| Dating Matters (DRV) | Niolon *et al.* (2019)  DeGue *et al.* (2021)  USA | Dating Matters is a comprehensive, multi-component prevention intervention with components at multiple levels of the social ecology to promote healthy relationships and prevent teen dating violence. This is predicated on the argument that in other areas of violence prevention, comprehensive approaches are more effective than single-component approaches. This intervention, delivered over four years, aims to create a ‘surround sound’ effect promoting healthy relationship behaviours and preventing unhealthy ones at the individual, family, neighbourhood and community levels of the social ecology.  Students (sixth to eighth graders) receive a program specific to their grade that teaches them about healthy relationships and assists in practising healthy relationship skills. The parent programs, specific to student grade, teaches skills for positive parenting and how to communicate effectively with their children on healthy relationships. All teachers complete online educator training providing information and resources on teen dating violence. The youth communications program reinforced community activities and messages about healthy relationships with community activities and other resources. Local health departments implementing the intervention are assisted in assessing and building capacity for comprehensive teen dating violence prevention and tracking local policy and indicator data relating to teen dating violence. | None mentioned |
| Dating Violence Prevention Program (DRV)  USA | Avery-Leaf *et al.* (1997)  USA | This intervention aims to change high school students’ attitudes that justify the use of dating aggression. The theory of change is based on the assumption that aggression is a multi-determined phenomenon i.e. both social and psychological. The importance of patriarchal social structure in the etiology of male violence towards female partners is mentioned.  The five-session curriculum provides a didactic, skills-based approach focusing on attitude change and skills enhancement. Its objectives are to (i) promote equity in dating relationships; (ii) challenge individual and societal attitudes towards violence as a means of conflict resolution; (ii) identify constructive communication skills (focusing on negotiation and conflict resolution); and (iv) support resources for victims of aggression. Help-seeking for those involved in aggressive relationships is also covered. | Feminism: Dobash & Dobash (1979); Yllo (1993) |
| Dating violence Prevention Program (DRV) | Filho (2017)  Brazil | This DRV intervention (and pilot) aims to prevent dating violence (as well as alcohol consumption) and increase problem-solving behaviour. It has 10 sessions of 50 minutes delivered three times a week during class. The curriculum covers types of violence, positive and negative aspects of relationships, gender roles, peaceful conflict resolution, sexuality and sexual violence, and seeking help in violent situations. The sessions were interactive with for example, role-playing activities to show non-violent solutions to conflict. The mechanisms of action are the knowledge and techniques/strategies for problem-solving and conflict resolution that the curriculum aims to develop. | None mentioned |
| Dating Violence Prevention Program (DRV) | Macgowan (1997)  USA | This dating violence program aims to change knowledge, attitudes and preventative strategies for dealing with relationship violence. It is designed to help students recognise dating violence, understand its causes, and make decisions to avoid or end an abusive relationship. The program comprises five one-hour sessions delivered over five days.  The mechanisms of change are that increasing awareness of violent and abusive relationships, and understanding attitudes towards violent behaviour (such as possessiveness and jealousy), and developing communication and problem-solving skills through teacher-student discussions and experiential exercises, would prevent dating violence. | None mentioned |
| Developmental Guidance Unit & Self-instruction Module about Sexual Harassment (GBV) | Sabella (1995)  USA | This intervention is a developmental guidance unit and self-instruction module about sexual harassment for adolescents. It aims to enhance knowledge, attitudes and behaviour of students, targeting the risk factors for sexual harassment such as socio-cultural norms, interpersonal communication behaviour, sex role stereotyping and lack of victim reporting. The theoretical basis for the study is developmental guidance which assumes that human nature moves individuals sequentially and positively towards self-enhancement. Development guidance units are designed to facilitate student learning and achieve a number of goals which include understanding the school environment, self and others, attitudes and behaviours, decision making and problem solving and interpersonal communication skills.  The intervention is delivered in one of three ways; adult-helper led, peer-helper led and self-led. The self-instruction module requires students to read and respond to materials on sexual harassment issues used in the adult-led and peer-led sessions (six sessions) but without group discussion and interaction. | Myrick (1993) |
| ECPVG - Educación Comunitaria para la Prevención de la  Violencia de Género  (Community Education for the Prevention of Gender Violence Program) (GBV) | Bando *et al.* (2019)  Bando *et al.* (2018)  El Salvador | ECPVG is an adaptation of Progam H and M aiming to address GBV through education and provision of life skills. It aims to change students’ knowledge, coping skills and attitudes towards gender norms. The program comprises two components: (i) educational groups where students reflected critically and discussed gender equality; (ii) support for students to create and execute a social marketing campaign to promote gender equality for one month in the school. The activities associated with this include workshops, puppet shows, skits and art projects, as well as the dissemination of key messages through promotional materials such as posters.  The logic of the program is that if students learn and discuss gender roles, they will improve life skills and change their attitudes towards gender discrimination. As a result, students will change the dynamics in their intimate relations. The rationale is that teachers discuss gender bias in the classroom, then students may talk among themselves about gender bias outside the classroom. If they talk more about gender bias outside the classroom, then they may change their perceptions about what the group norm is. Students who discuss gender with their peers may shift attitudes and norms toward more equitable ones to fit in with the group. A change in students’ attitudes and skills to deal with conflict may lead to a reduction in GBV.  The key pathway to change to change social norms is through social interactions. The group sessions aim to dialog and social interactions as a way of learning. In designing and leading the campaign, students use their own words and language to promote change in their schools. | Social norms theory (Scott & Marshall, 2009) |
| Ending Violence: A Curriculum for Educating Teens on Domestic Violence and the Law (DRV) | Jaycox *et al.* (2006a)  Jaycox *et al.* (2006b)  USA | Ending Violence is a brief three-class prevention program that teaches young people about domestic violence, healthy relationships and legal rights of victims and legal responsibilities of perpetrators. The theory of change is consistent with social learning theory (Bandura, 1977) and aims to reverse acceptance of violence by focusing on how partner violence is illegal and to increase knowledge and help-seeking by providing information and resources. It is informed by the assumption that the cultural norms to which Latino students are exposed may be more tolerant of partner violence and that knowledge of US law is limited. It is taught by bilingual, bicultural attorneys to emphasise the legal dimension and to increase students’ comfort with speaking to attorneys (and to inform about legal services). The structured program appears to have some interactive learning with discussion, exercises, a video and a game, and a mock hearing (role-play) for a restraining order. | Social learning theory (Bandura & Walters, 1977) |
| Expect Respect (GBV) | Rosenbluth *et al.* (2004)  Whitaker *et al.* (2003)  Rosenbluth *et al.* (2011)  USA | Expect Respect Elementary School Project aims to prevent bullying and sexual harassment. The theoretical foundation for Expect Respect is based on the multi-level, multi-component, school-based prevention program developed by Olweus et al (1999). Similar to Olweus et al’s program, Expect Respect aims to take a ‘whole-school’ approach to preventing bullying and sexual harassment by preparing all members of the school community to recognise and respond effectively to bullying and sexual harassment. The intervention has five components: a 12-week classroom curriculum; staff training; education and support for parents; guidance for administrators for policy development, and support services for students who have been affected by bullying, sexual harassment, or sexual or domestic violence. Lesson included writing assignments, role-plays of how to intervene upon witnessing bullying, class discussions, and opportunities for students to practise intervention skills in the classroom. | Olweus Bully Prevention Program (Olweus *et al.*, 1999) |
| Expect Respect: Preventing Teen Dating Violence (DRV) | Roberts (2009)  USA | This intervention aims to change teen’s attitudes towards abusive dating behaviours and reduce teen dating violence with the ultimate goal of reducing the incidence of domestic violence in adulthood. It is designed for teens in abusive relationships to recognise them as abusive and take measures to leave the relationship safely; and for those at the beginning of a relationship, to recognise signs of abuse and end the relationship. The program uses the theory of the cycle of violence (Walker, 1979) to suggest that teen dating violence is a precursor to domestic violence, and that gaining knowledge to better recognise abusive relationships during high school helps to avoid the consequences of domestic violence in the future as adults.  It is a four-session program and includes elements such as the cycle of violence, issues of power and control, signs of abuse, how to leave an abusive relationship safely, and how to establish and maintain healthy relationships. The program has discussion, videos, worksheets and a ‘case study’ of a survivor of teen dating violence and domestic violence who shares her experience of violence with the students. | Cycle of violence (Walker, 1979) |
| Fourth R:  Skills for Youth Relationships (DRV & GBV) | Cissner & Ayoub (2014)  Wolfe *et al.* (2009)  USA | The Fourth R: Skills for Youth Relationships is an intervention that integrates dating violence prevention with lessons on healthy relationships, sexual health and substance use. The curriculum comprises three units of seven 75-minute classes each; (i) personal safety and injury prevention, (ii) healthy growth and sexuality, and (iii) substance use and abuse. In the Bronx adaptation of Fourth R the curriculum comprises 26 50-minute sessions. The lessons are integrated into existing core curricula with teachers building on Reading, ‘Riting and ‘Rithmatic by incorporating relationship education into physical education, health, or English classes.  The Fourth R draws from social learning theory and how young people learn to formulate and choose their behaviour strategies for decision making, conflict resolution and self-efficacy. The underpinning assumption is that these strategies, along with strategies for promoting wellbeing and resilience for related risk behaviours of substance use and unsafe sex, may well reduce physical dating violence. The curriculum is interactive aiming to engage students through a variety of exercises and activities and there is ‘extensive skill development’ to enable young people to develop strategies for dealing with pressures and resolving conflict without resorting to violence. This is achieved through the use of scenarios and role-playing. Diffusion of the Fourth R curriculum messages across the schools via peer-to-peer contact and or teacher-student contact is also a feature of the Bronx adaptation. | Social learning theory (Cissner & Ayoub, 2014) and stages of social development (Cissner & Ayoub, 2014) |
| Gender Equity Movement in Schools (GEMS) (GBV) | Achyut *et al.* (2011)  Achyut *et al.* (2016)  Achyut *et al.* (2015)  ICRW (2017)  India | The Gender Equity Movement in Schools (GEMS) is a two-year violence prevention program comprising Group Education Activities (GEAs) and a school-based campaign for both boys and girls in India. The theory of change draws from the social normative framework (Cardoso & Oliveira, 2011) and is informed by the knowledge that violence among boys and girls in India are ‘deeply rooted’ and normalised, and that aggressive behaviour particularly among boys are often tolerated or ignored as they are considered natural. GEMS uses the cognitive-affective approach and life-skills to bring about change, engaging students to recognise, challenge and transform gender norms.  GEMS aims to promote gender equality by examining social norms that define men and women’s roles and questions the use of violence. The GEA component covers three themes – gender, the body and violence - in the first year, and in the second, focuses on building skills to respond positively to discrimination and violence. The school campaign is a week-long series of events designed in consultation with students and involved games, competitions, debates and short plays. The primary mechanism of change appears to be the use of participatory methods such as role-plays, games and debates to engage students in ‘meaningful and relevant interactions and reflection’. The GEMS Diary is a workbook that also facilitates student reflection on gender roles, relationships and violence. | Social normative framework (Cardoso & Oliveira, 2011) |
| Good School Toolkit (GBV) | Devries *et al.* (2017)  Uganda | This complex intervention aims to change the operational culture of the school with the view to reducing emotional, physical and sexual violence from staff to students and between peers. The whole school approach to violence prevention is about promoting respect, participation and reflection on concepts and exercise of power. The Toolkit comprises six steps designed to be implemented in sequence, drawing on the Transtheoretical Model of behaviour change (Prochaska & Velicer, 1997). The steps contain more than 60 activities which are informed by a number of behaviour change technique, such as setting goals, making action plans, and rewards and reinforcement.  The activities focused around improving the school compound and creating a better learning environment, respect and better understanding of power relationships, improving teaching techniques and improving accountability, and learning non-violent methods of discipline. | Transtheoretical Model of Behavior Change (Prochaska & Velicer, 1997) |
| Green Dot (DRV & GBV) | Coker *et al.* (2017)  Coker *et al.* (2019)  Coker *et al.* (2020b)  Coker *et al.* (2020a)  Coker *et al.* (2021)  USA | This programme aims to engage potential bystanders to act to reduce sexual violence (and related forms of interpersonal violence) and acceptance of sexual violence. Green Dot is theory-based and draws on bystander psychology, diffusion of innovation theory and sexual violence perpetrator characteristics. It provides training for young people to recognise situations and behaviours that can contribute to violence and determine actions that they can take to reduce the likelihood or effect of violence. Active bystander behaviours are called ‘green dots’ to distinguish them from ‘red dots’ or behaviours that may contribute to violence. The curriculum comprises school wide presentations to orient students to their potential role as engaged bystanders and explains how to recognise ‘red dots’ and ‘green dots. The second part of the intervention uses the popular opinion leader (POL) strategy which suggests that training 12-15% of a student body would maximise diffusion of the intervention. Students identified as leaders participated in a 5-hour bystander training session. Training focuses on violence victimisation, perpetration and on prosocial behaviours to recognise situations that may lead to violence and to act directly to distract or to delegate tasks to reduce the likelihood of violence. Training also focuses on sexual harassment, stalking and partner violence. A key mechanism of change is the role of student leaders in diffusing prosocial behaviour and norms to other students through peer networks which is expected to contribute to the normalisation of prosocial and bystander behaviours in the school. | Bystander psychology (Bryan & Test, 1967; Latané & Darley, 1970; Clark & Word, 1974; Chekroun & Brauer, 2002), diffusion of innovation theory (Darley & Latane, 1968; Rushton & Campbell, 1977) and sexual violence perpetrator characteristics (Lisak & Roth, 1988; Lisak & Miller, 2002; Johnson *et al.*, 2006) |
| Health belief model educational program (GBV) | Garmaroudi *et al.* (2016)  Iran | This intervention is an educational program informed by the Health Belief Model and aims to prevent domestic violence behaviours in high school female students. The curriculum content of the program addresses defining violence and types of violence, the physical and psychological effect of domestic violence, anger symptoms, and prevention and anger management. It is delivered in three 45 minute sessions during one month. Interactive learning is a feature of the delivery with question/answer sessions, group discussions and brainstorming. The intervention’s theory of change uses the Health Belief Model in terms of increasing awareness of negative consequences of violent behaviours and benefits of engaging in non-violent behaviours. | Health Belief Model (Davidson & Morrison, 1983; Ajzen & Madden, 1986) |
| IMPower (GBV) | Decker *et al.* (2018)  Baiocchi *et al.* (2017)  Malawi, Kenya | IMPower is a standardised six-week empowerment self-defence (ESD) intervention used for sexual assault risk reduction among girls in Malawi and Kenya. The principle underpinning the intervention’s theory of change is that it responds to the structural forces, including social norms that tolerate sexual violence and expect silence in response particularly from young women, in settings where sexual violence is endemic. Structural forces disempower young women and ‘blame them for their victimisation’. The mechanisms of action are increases in self-defence knowledge and skills, confidence/self-efficacy and disclosure of violence.  IMPower is a 12 hour intervention, comprising two-hour sessions delivered over six weeks (booster training within three months in Kenya). It focuses on boundary recognition and boundary setting (e.g., name harmful behaviours, warn about consequences), negotiation and diffusion tactics, verbal assertiveness (e.g., yell if threatened), and physical defence skills, with the self-efficacy to implement these skills. The physical skills comprises closed target skills, weapons and targets. In recognition that self-defence can result in harm and injury, IMPower promotes boundary setting through voice and action, with physical self-defence as a last resort. A range of learning methods are used and include role-plays, facilitated discussions and verbal and physical skills practice. | Social learning theory (Bandura, 1977) and health belief model (Rosenstock *et al.*, 1988) |
| *It’s Your Game…Keep It Real (IYG)* (DRV) | Peskin *et al.* (2014)  USA | *It’s Your Game…Keep It Real (IYG)* is a two-year health education programme designed to delay sexual behaviour and promote heathy dating relationships in ethnic minority middle schools. It also aims to reduce dating violence behaviour. *IYG* is grounded in a skills-building approach based on social cognitive models of behaviour change.  *IYG* comprises a 24 lesson curriculum (12 lessons in 7^th^ grade and 12 lesson in 8^th^ grade) and its major focus is on developing the knowledge and skills for healthy relationships - the foundation for healthy adolescent sexual health. Topics covered in the 7^th^ grade curriculum include: identifying the characteristics of healthy and unhealthy friendships and dating relationships; skills training related to evaluating relationships, peer pressure and peer support; setting personal limits and recognising others’ limits; and recognising peer norms. These topics are reviewed in the 8^th^ grade. A wide range of learning strategies are used in the intervention and in addition to group-based activities in the classroom, there are computer-based activities which include interactive skills-training exercises, peer role model videos, quizzes, animations, fact sheets and ‘real world’ style adolescent serials. The intervention also provides individualised messages for students (e.g. personalised quizzes to assess whether students have healthy relationships).  The curriculum includes six parent-child homework activities which focuses on increasing communication regarding healthy friendships and dating relationships, using effective refusal skills, dating partners’ expectations, and parental rules about dating relationships. Here the intended mechanism of change is that improved communication between students and parents has the potential to reinforce the curriculum’s focus on healthy relationships. | Social cognitive theory (Bandura, 1986), social influence models (McGuire, 1972) and theory of triadic influence (Flay & Petraitis, 1994) |
| *Jesse* (GBV) | Boduszek *et al.* (2019)  Barbados | Jesse is a prosocial video game designed to tackle Intimate Partner Violence (IPV) accepting attitudes among children and adolescents in the Caribbean. Jesse’s theory of change draws from the General Learning Model (GLM) and hypothesises that students exposed to the misfortune of a woman experiencing physical and emotional abuse by a partner (as depicted in the video), will lead to increased cognitive and affective empathic reactions towards IPV victims. The game aims to affect players’ empathic responses to victims of domestic violence and increase awareness of the impact of IPV. It is designed to allow players to role-play different characters experiencing and/or perpetuating physical and emotional violence in a family context. | General Learning Model (GLM) (Buckley & Anderson, 2006) |
| Joven/Youth (Juntos Opuestos a la Violence Entre Novios/Together Against Dating Violence) (DRV & GBV) | Gonzalez-Guarda *et al.* (2015)  USA | Joven is a manualised, school-based dating violence prevention program, informed by ecodevelopmental theory (Pantin et al, 2009; Szapocznik & Coatsworth, 1999) and social cognitive theory (Bandura, 1977), and by input from dating violence service providers, community leaders, parents, students and school personnel. Ecodevelopmental theory builds and expands on Bronfenbrenner’s (1979, 1986) socioecological theory and guided the development of the intervention as it was consistent with the risk and protective factors identified by the Hispanic community as important to address dating violence prevention. Key to ecodevelopmental theory is that adolescents are in constant interactions with their peers, families, and schools (micro-system e.g. adolescent-parent interaction) which, in turn, are in constant interaction with one another (meso-system e.g. parent-school interaction). These systems are then influenced by external factors such as stressors and resources (exosystem e.g. related to immigration status and limited access to community resources), and the socio-cultural context (macro-system e.g. culturally ascribed gender norms). This guided the factors and levels of influence to target, and social cognitive theory (Bandura, 1977) guided the development of the activities that could contribute to behavioural change (e.g. skill building activities that could increase self-efficacy).  This intervention consists of six large group sessions for Hispanic adolescents, two for their parents, and two for school personnel. Each session includes psychoeducational and skill-building activities that are delivered using videos, music, group discussion and other modalities. In the first session, a TV sitcom about the acculturation differences between adolescents and adults are shown to highlight some of the differences in norms about relationships according to acculturation level and generation. In the second session, students use their phones to take pictures of images that represented healthy qualities of relationships and shared with these with one another to encourage discussion on healthy intimate relationships. In the fifth session, the students engage in a number of role-playing activities that provided them with opportunities to act out healthy bystander behaviours with friends who were in risky situations and at risk for physical or sexual harm. Parents join the adolescents in the final session to practice healthy communication skills in negotiating around curfews and dating | Ecodevelopmental theory (Szapocznik & Coatsworth, 1999; Pantin *et al.*, 2004) and social cognitive theory (Bandura & Walters, 1977) |
| Katie Brown Educational Program (DRV) | Joppa *et al.* (2016)  USA | This dating violence prevention programme is delivered in schools by a community-based organisation, Katie Brown Educational Program (KBEP). The KBEP prevention program is a brief, manualised, group-based curriculum that replaces five high school health class periods during one week. It is freestanding and can be incorporated into any existing high school health curriculum. The curriculum is based in social learning theory with lessons that aims to change cognitions (dating attitudes, expectations and knowledge) and behaviours (conflict resolution and communication skills) to help students foster healthy relationships.  Five sessions cover understanding violence; wants and needs in a relationship; expectations in dating relationships; communication skills; and cycles of violence and warning signs. Sessions include a lecture, discussion, group and individual activities, handouts and worksheets. Observational learning, role-play, and modelling of communication and healthy relationship skills are used; for example, students learn what assertive communication means, watch facilitators perform a role-play demonstrating assertive communication skills in a relationship context, and then practice their own assertive communication skills in a group game. | Social Learning Theory (Bandura & Walters, 1977) |
| Let Us Protect Our Future (GBV) | Jemmott *et al.* (2018)  South Africa | Let Us Protect Our Future is a theory-based, culturally appropriate, HIV risk-reduction intervention to reduce the experience and perpetration of forced sex among South African adolescents. It comprises 12 one-hour modules, with two modules delivered during each of the six sessions, on consecutive school days. These sessions include a number of activities which aim to develop the knowledge, skills and self-efficacy that adolescents need to avoid and decline forced sex. Activities focus on awareness of risky situations and how to plan to avoid them; the ‘Long Walk Home’ activity helps participants identify risky situations on their way to and from school and brainstorm strategies to reduce risk of sexual coercion. Other activities help participants to know and be able to express their limits to avoid risky behaviours, and develop their refusal skills.  It also aims to address gender issues and rape myth beliefs relevant to perpetration and experience of forced sex. A doll activity involving changeable clothing is used to challenge negative attitudes towards women and coercion, by enabling participants to consider whether a woman’s clothing is a legitimate reason to conclude about her character, and whether a girl dressed sexily is asking for sex and deserves to be forced to have sex. The intervention is implemented in mixed-sex groups and co-facilitated by a trained man and woman. An additional mechanism of action is that these facilitator pairs model egalitarian gender roles. | Social cognitive theory (Bandura, 1986) and theory of planned behaviour (Ajzen, 1991) |
| Me & You (DRV) | Peskin *et al.* (2019)  USA | Me & You is a multi-level technology-enhanced intervention to reduce dating violence (DV) perpetration and victimisation among ethnic-minority students. It is an adaptation of IYG (Peskin et al, 2014) which has two healthy relationship lessons. Me & You focuses exclusively on promoting healthy relationships and preventing DV. It aims to address the determinants of physical DV perpetration i.e. conflict resolution skills and norms towards violence, and all unhealthy relationship behaviour –emotional, physical, sexual and cyber.  The life-skills decision-making paradigm from IYG (select, detect, protect) is adapted and is central to the theory of change. The goal is to increase cognitive skills for decision-making in relationships, understanding the consequences of one’s actions, and solving problems (these skills underlie the socio-emotional learning framework). Students are instructed to select personal rules to have healthy friendships and dating relationships, to detect signs and situations that could challenge rules, and to protect their rules.  The intervention comprises 13 lessons (25 minutes each) – five classroom (including interactive role-plays, group discussion and other skills-building activities), five individual computer only and three classroom-computer blended (delivered in class with some group-based computer activities). Lessons cover modelling and skills practice for managing emotions and constructive communication skills. They emphasise that skills could be applied to any current of future relationship. The authors assert that technology facilitates tailored education, immediate feedback, simulated skills practice, greater enjoyment and interactivity. Computer activities include animations, peer video role modelling of skilled behaviours, interactive quizzes, and virtual role-play skills practice. Some activities are tailored to individuals e.g. quizzes about communication provide tailored feedback depending on users’ answers. The activities also challenge gender stereotypes by depicting both male and female as perpetrators and victims.  Me & You has two other components: a parent component of three parent-child take-home activities and two parent newsletters; and a school component of a two-day teacher training and one school newsletter emailed to all school staff. Take-home activities included interactive discussions to promote parent-child communication and connectedness, and teacher training focused on how to recognise DV, respond to students involved in DV and refer students to appropriate resources. | Social cognitive theories (Bandura, 1986; Montano & Kasprzyk, 2008), socioemotional learning (Weissberg *et al.*, 2015), and socioecological model (Sallis & Owen, 2002) |
| Media Aware  Media Aware - High School  (DRV & GBV) | Scull *et al.* (2018)  Scull *et al.* (2021)  USA | Media Aware is a 10 lesson, classroom-based media literacy education (MLE) program for improving adolescents’ sexual health outcomes. The intervention is teacher-led and in addition to a number of sexual health outcomes, aims to increase self-efficacy to refuse sex, decreased acceptance of strict gender roles and dating violence.  Media Aware – High School is a web-based program that aims to provide high school students with sexual health knowledge, media literacy skills and healthy decision-making skills regarding sexual activity and relationships. It comprises four highly interactive, self-paced modules, each designed to be completed within one class period.  The principle underpinning MLE is that it enhances critical thinking about media messages to disrupt the influence that inaccurate media messages may have on young people. The intervention uses the Message Interpretation Processing (MIP) Model (Austin & Johnson, 1997) which proposes that logical (perceived realism of, and perceived similarity to, media messages) and affective (perceived desirability of media messages) constructs influence the degree of identification with the media message and expectations in relation to the behaviour portrayed in the message. The model suggests that decreased perceived media realism, similarity and desirability can act as protective mechanisms that reduce the impact of media influence on unhealthy behaviour. The MIP model is consistent with TRA/TRB and its premise is that teaching critical thinking skills creates an ‘active filter’ through which images are processed. This is expected to change adolescents’ cognition about sexual practices and self-efficacy to engage in in healthy behaviours. These changes result in increased intentions for healthy behaviours, beliefs about normative sexual practices and less willingness for risk behaviours.  The mechanisms of action appear to be changes in students’ processing of media messages and media-skills - enhanced media deconstruction skills and increased media scepticism. | Theory of Reasoned Action (TRA) (Ajzen & Fishbein, 1980), Theory of Planned Behavior (TRB) (Ajzen, 1991), Message Interpretation Processing Model (Austin & Johnson, 1997) |
| My Voice, My Choice (MVMC) (GBV) | Rowe *et al.* (2015)  USA | My Voice, My Choice (MVMC) is a 90-minute assertive resistance training programme emphasising skills practice in an immersive virtual environment (IVE). It aims to reduce male-to-female sexual victimisation among adolescent girls. MVMC is designed to teach a specific set of self-protection skills (assertive resistance) and provide ‘multiple’ opportunities to practice skills in realistic environments. The theory of change is predicated on the assumption that teaching skills should be accompanied with opportunities to practice skills and receive feedback on use of skills. An additional assumption is that skills learned under conditions similar to those in which they are to be used are more likely to generalise to the real world. Simulation-based learning is used in the intervention as a mechanism for girls to learn and practise skills in situations that pose potential risk for sexual victimisation. The practice is conducted in an IVE in which the participant experiences herself as ‘in’ the coercive situation. The authors assert that the development of assertive resistance skills might have positive effects on reducing other forms of interpersonal victimisation.  Groups of two to four participants receive training from a female facilitator and practised skills in IVE simulations. The sessions begin with 30 minutes of discussion and modelling of assertive resistance skills. The female facilitator demonstrates assertive and non-assertive resistance in role-play interactions with a male actor. The facilitator then introduces the next part which was around 60 minutes. Each participant completes three virtual simulations in which the verbal sexual coercion increased in severity. The virtual environment for all simulations is a virtual bedroom, in which the participant is seated on a couch to the right of the male avatar. After each simulation the participant receives constructive feedback from the facilitator and other participants. Simulations could be 2-3 minutes long but could be repeated until the participant successfully demonstrates assertive resistance. At the end of the sessions, the facilitator leads with a concluding conversation about the experience and programme. | None mentioned |
| PP (Practitioner Program) & SPP (Scientist Practitioner Program) (DRV & GBV) | Muck *et al.* (2018b)  Muck *et al.* (2018a)  Germany | PP and SPP are two school-based universal sexual violence (SV) prevention programs for adolescents. These programs differ in their duration, active participation by students, and gender composition of audience, but not in content. Both use a fixed curriculum to deliver the same program topics in every class in the same way. The curriculum included the following content: (i) general knowledge about SV (definition of SV and general information); (ii) knowledge about professional help (local and online professional help services); (iii) victim blaming (victim-blaming attitudes and rape myths); (iv) personal space (perception of one’s own and others’ personal space). The PP curriculum is delivered in one 90-minute session via presentations, worksheets and quizzes. The SPP curriculum has an identical first session to the PP program and a second 90-muinute session where students are separated by gender and participate in more active learning via role-playing and group discussions. The mechanisms of action appears to be that active participation in learning will facilitate knowledge acquisition and attitude change. | None mentioned |
| Peer-to-peer sexual harassment intervention (GBV) | Durand (1997)  USA | This intervention aims to address peer-to-peer sexual harassment among junior high school students. The curriculum is designed to increase awareness of the behaviours that constitute peer-to-peer sexual harassment. It comprises three units on defining sexual harassment, causes of sexual harassment and how to prevent and stop sexual harassment. Within each unit there are various group activities, role-play, video, case studies and class discussion. The mechanisms of action are changes in knowledge, attitudes and beliefs about peer-to-peer sexual harassment. | None mentioned |
| Precede-Proceed Model based intervention (GBV) | Ekhtiari *et al.* (2013)  Ekhtiari *et al.* (2014)  Iran | This intervention is an educational and environmental intervention that aims to promote preventive behaviours of domestic violence amongst Iranian high school girls. Specifically, it aims to increase students’ awareness about domestic violence prevention, change students’ attitudes and behaviour, and promote reinforcing and enabling factors.  It follows the Precede-Proceed model, an eight stage model for planning, implementing and evaluating health promotion programmes. The intervention has a number of components: an educational programme with twice weekly, lectures; student focus group discussions on domestic violence issues; distribution of educational booklets to parents; training high school counsellors to effectively conduct domestic violence prevention education for students; and coordination with free counselling centres in selected districts and introducing them to the students. | Preceed-Proceed model (Gielen *et al.*, 2008) |
| PREPARE (DRV) | Mathews *et al.* (2016)  South Africa | PREPARE is a multi-component school-based HIV prevention intervention to delay sexual debut, increase condom use and decrease intimate partner violence (IPV) among adolescents. The program is built upon the Respect4U program (an intervention that was based on the Jewkes conceptual framework) and is informed by social cognition models including the Reasoned Action Framework (Fishbein & Ajzen, 2009) and the I-Change Theoretical Model (Elfeddali et al, 2012).  It comprises education sessions, a school health service and a sexual violence prevention programme. The education sessions included sessions on gender power inequities, relationships, sexual decision-making, IPV and sexual violence, and support for victims of IPV and sexual violence. The school safety programme focuses on reducing acceptability and prevalence of IPV and sexual violence in the school. The education methods are interactive and skills-based and included worksheets, group discussions, role-plays and games. The school safety progam comprises a photovoice activity (students take photographs of safe and unsafe places and situations) which aims to empower students to be the ‘driving force’ in improving the physical, emotional and sexual safety at school. It is also an opportunity for students to influence school safety policy. | Reasoned Action Framework (Fishbein & Ajzen, 2010) and I-Change theoretical model (Elfeddali *et al.*, 2012) |
| PREVIO (DRV) | Munoz-Rivas *et al.* (2019)  Spain | This psychoeducational intervention aims to change student aggressive verbal and physical behaviours to reduce the perpetration of verbal and physical violence. It comprises eight sessions (50-60 minutes) delivered weekly and focuses on providing knowledge on the nature, scope and impact of intimate partner violence (IPV), modifying student beliefs and attitudes to IPV and developing positive relationship skills. | None mentioned |
| PR:EPARe (Positive Relationships: Eliminating  Coercion and Pressure in Adolescent Relationships) (DRV) | Arnab *et al.* (2012)  Arnab *et al.* (2013)  Brown *et al.* (2012)  UK | This intervention is a digital game to raise awareness of, and promote discussion on, experiences of sexual coercion to improve the delivery of Relationships and Sex Education (RSE). The theoretical basis of the intervention is informed by Intervention Mapping which has six ‘steps’ or activities. The first step (needs assessment) identified determinants including attitude, knowledge, self-efficacy or skill and subjective norms about the risk of experiencing or perpetrating coercive behaviours. The game aims to reduce the likelihood of being coercive towards others or allowing others to successfully coerce by targeting these determinants as they align to behaviour.  PR:EPARe is scenario-based game with two parts: (i) focuses on developing knowledge and understanding risk; and (ii) immersive scenarios to address more complex psychological determinants such as attitude and self-efficacy. A key target is for students to be able to identify the nature and levels of coercion (embedded in the game’s narrative content) and how to respond from a range of perspectives (role-playing scenarios), with ‘exploratory learning’ facilitated through teacher-led small group or dyad discussion, reflection and debriefing, during and after game-play. | Four Dimensional Framework of Learning (de Freitas & Oliver, 2006) underpinned the development of the game. Intervention Mapping (IM) (Bartholomew *et al.*, 1998; Bartholomew *et al.*, 2006) identified psychological targets for the game content. |
| Project Respect | Meiksin *et al.* (2020)  UK | Project Respect is a new intervention, trialled in the UK, and informed by learning from Safe Dates and Shifting Boundaries. This and existing research, including systematic reviews, informed the theory of change, which is underpinned by the theory of planned behaviour and the social development model. After the theory of planned behaviour, the intervention aims to reduce DRV by changing student attitudes and perceived social norms about gender, appropriate behaviours in relationships and violence, and promoting student sense of control over their own behaviour. A key point in the theory of change is that attitudes and norms will be challenged not only by the school curriculum, but by school environmental actions to reduce gender-based harassment on the school site and increase school sanctions against gender-based harassment and DRV. Sense of control over behaviour is encouraged by the curriculum promoting anger management and communication skills. After the social development model, Project Respect enables student participation in curriculum lessons and in leadership campaigns to maximise learning, increase student bonding to school, and acceptance of school behavioural norms. The curriculum also aims to reduce DRV by ensuring those that are exposed to risk can seek early support via the Circle of 6 App and promoting awareness of services.  The intervention is a manualised, multicomponent, school-based, universal prevention intervention. It comprises (i) training for senior leadership team (SLT) by the NSPCC to enable them to plan and deliver intervention; (ii) training by SLT of all other school staff in safeguarding to prevent, recognise and respond to gender-based harassment and DRV; (iii) written information for parents on intervention and advice on recognising and dealing with DRV; (iv) making the Circle of 6 App available to students disguised as a games app; (v) classroom curriculum delivered by teachers, including student-led campaigns. | Theory of planned behaviour (Ajzen, 1985) and social development model (Hawkins & Weis, 1985) |
| Rhode Island Teen Dating Violence Prevention Program (RITDVPP) (DRV) | Silverman (2000)  USA | This ‘multifaceted’ intervention comprises two parts: (i) a primary prevention project for sixth and seventh grade students; and (ii) a secondary prevention project for a subset of students at high risk for victimisation and perpetration of teen dating violence (TDV). The primary prevention intervention is a one-session, 45 minute intervention and the secondary prevention intervention is a seven-session curriculum modified from the Massachusetts Teen Dating Violence Prevention Program.  Both interventions aim to increase awareness of TDV and the attitudes promoting the tolerance of TDV by society such as gender stereotypes and TDV myths. The underpinning assumption to the theory of change is that modifying teens’ attitudes and behaviours could reduce incidents of intimate partner violence over time. The authors state that the logic model for the interventions is to increase student knowledge about abusive and respectful behaviours so that students think about how they would like to be treated in their own dating relationships (before abuses occurs). This in turn would reduce students’ positive attitudes towards couple violence and rape, and thus reduce the likelihood that they would become involved in TDV, either as a victim or perpetrator. | None mentioned |
| Safe Dates (DRV) | Foshee *et al.* (1998)  Foshee (1998)  Foshee *et al.* (2000)  Foshee *et al.* (2004)  Foshee *et al.* (2005)  Gage *et al.* (2016)  USA | Safe Dates is a school-based adolescent dating violence prevention program targeting primary and secondary prevention of dating violence. It comprises school and community activities. The school activities include a theatre production performed by peers, a 10-session curriculum, and a poster contest. The community activities include services for adolescents in abusive relationships (e.g. crisis line and support groups) and community service provider training.  The theory of change is guided by social norms theory, cognitive developmental theory and precaution adoption theory. The authors state that the intervention’s components target ‘theoretically-based mediators’. Changes in norms – both dating violence norms and gender-role norms – and conflict management skills are the theoretical base for primary prevention activities. Secondary prevention activities target cognitive factors such as beliefs in the need for help and awareness of community services that could help. Each mediator is addressed by the intervention’s activities (e.g. changing dating violence norms are addressed by increasing the adolescent’s perception of negative consequences associated with dating violence). Interactive exercises such as completing worksheets with peers, practicing communication skills while role-playing dating situations involving conflict, is an integral part of the curriculum. | Precaution Adoption Theory (Weinstein, 1989), social norms theory (Fishbein & Ajzen, 1975) and cognitive developmental theories (Bem, 1981) |
| SAISIR (Session d'Ateliers Interactifs de Sensibilisation, d'Information et de Réflexion à la violence dans les relations amoureuses des-a (DRV) | Chamberland *et al.* (2014)  France | SAISIR is a dating violence prevention intervention that aims to guide students through the process of developing a mature, responsible attitude towards violence in dating relationships. The design of the intervention is based on a feminist analysis of violence which sees patriarchy as the underlying cause of domestic violence. The design has also been influenced by the developers’ experience of working with women (who have experienced domestic violence) and their children.  SAISIR comprises four structured 75-minute workshops, with one workshop delivered in each nine-day school cycle. The workshops aim to (i) prompt students to think about the problem of violence in teen dating relationships; (ii) inform students about causes and consequences of dating violence; (iii) raise awareness of the sex roles of boys and girls and of the adverse consequences of sexual violence on girls; and (iv) increase student awareness of appropriate attitudes and actions towards girls as victims and boys as perpetrators. The workshop activities included discussions prompted by pictures, games, questions and role-playing.  The mechanisms of change are knowledge and attitudes about violence in teen dating relationships and on increased intentions to act when witnessing violent behaviour. | None mentioned |
| Second Step: Student Success Through Prevention (SS-SSTP) Middle School Program (GBV) | Espelage *et al.* (2013)  Espelage *et al.* (2015b)  Espelage *et al.* (2015a)  Espelage *et al.* (2017)  USA | SS-SSTP is a social-emotional learning programme aiming to reduce youth violence including peer aggression, peer victimisation, homophobic name-calling, sexual violence perpetration and victimisation. It is a curricular classroom intervention with students receiving 41 lessons over three years. The curriculum covers empathy, emotion regulation, communication skills, problem-solving, bully and sexual harassment prevention, and substance abuse prevention. Lessons are delivered in one 50-minute or two 25-minutes sessions, weekly or semi-weekly throughout the year. Student-centred and interactive learning is key with, for example, group discussions, hands-on activities, reflection and role-playing used.  There are several theoretical assumptions underpinning the theory of change for this intervention. It draws on risk and protective factor theory which suggests that ‘problem’ behaviours are rooted in a common overlapping group of risk and protective factors, and so SS-STP targets risk and protective factors linked to aggression, violence and substance use through skill building and skill practice. Social learning theory informs the design and delivery of the curriculum with its emphasis on skills acquisition. Also, social control theory which posits that social control is established through opportunities to interact in positive and prosocial ways with individuals and communities. | Risk and protective factor theory (Rolf *et al.*, 1990; Coie *et al.*, 1993), social learning theory (Bandura, 1977) and social control theory (Hirschi, 2002). |
| School Health Centre Intervention (SHARP) (DRV) | Miller *et al.* (2015)  USA | SHARP is a brief relationship abuse education and counselling program in school health centres to prevent abusive relationships among adolescents (ARA). It addresses a range of abusive behaviours including cyber dating abuse and aims to improve adolescents’ recognition of ARA, use of resources, and attitudes and behaviours to reduce ARA. In each clinical encounter with a young person, healthy and unhealthy relationships are discussed and a palm-size brochure is provided. Each school health centre involves its youth advisory board to organise school-wide outreach events to provide ARA information and encourage students to visit the school health centre.  The mechanisms of change are knowledge with increased recognition of abuse and of relationship abuse resources, attitudes with increased intention to intervene to stop abusive behaviours and self-efficacy to use harm-reduction behaviours. | None mentioned |
| Shifting Boundaries (DRV & GBV) | Mabin (2019)  Taylor *et al.* (2008)  Taylor *et al.* (2010a)  Taylor *et al.* (2010b)  Taylor *et al.* (2011)  Taylor *et al.* (2015)  Taylor *et al.* (2013)  Taylor *et al.* (2017) (DRV)  USA | Shifting Boundaries is a dating violence prevention program comprising classroom-based curricula (covering consequences for perpetrators of DRV, gender roles and healthy relationships) and a building-based intervention (building-based restraining orders, greater faculty and security staff in hot spots, school media campaign).  The theory of change for Shifting Boundaries is informed by the Theory of Reasoned Action (TRA) which is based on research that demonstrates that intentions to behave are immediate predecessors to specific actions. Based on TRA, attitudes towards, and perceived norms about, the desired behaviour facilitate the intention to change, modify or adopt a particular behaviour. The Shifting Boundaries interventions aim to reduce DRV by addressing elements, i.e. increasing knowledge is designed to change attitudes which then affects behavioural intentions and ultimately leads to behavioural change. | Theory of Reasoned Action (Fishbein, 1967; Ajzen & Fishbein, 1980) |
| Skhokho (GBV & DRV) | Jewkes *et al.* (2019)  South Africa | Skhokho is a holistic school intervention to reduce dating and sexual violence. Its goal is to address the drivers of rape and intimate partner violence (IPV) manifest in young people’s social environment. These are outlined in the intervention’s theory of change and are patriarchal gender norms, youth masculinities, protest femininities reifying substance abuse, poor relationship skills and culture of acceptance of violence. The intervention aims to change the manifestations of these drivers i.e. gender norms at home, at school and with peers, alcohol and substance abuse, poor communication (between parent and child and between dating partners), physical and sexual child abuse at home and at school, ineffective, inconsistent and harsh punishment at home, corporal punishment at school and witnessing IPV at home.  The intervention is a schools intervention package and a family intervention, comprising a four day workshop for carers and children on parent teenager relationships. The school intervention comprises a workbook which follows the national curriculum Life Orientation (LO) lesson that covers relationships (content includes gender equity and violence prevention), health, citizenship and preparation for careers and work. The workbook has been co-authored with a Grade 8 learner and has an accessible and youthful style. The workbook material has a strong gender lens and the exercises emphasise learning by self-study. Teachers receive training on the materials, with a guide and model answers for the activities, and training on positive discipline and classroom management, stress and coping, and putting policies and values into action. Positive discipline training is offered to all school staff. The families’ workshops aim to strengthen relationships and are conducted in separate peer groups with a dialogue session at the end of each day to encourage caregiver teenager communication. The emphasis is on participatory learning approaches such as critical reflection, drama and skills building. | Educational theory summarised by Morrell *et al.* (2009) |
| Speak: Literary Instruction on Rape Myth Acceptance (GBV) | Malo-Juvera (2012)  Malo-Juvera (2014)  USA | This literary intervention uses a young adult novel, *Speak*, as part of English Language arts classes to reduce adolescent rape myth acceptance. The novel details the aftermath of a date rape survived by a girl who is assaulted before her first year of high school. The theory of change draws on reader response theory (Rosenblatt, 1978) to suggest that readers can vicariously experience what characters do; such experience may broaden their emotional, intellectual and cultural horizons by allowing identification with characters that differ in age, gender, socioeconomic status, race, religion, sexual orientation and geographic location. The reading transaction may occur on a continuum between aesthetic and efferent with the possible benefits of transactional reading more likely to occur in literary aesthetic events. This is influenced by the learning approach and ‘dialogic instruction’ provides students with the ability to talk to learn, invites discussion that invites students to interact with each other, helps students to discuss and consider controversies and different points of view.  The intervention is a five-week unit (12 classes) grounded in reader response-based dialogic instruction about *Speak*. It uses four major reader response writing assignments that were completed in class. Writing is followed by small-group discussion, followed by whole class discussion. There is minimal teacher intervention to enable genuine discussion that could include divergent views and opinions. In the large-group discussion, teachers encourage students to discuss and explore different perspectives. | Reader response theory (Rosenblatt, 1978) |
| TakeCARE (DRV & GBV) | Jouriles *et al.* (2019)  Sargent *et al.* (2017)  USA | This intervention is a brief video bystander programme (less than 30 minutes long) which aims to promote self-efficacy by presenting students with several different risky situations as well as situations in which violence has occurred. For each situation the video models and describes possible actions that could be taken to reduce the risk that violence might follow and support the victims of the violence. TakeCARE offers students concrete examples of what they can say or do, focusing on the importance of taking ‘some’ action to help. The intervention is based on the theory that suggests in order for bystanders to act, they need to feel confident in their ability to intervene effectively, i.e. have self-efficacy. | Self-efficacy (Latané & Darley, 1970; Burn, 2009; Banyard, 2011) |
| Teen VIP (Teen Dating Violence Intervention and Prevention) (DRV) | Miller (1998)  USA | This intervention follows the group counselling model which is designed for children who live or have lived with violence at home or who have a history of dating violence. It is a five-day program, delivered over 10 weeks. The authors argue that social learning theory provides a comprehensive explanation for the mechanisms involved in understanding domestic violence and how values, attitudes, beliefs and behaviours develop at the individual level.  The intervention aims to stop the cycle of violence, teach healthy peer relationships, assertiveness and communication skills, techniques to control anger, and the self-efficacy to use these skills and techniques There were opportunities to explore feelings and personal experiences. Learning by role-playing is an integral component of social learning theory and is an important technique to help children model and practise new behaviours to counter aggression and violence. | Social learning theory (Bandura, 1973; Bandura & Walters, 1977; Bandura, 1986)) and cycle theory of violence (Walker, 1995) |
| Teen Choices (DRV) | Levesque *et al.* (2016)  Levesque *et al.* (2017)  NCT (2015)  <https://clinicaltrials.gov/ct2/show/NCT02458365>  Levesque (2007)  USA | This intervention comprises a three session online programme that delivers assessments and individualised guidance matched to dating history, dating violence experiences, and stages of readiness for using healthy relationship skills. It is a web-based multimedia (text, images, audio, video) expert system intervention that integrates in a stage-matched manner, key content (e.g. warning signs, statistics on dating violence), and activities (e.g. expectations regarding the balance of power in dating relationships) found in evidence-based dating violence prevention programmes. The intervention is individually tailored, with five intervention tracks to meet the unique needs of (a) high-risk victims, (b) high-risk daters, (c) low-risk daters, (d) high-risk non-daters, and (e) low-risk daters. For high-risk victims, the program focuses on progressing through the stages of change for keeping oneself safe in relationships. Sessions last 25-30 minutes.  The theory of change for Teen Choices is based on an evidence-based model of health behaviour change, the Transtheoretical Model. Behaviour change involves progress through a series of stages of change – Precontemplation, Contemplation, Preparation, Action and Maintenance. Teen Choices aims to reduce risk for dating violence by facilitating progress through the stages of change for using five healthy relationship skills: (a) trying to understand and respect the other person’s feeling and needs; (b) using calm, non-violent ways to deal with disagreements; (c) respecting the other person’s boundaries; (d) communicating needs and feelings clearly and respectfully; and (e) making decisions that you know that are good for you in relationships. Videos demonstrate how to use two skills that the students have been using the least. | Transtheoretical Model (TTM) (Prochaska & DiClemente, 1983) |
| Twilight, True Love and you (DRV) | Lynch (2014)  England | This intervention uses a bibliotherapy approach using ‘Twilight, True Love and you’ written by a clinical psychologist as a resource to help prevent dating abuse in adolescent girls. It aims to engage the reader through using the teen romantic fantasy series to highlight what a woman should look for in a partner and what may be a warning sign of dating abuse.  The theory of change is informed by the theory of planned behaviour (Ajzen, 1991) and aims to change romantic myths about relationships such as ‘love at first sight’ and ‘women can change their men’. The underlying assumption is that these are likely to influence adolescents’ cultural norms about dating abuse with the result that they may be more tolerant of dating abuse. If these norms and attitudes change then intentions around involvement in an abusive relationship may change. The mechanisms of change include belief in common romantic myths, knowledge of warning signs of dating abuse, reduction in violent tolerant attitudes, behavioural intention to date someone displaying warning signs and reporting dating abuse. | Theory of planned behaviour (Ajzen, 1991) |
| Web-based dating violence prevention program (DRV) | Jung & Min (2013)  South Korea | This web-based intervention aims to prevent dating violence in middle school students by increasing awareness of dating violence, modifying gender role attitudes and reducing aggression. It is composed of 13 topics across five areas and is organised as eight sessions (90 minutes per session). It is delivered as part of small group learning activities. The ABCDE model of behavioural therapy provides the theoretical basis for the intervention and aims to change irrational beliefs and misconceptions (i.e. gender role attitudes) to improve the response and so reduce anger and aggression. | ABCDE model of behavioral therapy theory (Ellis & MacLaren, 1998) |
| You-Me-Us (DRV) | Coyle *et al.* (2019)  USA | This intervention is a healthy relationships-based program comprising a classroom curriculum and a social norms campaign. The 12-session curriculum is theoretically grounded in social cognitive theory (Bandura, 1986) and includes content on building healthy friendships and relationships, communicating effectively, influences on sexual expectations in relationships, personal boundaries, dealing with situations that could challenge personal boundaries and ending unhealthy relationships. The peer-norms campaign is led by students who implement projects such as creating a resource area to convey healthy relationship messages across the school. The mechanisms of action seem to be increased sexual-health related knowledge, attitudes, beliefs, perceived norms, improved parent-child communication and avoidance of risky situations. | Social cognitive theory (Bandura, 1986) and positive youth development (Damon, 2004) |

**References**

Achyut, P., Bhatla, N., Khandekar, S., Maitra, S. & Verma, R. K. (2011). Building support for gender equality among young adolescents in school: Findings from Mumbai, India. (New Delhi, India, International Center for Research on Women).

Achyut, P., Bhatla, N., Verma, H., Uttamacharya, S. G., Bhattacharya, S. & Verma, R. (2016). Towards gender equality. The GEMS journey thus far. An evaluation report of the Gender Equity Movement in Schools (GEMS) program in Jharkhand. (New Delhi, India, International Center for Research on Women).

Achyut, P., Bhatla, N. & Verma, R. (2015). Questioning gender norms to promote sexual reproductive health among early adolescents: Evidence from a school program in Mumbai, India. In: Y. K. Djamba & S. R. Kimuna (Ed) *Gender-based violence: Perspectives from Africa, the Middle East, and India* (Switzerland, Springer International): 195-213.

Ajzen, I. (1985). From intentions to actions: A theory of planned behavior. In: J. Kuhl & J. Beckmann (Ed) *Action control from cognition to behavior* (Berlin, Germany, Springer Verlag).

Ajzen, I. (1991). The theory of planned behavior. *Organizational Behavior and Human Decision Processes,* 50(2), 179-211.

Ajzen, I. & Fishbein, M. (1980). *Understanding attitudes and predicting social behavior*. (Englewood Cliffs, NJ, Prentice-Hall).

Ajzen, I. & Madden, T. J. (1986). Prediction of goal-directed behavior: Attitudes, intentions, and perceived behavioral control. *Journal of Experimental Social Psychology,* 22(5), 453-474.

Arnab, S., Brown, K., Clarke, S., Dunwell, I., Lim, T., Suttie, N., Louchart, S., Hendrix, M. & de Freitas, S. (2013). The development approach of a pedagogically-driven serious game to support Relationship and Sex Education (RSE) within a classroom setting. *Computers and Education,* 69, 15-30.

Arnab, S., Brown, K., Clarke, S., Judd, B., Baxter, J., King, R., Joshi, P., Newby, K. & Bayley, J. (2012). Serious game for Relationships and Sex Education (RSE): Promoting discourse on pressure and coercion in adolescent relationships. *Proceedings of the European Conference on Games Based Learning,* 1, 611-614.

Austin, E. W. & Johnson, K. K. (1997). Immediate and delayed effects of media literacy training on third grader's decision making for alcohol. *Health Communication,* 9(4), 323-349.

Avery-Leaf, S., Cascardi, M., O'Leary, K. D. & Cano, A. (1997). Efficacy of a dating violence prevention program on attitudes justifying aggression. *Journal of Adolescent Health,* 21(1), 11-17.

Baiocchi, M., Omondi, B., Langat, N., Boothroyd, D. B., Sinclair, J., Pavia, L., Mulinge, M., Githua, O., Golden, N. H. & Sarnquist, C. (2017). A behavior-based intervention that prevents sexual assault: The results of a matched-pairs, cluster-randomized study in Nairobi, Kenya. *Prevention Science,* 18(7), 818-827.

Bando, R., Hidalgo, N. & Land, A. (2018). El efecto de la educación en las actitudes de género: Evidencia experimental en educación secundaria en El Salvador, Banco Interamericano de Desarrollo)**:** 55.

Bando, R., Hidalgo, N. & Land, A. (2019). Education with a social focus on gender attitudes: experimental evidence from secondary education in El Salvador. *Journal of Economics, Race, and Policy,* 2(4), 225-239.

Bandura, A. (1973). Social learning theory of aggression. In: J. F. Knutson (Ed) *In the control of aggression* (Chicago, IL, Aldine).

Bandura, A. (1977). Self-efficacy: toward a unifying theory of behavioral change. *Psychological Review,* 84(2), 191-215.

Bandura, A. (1986). *Social foundations of thought and action: A social cognitive theory*. (Englewood Cliffs, NJ, Prentice-Hall).

Bandura, A. & Walters, R. H. (1977). *Social learning theory*. (Englewood Cliffs, NJ, Prentice-Hall).

Banyard, V. L. (2011). Who will help prevent sexual violence: Creating an ecological model of bystander intervention. *Psychology of Violence,* 1, 216-229.

Bartholomew, L. K., Parcel, G. S. & Kok, G. (1998). Intervention mapping: a process for developing theory- and evidence-based health education programs. *Health Education and Behavior,* 25(5), 545-563.

Bartholomew, L. K., Parcel, G. S., Kok, G., Gottlieb, N. H. & Fernandez, M. E. (2006). *Planning health promotion programs; an intervention mapping approach*. (San Francisco, CA, Jossey-Bass).

Bem, S. L. (1981). Gender schema theory: A cognitive account of sex typing. *Psychological Review,* 88(4), 354-364.

Berkowitz, A. D. (2002). Fostering men's responsibility for preventing sexual assault. In: P. A. Schewe (Ed) *Preventing violence in relationships: Interventions across the life span.* (Washington, DC, US, American Psychological Association): 163-196.

Boduszek, D., Debowska, A., Jones, A. D., Ma, M., Smith, D., Willmott, D., Trotman Jemmott, E., Da Breo, H. & Kirkman, G. (2019). Prosocial video game as an intimate partner violence prevention tool among youth: A randomised controlled trial. *Computers in Human Behavior,* 93, 260-266.

Bronfenbrenner, U. (1996). *A ecologia do desenvolvimento humano: experimentos naturais e planejados*. (Porto Alegre, Artes Médicas).

Bronfenbrenner, U. (2004). *Making human beings human: Bioecological perspectives on human development*. (London, UK, Sage).

Bronfenbrenner, U. & Evans, G. W. (2000). Developmental science in the 21st century: Emerging questions, theoretical models, research designs and empirical findings. *Social Development,* 9(1), 115-125.

Brown, K., Arnab, S., Bayley, J., Newby, K., Joshi, P., Judd, B., Baxter, A. & Clarke, S. (2012). Tackling sensitive issues using a game-based environment: serious game for relationships and sex education (RSE). *Annual Review of Cybertherapy and Telemedicine,* 181, 165-171.

Bryan, J. H. & Test, M. A. (1967). Models and helping: naturalistic studies in aiding behavior. *Journal of Personality and Social Psychology,* 6(4), 400-407.

Buckley, K. E. & Anderson, C. A. (2006). A theoretical model of the effects and consequences of playing video games. In: P. Vorderer & J. Bryant (Ed) *Playing video games: Motives, responses, and consequences* (Mahwah, NJ, LEA): 363-378.

Burkhart, B. R. F., M. E. (1991). Individual and social psychological understanding of sexual coercion. In: E. Grauerholz & M. A. Koralewski (Ed) *Sexual coercion: A sourcebook on it nature, causes, and prevention* (Lexington, MA, New Lexington Press): 75-89.

Burn, S. M. (2009). A situational model of sexual assault prevention through bystander intervention. *Sex Roles,* 60(11), 779-792.

Capaldi, D. M., Shortt, J. W. & Kim, H. K. (2005). A life span developmental systems perspective on aggression toward a partner. (Ed) *Family psychology: The art of the science.* (New York, NY, US, Oxford University Press): 141-167.

Cardoso, H. L. & Oliveira, E. (2011). Social control in a normative framework: An adaptive deterrence approach. *Web Intelligence and Agent Systems: An International Journal,* 9, 363-375.

Chamberland, A., Cantin-Drouin, M. & Damant, D. (2014). Assessment of the impact of Saisir: A dating violence prevention program. *Canadian Social Work Review/Revue Canadienne de Service Social,* 31(1), 125-139.

Chekroun, P. & Brauer, M. (2002). The bystander effect and social control behavior: the effect of the presence of others on people's reactions to norm violations. *European Journal of Social Psychology,* 32(6), 853-867.

Cissner, A. B. & Ayoub, L. H. (2014). Building healthy teen relationships: An evaluation of the Fourth R Curriculum with middle school students in the Bronx: U.S. (Washington, DC, Center for Court Innovation).

Clark, R. D. & Word, L. E. (1974). Where is the apathetic bystander? Situational characteristics of the emergency. *Journal of Personality and Social Psychology,* 29, 279-287.

Coie, J. D., Watt, N. F., West, S. G., Hawkins, J. D., Asarnow, J. R., Markman, H. J., Ramey, S. L., Shure, M. B. & Long, B. (1993). The science of prevention: A conceptual framework and some directions for a national research program. *American Psychologist,* 48(10), 1013-1022.

Coker, A. L., Bush, H. M., Brancato, C. J., Clear, E. R. & Recktenwald, E. A. (2019). Bystander program effectiveness to reduce violence acceptance: RCT in high schools. *Journal of Family Violence,* 34(3), 153-164.

Coker, A. L., Bush, H. M., Brancato, C. J., Huang, Z., Clear, E. R. & Follingstad, D. R. (2020a). Longer term impact of bystander training to reduce violence acceptance and sexism. *Journal of School Violence,* 19(4), 525-538.

Coker, A. L., Bush, H. M., Clear, E. R., Brancato, C. J. & McCauley, H. L. (2020b). Bystander program effectiveness to reduce violence and violence acceptance within sexual minority male and female high school students using a cluster RCT. *Prevention Science,* 21(3), 434-444.

Coker, A. L., Bush, H. M., Cook-Craig, P. G., DeGue, S. A., Clear, E. R., Brancato, C. J., Fisher, B. S. & Recktenwald, E. A. (2017). RCT testing bystander effectiveness to reduce violence. *American Journal of Preventive Medicine,* 52(5), 566-578.

Coker, A. L., Bush, H. M., Huang, Z., Brancato, C. J., Clear, E. R. & Follingstad, D. R. (2021). How does Green Dot bystander training in high school and beyond impact attitudes toward violence and sexism in a prospective cohort? *Journal of Interpersonal Violence*, 8862605211006354.

Coyle, K. K., Anderson, P., Franks, H. M., Walker, J. D. & Glassman, J. R. (2019). You-Me-Us: Results of a cluster randomized trial of a healthy relationships approach to sexual risk reduction. *Journal of Primary Prevention,* 40(6), 607-629.

Damon, W. (2004). What is positive youth development? *Annals Of the American Academy of Political and Social Science,* 591(1), 13-24.

Darley, J. M. & Latane, B. (1968). Bystander intervention in emergencies: Diffusion of responsibility. *Journal of Personality and Social Psychology,* 8(4, Pt.1), 377-383.

Davidson, A. R. & Morrison, D. M. (1983). Predicting contraceptive behavior from attitudes: A comparison of within- versus across-subjects procedures. *Journal of Personality and Social Psychology,* 45(5), 997-1009.

de Freitas, S. & Oliver, M. (2006). How can exploratory learning with games and simulations within the curriculum be most effectively evaluated? *Computers & Education,* 46(3), 249-264.

de Lijster, G. P., Felten, H., Kok, G. & Kocken, P. L. (2016). Effects of an interactive school-based program for preventing adolescent sexual harassment: A cluster-randomized controlled evaluation study. *Journal of Youth and Adolescence,* 45(5), 874-886.

Decker, M. R., Wood, S. N., Ndinda, E., Yenokyan, G., Sinclair, J., Maksud, N., Ross, B., Omondi, B. & Ndirangu, M. (2018). Sexual violence among adolescent girls and young women in Malawi: A cluster-randomized controlled implementation trial of empowerment self-defense training. *BMC Public Health,* 18(1), 1341.

DeGue, S., Niolon, P. H., Estefan, L. F., Tracy, A. J., Le, V. D., Vivolo-Kantor, A. M., Little, T. D., Latzman, N. E., Tharp, A., Lang, K. M. & Taylor, B. (2021). Effects of Dating Matters® on sexual violence and sexual harassment outcomes among middle school youth: A cluster-randomized controlled trial. *Prevention Science,* 22(2), 175-185.

Devries, K. M., Knight, L., Allen, E., Parkes, J., Kyegombe, N. & Naker, D. (2017). Does the Good Schools Toolkit reduce physical, sexual and emotional violence, and injuries, in girls and boys equally? A cluster-randomised controlled trial. *Prevention Science,* 18(7), 839-853.

Dobash, R. E. & Dobash, R. P. (1979). *Violence against wives: A case against the patriarchy*. (New York, NY, Free Press).

dos Santos, K. B., Murta, S. G., Vinha, L. G. D. & de Deus, J. S. (2019). Efficacy of a bystander intervention for preventing dating violence in Brazilian adolescents: short-term evaluation. *Psicologia: Reflexão e Critica,* 32(1), 14.

Durand, A. J. (1997). *Sexual harassment and junior high school students: The effect of a program designed to address peer-to-peer sexual harassment (PhD thesis)*, Colorado State University.

Edwards, K. M., Banyard, V. L., Sessarego, S. N., Waterman, E. A., Mitchell, K. J. & Chang, H. (2019). Evaluation of a bystander-focused interpersonal violence prevention program with high school students. *Prevention Science,* 20(4), 488-498.

Ekhtiari, S. Y., Shojaeizadeh, D., Rahimi Foroushani, A., Ghofranipour, F. & Ahmadi, B. (2013). The effect of an intervention based on the PRECEDE- PROCEED model on preventive behaviors of domestic violence among Iranian high school girls. *Iranian Red Crescent Medical Journal,* 15(1), 21-28.

Ekhtiari, Y. S., Shojaeizadeh, D., Foroushani, A. R., Ghofranipour, F. & Ahmadi, B. (2014). Effect of an intervention on attitudes towards domestic violence among Iranian girls. *Journal of the Pakistan Medical Association,* 64(9), 987-992.

Elfeddali, I., Bolman, C., Candel, M. J., Wiers, R. W. & De Vries, H. (2012). The role of self-efficacy, recovery self-efficacy, and preparatory planning in predicting short-term smoking relapse. *British Journal of Health Psychology,* 17(1), 185-201.

Ellis, A. & MacLaren, C. (1998). *Rational emotive behavior therapy: A therapist's guide*. (Atascadero, CA, Impact Publishers).

Espelage, D. L., Bub, K., Van Ryzin, M. & Holt, M. K. (2017). *Effects of a middle school social-emotional learning program on bullying, teen dating violence, sexual violence, and substance use in high school*, National Institute of Justice, National Criminal Justice Reference Service).

Espelage, D. L., Low, S., Polanin, J. R. & Brown, E. C. (2013). The impact of a middle school program to reduce aggression, victimization, and sexual violence. *Journal of Adolescent Health,* 53(2), 180-186.

Espelage, D. L., Low, S., Polanin, J. R. & Brown, E. C. (2015a). Clinical trial of Second Step© middle-school program: Impact on aggression & victimization. *Journal of Applied Developmental Psychology,* 37, 52-63.

Espelage, D. L., Low, S., Van Ryzin, M. J. & Polanin, J. R. (2015b). Clinical trial of Second Step Middle School Program: Impact on bullying, cyberbullying, homophobic teasing, and sexual harassment perpetration. *School Psychology Review,* 44(4), 464-479.

Fay, K. E. & Medway, F. J. (2006). An acquaintance rape education program for students transitioning to high school. *Sex Education,* 6(3), 223-236.

Fernández-González, L. C., E. & Sánchez-Álvarez, N. (2020). Efficacy of a brief intervention based on an incremental theory of personality in the prevention of adolescent dating violence: A randomized controlled trial. *Psychosocial Intervention,* 29(1), 9-18.

Filho, S. R. P. (2017). *Avaliação de uma intervenção para prevenção da violência no namoro (PhD thesis)*, Universidade Federal de São Carlos.

Fishbein, M. (1967). *Readings in attitude theory and measurement*. (Oxford, England, Wiley).

Fishbein, M. & Ajzen, I. (1975). *Belief, attitude, intention and behavior: an introduction to theory and research*. (Reading, MA, Addison-Wesley).

Fishbein, M. & Ajzen, I. (2010). *Predicting and changing behavior: The reasoned action approach*. (New York, NY, Psychology Press).

Flay, B. R. & Petraitis, J. (1994). The theory of triadic influence: a new theory of health behavior with implications for preventive interventions. In: G. Albrecht (Ed) *Advances in Medical Sociology. Vol. 4: A Reconsideration of Models of Health Behavior Change* (Greenwich, CT, JA1 Press): 19-44.

Foshee, V. A. (1998). Involving schools and communities in preventing adolescent dating abuse. In: X. B. Arriaga & S. Oskamp (Ed) *Addressing community problems: Psychological research and interventions* (Thousand Oaks, CA, Sage Publications): 104-129.

Foshee, V. A., Bauman, K. E., Arriaga, X. B., Helms, R. W., Koch, G. G. & Linder, G. F. (1998). An evaluation of Safe Dates, an adolescent dating violence prevention program. *American Journal of Public Health,* 88(1), 45-50.

Foshee, V. A., Bauman, K. E., Ennett, S. T., Linder, G. F., Benefield, T. & Suchindran, C. (2004). Assessing the long-term effects of the Safe Dates program and a booster in preventing and reducing adolescent dating violence victimization and perpetration. *American Journal of Public Health,* 94(4), 619-624.

Foshee, V. A., Bauman, K. E., Ennett, S. T., Suchindran, C., Benefield, T. & Linder, G. F. (2005). Assessing the effects of the dating violence prevention program "safe dates" using random coefficient regression modeling. *Prevention Science,* 6(3), 245-258.

Foshee, V. A., Bauman, K. E., Greene, W. F., Koch, G. G., Linder, G. F. & MacDougall, J. E. (2000). The Safe Dates program: 1-year follow-up results. *American Journal of Public Health,* 90(10), 1619-1622.

Gage, A. J., Honoré, J. G. & Deleon, J. (2016). Pilot test of a dating violence-prevention curriculum among high school students: Emerging evidence of effectiveness in a low-income country. .

Garmaroudi, G., Sarlak, B. & Sadeghi, R. (2016). بررسی تأثیر مداخله آموزشی براساس الگوي اعتقاد بهداشتي بر رفتارهای ‌پيشگيري‌كننده از خشونت خانگی در دانش‌آموزان دختر دوره دوم متوسطه شهر تهران‎. *Journal of Knowledge and Health in Basic Medical Sciences,* 11(1), 1395.

Gerrard, M., Gibbons, F. X., Houlihan, A. E., Stock, M. L. & Pomery, E. A. (2008). A dual-process approach to health risk decision making: The prototype willingness model. *Developmental Review,* 28(1), 29-61.

Gielen, A. C., McDonald, E. M., Gary, T. L. & Bone, L. R. (2008). Using the Precede-Proceed model to apply health behavior theories. In: K. Glanz, B. K. Rimer & L. F. M. (Ed) *Health behavior and health education: Theory, research and practice* (San Francisco, CA, Jossey-Bass): 407-433.

Gonzalez-Guarda, R. M., Guerra, J. E., Cummings, A. A., Pino, K. & Becerra, M. M. (2015). Examining the preliminary efficacy of a dating violence prevention program for Hispanic adolescents. *Journal of School Nursing,* 31(6), 411-421.

Hawkins, J. D. & Weis, J. G. (1985). The social development model: An integrated approach to delinquency prevention. *Journal of Primary Prevention,* 6(2), 73-97.

Hirschi, T. (2002). *Causes of delinquency*. (New Brunswick, NJ, Transaction).

ICRW (2017). Changing course. Implementation and evaluation of the Gender Equity Movement in Schools (GEMS) program in specific sites - Vietnam, India and Bangladesh. (New Delhi, India, International Center for Research on Women).

Jaycox, L. H., McCaffrey, D., Eiseman, B., Aronoff, J., Shelley, G. A., Collins, R. L. & Marshall, G. N. (2006a). Impact of a school-based dating violence prevention program among Latino teens: randomized controlled effectiveness trial. *Journal of Adolescent Health,* 39(5), 694-704.

Jaycox, L. H., McCaffrey, D. F., Weidmer Ocampo, B., Marshall, G. N., Collins, R. L., Hickman, L. J. & Quigley, D. D. (2006b). Curbing teen dating violence: Evidence from a school prevention program. RB-9194-CDC. (Santa Monica, CA, RAND Corporation).

Jemmott, J. B., 3rd, O'Leary, A., Jemmott, L. S., Ngwane, Z. P., Teitelman, A. M., Makiwane, M. B. & Bellamy, S. L. (2018). Effect of a behavioral intervention on perpetrating and experiencing forced sex among South African adolescents: A secondary analysis of a cluster randomized trial. *JAMA Network Open,* 1(4), e181213.

Jewkes, R., Gevers, A., Chirwa, E., Mahlangu, P., Shamu, S., Shai, N. & Lombard, C. (2019). RCT evaluation of Skhokho: A holistic school intervention to prevent gender-based violence among South African Grade 8s. *PLoS ONE,* 14(10), e0223562.

Johnson, R., Gilchrist, E., Beech, A. R., Weston, S., Takriti, R. & Freeman, R. (2006). A psychometric typology of U.K. domestic violence offenders. *Journal of Interpersonal Violence,* 21(10), 1270-1285.

Joppa, M. C., Rizzo, C. J., Nieves, A. V. & Brown, L. K. (2016). Pilot investigation of the Katie Brown Educational Program: A school-community partnership. *Journal of School Health,* 86(4), 288-297.

Jouriles, E. N., McDonald, R., Rosenfield, D. & Sargent, K. S. (2019). Increasing bystander behavior to prevent adolescent relationship violence: A randomized controlled trial. *Journal of Consulting and Clinical Psychology,* 87(1), 3-15.

Jung, H.-Y. & Min, H.-S. (2013). Development & evaluation of web-based dating violence prevention program for middle school students. *Journal of Korean Academic Society of Nursing Education,* 19(4), 627-639.

Kershner, R. E. (1995). *The efficacy of adolescent rape prevention education (PhD thesis)*, West Virginia University.

Latané, B. & Darley, J. M. (1970). *The unresponsive bystander: Why doesn't he help?* (New York, NY, Appleton-Century Crofts).

Lee, P. S., Chen, H. T. & Hong, Z. R. (2018). Effects of a bystander intervention curriculum for preventing sexual harassment. *Journal of Research in Education Sciences,* 63(3), 1-35.

Levesque, D. A. (2007). A stage-based expert system for teen dating violence prevention. Final Progress Report. (South Kingstown, RI, Pro-Change Behavior Systems, Inc.).

Levesque, D. A., Johnson, J. L. & Prochaska, J. M. (2017). Teen Choices, an online stage-based program for healthy, nonviolent relationships: Development and feasibility trial. *Journal of School Health,* 16(4), 376-385.

Levesque, D. A., Johnson, J. L., Welch, C. A., Prochaska, J. M. & Paiva, A. L. (2016). Teen dating violence prevention: Cluster-randomized trial of teen choices, an online, stage-based program for healthy, nonviolent relationships. *Psychology of Violence,* 6(3), 421-432.

Lisak, D. & Miller, P. M. (2002). Repeat rape and multiple offending among undetected rapists. *Violence and Victims,*(1), 73-84.

Lisak, D. & Roth, S. (1988). Motivational factors in nonincarcerated sexually aggressive men. *Journal of Personality and Social Psychology,* 55(5), 795-802.

Lynch, A. (2014). *Twilight, True Love and You: a bibliotherapy approach to preventing dating abuse in adolescent girls (PhD thesis)*, University of Surrey.

Mabin, A. (2019). *Effectiveness of a teen dating violence prevention program in middle schools (MSc thesis)*, Syracuse University.

Macgowan, M. J. (1997). An evaluation of a dating violence prevention program for middle school students. *Violence and Victims,* 12(3), 223-235.

Malo-Juvera, V. (2012). *The effect of young adult literature on adolescents' rape myth acceptance (Doctoral thesis)*, Florida International University.

Malo-Juvera, V. (2014). Speak: The effect of literary instruction on adolescents' rape myth acceptance. *Research in the Teaching of English,* 48(4), 407-427.

Mathews, C., Eggers, S. M., Townsend, L., Aaro, L. E., de Vries, P. J., Mason-Jones, A. J., De Koker, P., McClinton Appollis, T., Mtshizana, Y., Koech, J., Wubs, A. & De Vries, H. (2016). Effects of PREPARE, a multi-component, school-based HIV and intimate partner violence (IPV) prevention programme on adolescent sexual risk behaviour and IPV: Cluster randomised controlled trial. *AIDS and Behavior,* 20(9), 1821-1840.

McGuire, W. (1972). Social psychology. In: P. C. Dodwell (Ed) *New horizons in psychology 2* (Middlesex, UK, Penguin Books). 2: 219-242.

Meiksin, R., Crichton, J., Dodd, M., Morgan, G. S., Williams, P., Willmott, M., Allen, E., Tilouche, N., Sturgess, J., Morris, S., Barter, C., Young, H., Melendez-Torres, G. J., Taylor, B., Reyes, H., Elbourne, D., Sweeting, H., Hunt, K., Ponsford, R., Campbell, R. & Bonell, C. (2020). A school intervention for 13- to 15-year-olds to prevent dating and relationship violence: The Project Respect pilot cluster RCT. *Public Health Research,* 8(5), 1-337.

Merrell, R. (2004). *The impact of a drama intervention program on the response of the bystander to bullying situations (Doctoral thesis)*, University of Rochester.

Miller, E., Goldstein, S., McCauley, H. L., Jones, K. A., Dick, R. N., Jetton, J., Silverman, J. G., Blackburn, S., Monasterio, E., James, L. & Tancredi, D. J. (2015). A school health center intervention for abusive adolescent relationships: a cluster RCT. *Pediatrics,* 135(1), 76-85.

Miller, E., Jones, K. A., Ripper, L., Paglisotti, T., Mulbah, P. & Abebe, K. Z. (2020). An athletic coach-delivered middle school gender violence prevention program: A cluster randomized clinical trial. *JAMA Pediatrics,* 174(3), 241-249.

Miller, E., Tancredi, D. J., McCauley, H. L., Decker, M. R., Virata, M. C., Anderson, H. A., Stetkevich, N., Brown, E. W., Moideen, F. & Silverman, J. G. (2012). "Coaching Boys into Men": A cluster-randomized controlled trial of a dating violence prevention program. *Journal of Adolescent Health,* 51(5), 431-438.

Miller, E., Tancredi, D. J., McCauley, H. L., Decker, M. R., Virata, M. C. D., Anderson, H. A., O'Connor, B. & Silverman, J. G. (2013). One-year follow-up of a coach-delivered dating violence prevention program: a cluster randomized controlled trial. *American Journal of Preventive Medicine,* 45(1), 108-112.

Miller, J. R. (1998). *A social learning perspective toward the prevention of dating violence: An evaluation of a group counseling model (PhD thesis)*, Miami Institute of Psychology of the Caribbean Center for Advanced Studies.

Montano, D. E. & Kasprzyk, D. (2008). Theory of reasoned action, theory of planned behavior, and the integrated behavioral model. In: K. Glanz, B. K. Rimer & K. Viswanath (Ed) *Health behavior and health education: Theory, research and practice* (San Francisco, CA, Jossey-Bass): 67-96.

Morrell, R., Epstein, D., Unterhalter, E., Bhana, D. & Moletsane, R. (2009). *Towards gender equality: South African schools during the HIV and AIDS epidemic*. (Pietermaritzburg, South Africa, University of KwaZulu-Natal Press).

Muck, C., Schiller, E. M. & Kartner, J. (2018a). Prevention of sexual violence in adolescence - evaluation study on the effect of two school-based prevention programs on the willingness for disclosure and the experience of victimization. *Zeitschrift für Soziologie der Erziehung und Sozialisation,* 38(2), 154-170.

Muck, C., Schiller, E. M., Zimmermann, M. & Kartner, J. (2018b). Preventing sexual violence in adolescence: Comparison of a scientist-practitioner program and a practitioner program using a cluster-randomized design. *Journal of Interpersonal Violence,* 36(3-4), NP1913-1940NP.

Munoz-Fernandez, N., Ortega-Rivera, J., Nocentini, A., Menesini, E. & Sanchez-Jimenez, V. (2019). The efficacy of the "Dat-E Adolescence" prevention program in the reduction of dating violence and bullying. *International Journal of Environmental Research and Public Health,* 16(3), 31.

Munoz-Rivas, M. J., Redondo-Rodriguez, N. & Ronzon-Tirado, R. C. (2019). Dating violence prevention: Evaluation of the program PREVIO. *Revista de Psicologia Clinica con Niños y Adolescentes,* 6(3), 18-23.

Myrick, R. D. (1993). *Developmental guidance and counseling: A practical approach*. (Minneapolis, MN, Educational Media Corporation).

NCT (2015). A stage-based expert system for teen dating violence prevention. *ClinicalTrials.gov*.

Niolon, P. H., Vivolo-Kantor, A. M., Tracy, A. J., Latzman, N. E., Little, T. D., DeGue, S., Lang, K. M., Estefan, L. F., Ghazarian, S. R., McIntosh, W. L. K., Taylor, B., Johnson, L. L., Kuoh, H., Burton, T., Fortson, B., Mumford, E. A., Nelson, S. C., Joseph, H., Valle, L. A. & Tharp, A. T. (2019). An RCT of Dating Matters: Effects on teen dating violence and relationship behaviors. *American Journal of Preventive Medicine,* 57(1), 13-23.

Olweus, D. (1993). *Bullying at school: What we know and what we can do*. (Oxford, UK, Wiley).

Olweus, D., Limber, S. & Mihalic, S. (1999). Blueprints for violence prevention, book nine: Bullying prevention program. (Ed) (Boulder, CO, Center for the Study and Prevention of Violence). 12: 256-273.

Pacifici, C., Stoolmiller, M. & Nelson, C. (2001). Evaluating a prevention program for teenagers on sexual coercion: a differential effectiveness approach. *Journal of Consulting and Clinical Psychology,* 69(3), 552-559.

Pantin, H., Schwartz, S. J., Sullivan, S., Prado, G. & Szapocznik, J. (2004). Ecodevelopmental HIV prevention programs for Hispanic adolescents. *American Journal of Orthopsychiatry,* 74(4), 545-558.

Peskin, M. F., Markham, C. M., Shegog, R., Baumler, E. R., Addy, R. C., Temple, J. R., Hernandez, B., Cuccaro, P. M., Thiel, M. A., Gabay, E. K. & Tortolero Emery, S. R. (2019). Adolescent dating violence prevention program for early adolescents: The Me & You randomized controlled trial, 2014-2015. *American Journal of Public Health,* 109(10), 1419-1428.

Peskin, M. F., Markham, C. M., Shegog, R., Baumler, E. R., Addy, R. C. & Tortolero, S. R. (2014). Effects of the It's Your Game . . . Keep It Real program on dating violence in ethnic-minority middle school youths: a group randomized trial. *American Journal of Public Health,* 104(8), 1471-1477.

Poletto, M. & Koller, S. H. (2008). Contextos ecológicos: promotores de resiliência, fatores de risco e de proteção. *Estudos de Psicologia (Campinas),* 25, 405-416.

Prochaska, J. O. & DiClemente, C. C. (1983). Stages and processes of self-change of smoking: toward an integrative model of change. *Journal of Consulting and Clinical Psychology,* 51(3), 390-395.

Prochaska, J. O. & DiClemente, C. C. (1984). Self change processes, self efficacy and decisional balance across five stages of smoking cessation. *Progress in Clinical and Biological Research,* 156, 131-140.

Prochaska, J. O. & Velicer, W. F. (1997). The transtheoretical model of health behavior change. *American Journal of Health Promotion,* 12(1), 38-48.

Roberts, K. E. C. (2009). *An evaluation of the Expect Respect: Preventing teen dating violence high school program (PhD thesis)*, Ohio University.

Rogers, E. M. (2002). Diffusion of preventive innovations. *Addictive Behaviors,* 27(6), 989-993.

Rolf, J., Masten, A. S., Cicchetti, D., Neuchterlein, K. H. & Weintraub, S. (1990). *Risk and protective factors in the development of psychopathology*. (New York, NY, Cambridge University Press).

Rosenblatt, L. M. (1978). The reader, the text, the poem: The transactional theory of reading and writing, Carbondale, IL: Southern Illinois University Press).

Rosenbluth, B., Whitaker, D. J., Anne Valle, L. & Ball, B. (2011). Integrating strategies for bullying, sexual harassment, and dating violence prevention: The Expect Respect elementary school project. In: D. L. Espelage & S. M. Swearer (Ed) *Bullying in North American schools* (New York, NY, Routledge): 262-272.

Rosenbluth, B., Whitaker, D. J., Sanchez, E. & Valle, L. A. (2004). The Expect Respect project: preventing bullying and sexual harassment in US elementary schools. In: P. K. Smith, D. Pepler & K. Rigby (Ed) *Bullying in schools: how successful can interventions be?* (New York, NY, Cambridge University Press): 211-233.

Rosenstock, I. M. (1974). Historical origins of the health belief model. *Health Education Monographs,* 2(4), 328-335.

Rosenstock, I. M., Strecher, V. J. & Becker, M. H. (1988). Social learning theory and the health belief model. *Health Education Quarterly,* 15(2), 175-183.

Rowe, L. S., Jouriles, E. N. & McDonald, R. (2015). Reducing sexual victimization among adolescent girls: a randomized controlled pilot trial of my voice, my choice. *Behavior Therapy,* 46(3), 315-327.

Rushton, J. P. & Campbell, A. C. (1977). Modeling, vicarious reinforcement and extraversion on blood donating in adults: Immediate and long-term effects. *European Journal of Social Psychology,* 7(3), 297-306.

Sabella, R. A. (1995). *The effectiveness of a developmental guidance unit and self-instruction module about sexual harassment among seventh-grade students (PhD thesis)*, University of Florida.

Sallis, J. F. & Owen, N. (2002). Ecological models of health behavior. In: K. Glanz, B. K. Rimer & F. M. Lewis (Ed) *Health behavior: Theory, research, and practice* (San Francisco, CA, Jossey-Bass). 5.

Sanchez-Jimenez, V., Munoz-Fernandez, N. & Ortega-Rivera, J. (2018). Efficacy evaluation of "Dat-e Adolescence": A dating violence prevention program in Spain. *PLoS ONE,* 13(10), e0205802.

Sargent, K. S., Jouriles, E. N., Rosenfield, D. & McDonald, R. (2017). A high school-based evaluation of TakeCARE, a video bystander program to prevent adolescent relationship violence. *Journal of Youth and Adolescence,* 46(3), 633-643.

Schneider, B. H. (1993). *Children's social competence in context: The contributions of family, school and culture*. (Elmsford, NY, US, Pergamon Press).

Scott, J. & Marshall, G. (2009). *A dictionary of sociology*. (Oxford, UK, Oxford University Press).

Scull, T., Malik, C., Morrison, A. & Keefe, E. (2021). Promoting sexual health in high school: A feasibility study of a web-based media literacy education program. *Journal of Health Communication,* 26(3), 1-14.

Scull, T. M., Kupersmidt, J. B., Malik, C. V. & Morgan-Lopez, A. A. (2018). Using media literacy education for adolescent sexual health promotion in middle school: Randomized control trial of Media Aware. *Journal of Health Communication,* 23(12), 1051-1063.

Silverman, A. B. (2000). *Evaluating the efficacy of the Rhode Island Teen Dating Violence Prevention Program (TDVPP): A process and outcome approach to determining the success of both primary and secondary prevention projects (PhD thesis)*, University of Rhode Island.

Sluzki, C. (1997). *A rede social na prática sistęmica. Alternativas terapęuticas*. (Săo Paulo, Casa do Psicólogo).

Szapocznik, J. & Coatsworth, J. D. (1999). An ecodevelopmental framework for organizing the influences on drug abuse: A developmental model of risk and protection. In: C. Hartel (Ed) *Drug abuse: Origins & interventions* (Washington, DC, American Psychological Association): 331-366.

Taylor, B., Stein, N. & Burden, F. (2010a). The effects of gender violence/ harassment prevention programming in middle schools: a randomized experimental evaluation. *Violence and Victims,* 25(2), 202-223.

Taylor, B., Stein, N., Mack, A. R., Horwood, T. J. & Burden, F. (2008). Experimental evaluation of gender violence/harassment prevention programs in middle schools. (Fairfax, VA, ICF International).

Taylor, B., Stein, N. D., Woods, D. & Mumford, E. (2011). Shifting Boundaries: final report on an experimental evaluation of youth dating violence prevention program in New York City middle schools. Report submitted to the National Institute of Justice. (Washington, DC, Police Executive Research Forum).

Taylor, B. G., Mumford, E. A., Liu, W. & Stein, N. D. (2017). The effects of different saturation levels of the Shifting Boundaries intervention on preventing adolescent relationship abuse and sexual harassment. *Journal of Experimental Criminology,* 13(1), 79-100.

Taylor, B. G., Mumford, E. A. & Stein, N. D. (2015). Effectiveness of "Shifting Boundaries" teen dating violence prevention program for subgroups of middle school students. *Journal of Adolescent Health,* 56(2 Suppl 2), S20-26.

Taylor, B. G., Stein, N. & Burden, F. F. (2010b). Exploring gender differences in dating violence/harassment prevention programming in middle schools: results from a randomized experiment. *Journal of Experimental Criminology,* 6(4), 419-445.

Taylor, B. G., Stein, N. D., Mumford, E. A. & Woods, D. (2013). Shifting Boundaries: an experimental evaluation of a dating violence prevention program in middle schools. *Prevention Science,* 14(1), 64-76.

Walker, L. E. (1979). *The battered woman*. (New York, NY, Harper & Row).

Walker, L. E. A. (1995). *Current perspectives on men who batter women: Implications for intervention and treatment to stop violence against women*. Meeting of the Florida Psychological Association, Key West, FL., Key West, Florida.

Walther, D. J. (1986). Wife abuse prevention: Effects of information on attitudes of high school boys. *Journal of Primary Prevention,* 7(2), 84-90.

Weinstein, N. D. (1989). Effects of personal experience on self-protective behavior. *Psychological Bulletin,* 105(1), 31.

Weissberg, R. P., Durlak, J. A., Domitrovich, C. E. & Gullotta, T. P. (2015). Social and emotional learning: Past, present, and future. In: R. P. Weissberg, J. A. Durlak, C. E. Domitrovich & T. P. Gullotta (Ed) *Handbook of social and emotional learning: Research and practice* (New York, NY, Guilford Press): 3-19.

Whitaker, D. J., Rosenbluth, B., Valle, L. A. & Sanchez, E. (2003). Expect Respect: A school-based intervention to promote awareness and effective responses to bullying and sex harassment. In: D. L. Espelage & S. M. Swearer (Ed) *Bullying in American schools: A social-ecological perspective on prevention and intervention* (Mahwah, NJ, Lawrence Erlbaum Associates Publishers): 327-350.

Wolfe, D. A., Crooks, C., Jaffe, P., Chiodo, D., Hughes, R., Ellis, W., Stitt, L. & Donner, A. (2009). A school-based program to prevent adolescent dating violence: a cluster randomized trial. *Archives of Pediatrics and Adolescent Medicine,* 163(8), 692-699.

Yllo, K. (1993). Through a feminist lens In: Gelles RJ & L. DR (Ed) *Current Controversies in Family Violence* (Newbury Park, CA, Sage).

Yom, Y. H. & Lee, K. E. (2005). Effects of a CD-ROM educational program on sexual knowledge and attitude. *CIN - Computers Informatics Nursing,* 23(4), 214-219.
