# Supplementary material for: School‐based interventions for preventing dating and relationship violence and gender‐based violence: A systematic review and synthesis of theories of change
Source: Rev Educ. 2022 Dec 15;10(3):e3382. doi: 10.1002/rev3.3382 (PMC10116865; doi:10.1002/rev3.3382)
Supplement: Supplementary file 3 — Appendix S3 [file REV3-10-0-s004.docx]

**Supplementary Material 3**

**Theory Synthesis: example coding templates**

Examples of coding templates for the Fourth R intervention and the SHARP intervention completed by two reviewers (XX & XX).

**Reviewer 1**

**Coding template for Fourth R (Wolfe, 2009)**

**Inputs:** Curriculum, Teachers Trained, Supporting Materials, Information for Parents

**Curriculum:** 21-lesson curriculum delivered in 28 hours by teachers. Curriculum comprised of 3 units containing seven 75-minute classes each: (1) personal safety and injury prevention, (2) healthy growth and sexuality, and (3) substance use and abuse.

**Teacher Training**: Teachers attended a 6-hour training workshop on DV and healthy relationships taught by an educator and a psychologist to review the materials and participate in skill-building exercises for engaging youths.

**Supporting Materials:** Lesson plans, video resources, role-play exercises, rubrics, handouts, and “Youth Safe Schools” manual, which describes ways to involve students in school and community violence prevention activities.

**Information for Parents:** Parents received information during grade 9 orientation and from newsletters that describe the topics being taught.

**Intervention goals**

**Primary Objective:** Reduce of Sexual Violence Victimisation and Perpetration (as measured 2 years post content delivery)

**Secondary Objective:** Reductions in related risk behaviours of peer violence, substance use, and unsafe sex.

**Key theoretical concepts**

**Social Learning Theory:** emphasis on skills acquisition

**Diffusion of Innovation**: The Fourth R curriculum enables students to practice peer mentoring, role modelling, and mediation, which allow for peer-to-peer diffusion.

**Bystander Psychology** (Secondary Effect)

**Mechanisms of change**

**Skill Development**: Problem Solving Skills, Development of Positive Strategies for Dealing with Pressure, Conflict Resolution Skills*,* Negotiations, delay, and refusal skills

**Knowledge of IPV:** Knowledge of the role of gender in IPV

**Roleplay:** designed to increase interpersonal and problem-solving skills

**Examples:** Peer and Dating Conflicts

**Outcomes**

**Distal Outcome**: Reduction of DV perpetration, larger effect in boys

**Coding Template for Fourth R (Cissner & Ayoub, 2014)**

Fourth R Curriculum implemented in 10 middle schools in Bronx, New York

**Inputs**: Teachers Trained, Supporting Materials, Curriculum, Parental Handout

**Curriculum:** Modified to 50 minutes 26-session curriculum

**Intervention Goals:**

**Primary Goals:** Reduce sexual harassment/assault, Reduce Dating Violence, Reduction in Youth Violence/ Bullying, Challenge Gender Norms and Stereotypes, Reverse Acceptance of Violence, Challenge Violence Accepting Norms**.**

**Auxiliary Goals:** sexual activity, drug and alcohol use, perceptions of school safety

**Key Theoretical Concepts:** Diffusion of Innovation Theory, Social Learning Theory**,** and (Secondary Effect) Bystander Psychology

**Mechanisms of Change:** Positive Relationships, Interactive and Experiential Learning (Role-Play, Activities)**,** Skills: Assertive Skills, Problem-Solving Skills (Conflict Resolution Skills),

**Outcomes**:

**Proximal:** reduced acceptance of pro-violence beliefs and gender stereotypes

**Distal**: decreased peer violence/bullying perpetration, Reduced Dating Violence among High Risk Students who had already experienced or perpetrated DV at baseline

**Reviewer 2**

**Coding template for Fourth R (Wolfe, 2009)**

**Inputs**: Curriculum, teacher training, information for parents, student-led safe school committees

Fourth R curriculum - comprises three units of seven 75-minute classes each: (i) personal safety and injury prevention, (ii) healthy growth and sexuality, and (iii) substance use and abuse. There were curriculum detailed lesson plans, video resources, role-play exercises and handouts provided for all lessons.

Teachers trained in Fourth R - six-hour training workshop taught by an educator and a psychologist to review the materials of the Fourth R curriculum and participate in skill-building exercises for engaging young people.

Information for parents - four newsletters describe the topics taught and a Year 9 orientation

Student-led ‘safe school committees’ – no detail

Youth Safe School Manual - describes ways to involve students in school and community violence prevention activities, such as guest speakers, field trips, community resources and volunteering.

**Intervention Goals:**

Primary outcomes were reductions in physical dating violence (measured two years post-content delivery)

Secondary outcomes were reductions in related risk behaviours of peer violence, substance use, and unsafe sex (i.e. condom use).

**Key Theoretical Concepts:**

Social learning theory – skills acquisition

**Mechanisms of Change:**

Knowledge – healthy relationships, rights and responsibilities

Skills development e.g. conflict resolution, assertiveness skills, interactive and experiential learning, practice with peers, role-playing

**Outcomes**:

Reduction in dating violence perpetration

**Coding Template for Fourth R (Cissner & Ayoub, 2014)**

**Inputs**: Curriculum, teacher training

Fourth R curriculum – modified to 50 minute, 26 session curriculum

Teacher training – six-hour training session that provided information, hands-on training and resources

**Intervention Goals:**

Primary **-** reduce dating violence victimisation and perpetration, sexual harassment victimisation and perpetration, peer violence/bullying victimisation and perpetration, reduce sexual activity, reduce drug and alcohol use

Secondary – increase school safety, positive beliefs (e.g. anti-fighting/violence, and rejection of gender stereotypes), prosocial behaviours

**Key Theoretical Concepts:**

Social learning theory

Diffusion of innovation theory

Bystander psychology

**Mechanisms of Change:**

Knowledge – healthy relationships

Skills acquisition – conflict resolution skills, interactive and experiential learning e.g. practice peer mentoring, mediation, role playing and use of scenarios

**Outcomes**:

Proximal - reduced acceptance of pro-violence beliefs and gender stereotypes,

Distal – increase school safety, decreased peer violence/bullying victimisation and perpetration, reduced dating violence victimisation and perpetration, reduced sexual harassment victimisation and perpetration

**Reviewer 1**

**Coding template for School Health Center Healthy Adolescent Relationship Program (SHARP) (Miller, 2015)**

**Inputs**

**School Personnel Training (SHC Clinicians and Staff)**: 3-hour training on the SHARP intervention about DRV impact on health and how to introduce the brochure, conduct DRV assessment, and make a warm referral to a victim service advocate (connecting a patient to an advocate via telephone or in person).

**Clinic-Based Assessment:** providers discussed healthy and unhealthy relationships with every patient and gave them several palm-sized brochures on healthy relationships (to further disseminate to the student’s friends). Further intervention components were delivered as needed to patients experiencing DRV: discussing harm reduction strategies and connecting patients to a domestic violence or sexual assault advocate.

**Supporting Materials**: Brochure with DRV Prevention Information for Students, Staff Educational Materials

**Youth-Led Outreach Events**: teams of students implemented 1) a bathroom campaign in both the male and female bathrooms that contains signs that discuss healthy relationships and identify the SHC as a resource and 2) one school-wide activity up to the students’ choosing. (Ex: Valentine’s Day table that gave students the opportunity to write down relationship qualities or actions that are healthy or unhealthy)

**Intervention goals**

**Targeted intervention­** – reach adolescents experiencing DRV

**Early intervention­** – identify adolescents at risk for DRV

**Primary prevention** – Prevent Onset of DRV through healthy relationship education

**Key theoretical concepts**

**Diffusion of Innovation Theory:** both the act of students disseminating the brochures and the peer-to-peer interaction of the youth-led outreach events

**Bystander Behaviour:** providers encouraged students to exhibit bystander behaviours

**Mechanisms of change**

Knowledge, Positive Relationships, Awareness of Services, Self-Efficacy, Bystander Behaviours

**Outcomes**

**Proximal**: Increased recognition of abusive behaviour and increased intention to intervene

**Distal:** Prevention and reduction in dating violence victimisation

**Reviewer 2**

**Coding template for School Health Center Healthy Adolescent Relationship Program (SHARP) (Miller, 2015)**

**Inputs**

School staff training (clinicians and staff at the School Health Centers) - three-hour session on SHARP intervention on how abusive adolescent behaviours (ARA) impacts health, how to introduce a brochure that discusses healthy relationships, conduct an ARA assessment, and make a referral to a victim service advocate.

Clinic-based assessment - SHC staff discuss healthy and unhealthy relationships in each clinical encounter with the brochure provided regardless of reason for visit. Patients are encouraged to take brochures for friends.

Supporting materials – SHARP clinical guidelines, a palm-sized brochure that discusses healthy relationships and how to help a friend, and ARA resources.

School-wide outreach events - each SCH use youth advisory boards to organise events to present ARA information and encourage students to come to SHC. Student leaders present information during lunches and assemblies to encourage students to come to the SHC to learn about ARA.

**Intervention goals**

Primary **–** increased recognition of abusive behaviours, increased intentions to intervene, increased knowledge of ARA resources

Secondary **–** increased self-efficacy to use harm reduction behaviours

**Key theoretical concepts**

Diffusion innovation theory

Bystander behaviour

**Mechanisms of change**

Knowledge, attitudes, self-efficacy, awareness of services

**Outcomes**

Proximal - increased recognition of ARA, increased knowledge of ARA resources and increased intention to intervene

Distal – prevention and reduction of ARA
