# Supplementary material for: School‐based interventions for preventing dating and relationship violence and gender‐based violence: A systematic review and synthesis of theories of change
Source: Rev Educ. 2022 Dec 15;10(3):e3382. doi: 10.1002/rev3.3382 (PMC10116865; doi:10.1002/rev3.3382)
Supplement: Supplementary file 4 — Appendix S4 [file REV3-10-0-s001.docx]

**Supplementary Material 4**

**Sample of Theory of Change Models**

**Second Step**

The SS-SSTP intervention is a social-emotional learning programme that aims to reduce youth violence including peer aggression, peer victimisation, homophobic name-calling, cyber bullying and cyber sexual harassment, sexual violence perpetration and victimisation, and teen dating violence among middle school students.

**Figure 1.** Logic Model of Second Step


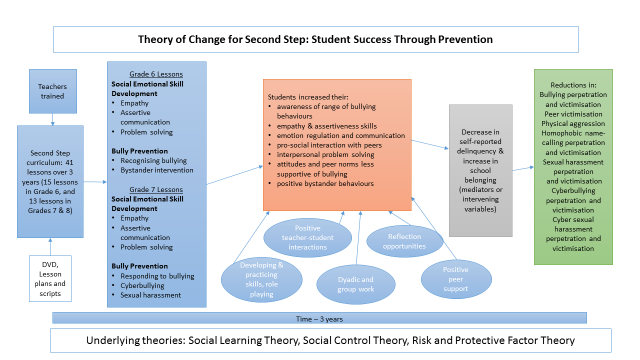


**Theoretical assumptions**

There are several theoretical assumptions underpinning the theory of change for this intervention. Firstly, it draws from the risk and protective framework literature. Risk and protective factor theory suggests that “…problem behaviours are rooted in a common overlapping group of risk and protective factors” (Espelage et al, 2015: 465) and so Second Step targets risk and protective factors linked to aggression, violence and substance use. Secondly, social learning theory informs the design and delivery of the curriculum with its emphasis on skills acquisition; and thirdly, social control theory which posits that self-control is established among young people through opportunities to interact in positive and prosocial ways with individuals and communities, and thereby forming bonds with others and institutions. In the delivery of the curriculum, young people have the opportunity to interact with teachers and other students, promoting positive teacher-students relations.

**Inputs**

The intervention has two key inputs, the training of the teachers who facilitate the delivery of the Second Step curriculum, and the actual Second Step curriculum. These two key inputs are the key themes in the theory of change.

1. Teachers trained in the Second Step curriculum

Teachers receive a three or four-hour training which covers the curriculum and its delivery, and an introduction to child developmental stages related to the skills taught and a background on bullying research. They receive a DVD to support the delivery of lessons with ‘media-rich content’ including topic-focused interviews with students and a video demonstrations of skills. Teachers are also provided with suggestions for connecting lessons to events of the day and to revisit skills as conflicts occur outside the classroom. At the end of lessons, teachers complete online implementation logs on student engagement and components of lesson completed.

2. Second Step Curriculum

The curriculum comprises 15 lessons at Grade 6 and 13 lessons each at Grades 7 and 8. The lessons are delivered in one 50-minute or two 25-minute classroom sessions, taught weekly or semi-weekly throughout the year. The curriculum content has direct instruction in risk and protective factors linked to aggression and violence, including empathy training, emotion regulation, communication skills and problem solving strategies (Espelage et al, 2015: 53). The curriculum also indirectly targets school violence by targeting the peer context for bullying by

…expanding students’ awareness of the full range of bullying behaviors, increasing perspective taking skills and empathy for students who are bullied, educating students on their influence and responsibility as bystanders, and education and practice on the appropriate, positive responses that students can use as bystanders to remove peer support for bullying (Espelage et al, 2015: 54).

Students are taught and practice a range of bystander behaviours from refusing to provide an audience to directly intervening to stop bullying. The programme is designed to change the peer context, removing the bystander support, so important for bullying and other violent behaviours.

In the sixth-grade there are two lessons that focus specifically on bullying – only introduced after students have been exposed to empathy and communication training – and in Grade 7, students review the components of bullying and how to respond. They are encouraged to learn ways on how to intervene to help other as ‘allies’. They also learn how sexual harassment differs from flirting and learn assertive skills to refuse sexual harassment.

**Mechanisms of Change**

There are a number of mechanisms through which Second Step is expected to achieve its outcomes and these are underpinned by the important themes of skills acquisition and developing prosocial relationships among peers. Lessons are skills-based and students receive coaching on performance and suggestions for improvement. All of the activities surrounding the delivery of the curriculum emphasise skills development: “[H]omework assignments, extension activities, academic integration lessons, and videos all serve to reinforce each skill and promote skills acquisition” (Espelage et al, 2015: 466). The use of group and collaborative work encourages students to practice skills in a supportive environment. The emphasis of the delivery is on student interaction – both with teachers and with other students:

Lessons are highly interactive, incorporating small-group discussions and activities, dyadic exercises, whole-class instruction, and individual work (Espelage et al, 2013:181).

This approach to learning provides opportunities for positive teacher-student interactions and the strengthening of relationships.

**Outcomes**

The distal outcomes includes face-to-face bullying, cyberbullying, homophobic name-calling, and sexual harassment perpetration. The authors hypothesise that Second Step indirectly effects these outcomes through the intervening variables of self-reported delinquency (e.g. skipping school, cheating and shoplifting) and sense of school belonging (Espelage et al, 2017).

…it is also quite plausible that there is a cascade effect in which Second Step is more likely to turn off disruptive and oppositional behaviors that are perhaps less ecologically complicated, which in return reduces more complex forms of misbehaviour, such as bullying (Espelage et al, 2015: 467).

**Fourth R**

The Fourth R: Skills for Youth Relationships is an intervention that integrates dating violence prevention with lessons on healthy relationships, sexual health and substance use. It aims to develop relationship skills to promote safer decision making with peers and dating partners.

**Figure 2.** Logic Model of Fourth R


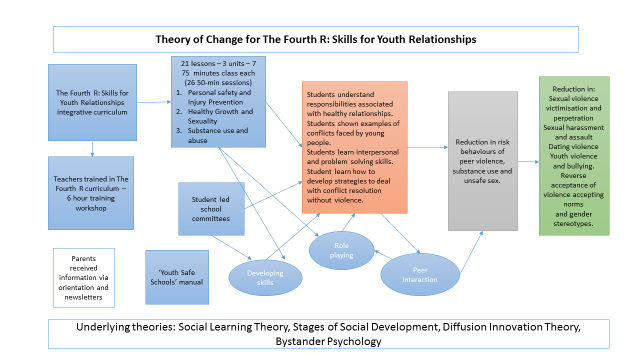


**Intervention Inputs**

The intervention has two main inputs: the training of the teachers who deliver the Fourth R curriculum as part of the Health and Physical Education curriculum in the classroom; and the actual Fourth R curriculum. These two key inputs are the two main themes in the theory of change. Additional inputs included information for parents and student led ‘safe school committees’.

1. Teachers trained in the Fourth R curriculum

Teachers are the key facilitators of the intervention. The initial input is the teacher training in dating violence and healthy relationships. Teachers receive a six-hour training workshop, taught by an educator and a psychologist, to review the materials of the Fourth R curriculum and participate in skill-building exercises for engaging young people. Teachers receive detailed lesson plans, training videos, role-playing demonstrations and received individual feedback. They also have experience of implementing the curriculum for one semester before the trial, increasing their familiarity with the content. That the intervention could be delivered by teachers in receipt of supplementary training was perceived as “…a more sustainable and less expensive strategy compared with programs delivered by non-teachers”(Wolfe et al, 2009: 693).

2. The Fourth R Curriculum

The Fourth R curriculum is taught as part of the regular curriculum without requirements for additional class time or scheduling. This was regarded as a particular strength of the intervention:

The focus on embedding the program into curriculum that meets the guidelines for mandatory classes in high schools provides a vehicle for widespread dissemination and sustainability far beyond that which can be achieved by add-on programs (Wolfe et al, 2009: 698).

The curriculum comprises three units of seven 75-minute classes each; (i) personal safety and injury prevention, (ii) healthy growth and sexuality, and (iii) substance use and abuse. In the Bronx adaptation of Fourth R the curriculum comprises 26 50-minute sessions. The curriculum topics are not addressed independently but the theme of healthy, non-violent relationships are woven throughout the units to increase generalisation across risk situations and behaviours. This has the additional benefit of eliminating “the need for multiple programs targeting overlapping behaviors” (Cissner & Ayoub 2014: ix). The curriculum adopts a gender approach to dating violence highlighting gender specific patterns and factors and matching activities accordingly. Activities and exercises were tailored for boys and girls to maximise relevance and minimise defensiveness in class.

There were detailed lesson plans, video resources, role-play exercises and handouts provided for all lessons. There was ‘extensive skill development’ to enable young people to develop strategies for dealing with pressures and resolving conflict without resorting to violence.

3. Information for Parents

Parents receive information from a Year 9 orientation and from four newsletters that describe the topics taught.

4. Student-led ‘safe school committees’

No detail on these but are mentioned as one of the inputs.

**Mechanisms of Change**

There are several mechanisms through which the Fourth R is expected to achieve the desired outcomes of the intervention. A key theme within the mechanisms of change is the ‘extensive skill development’ (Wolfe et al, 2019: 693) aiming to give young people positive strategies for dealing with pressures and resolving conflict without abuse or conflict such as negotiation, delay and refusal skills. This is facilitated by giving students detailed examples of conflicts experienced by young people which include peer bullying and dating conflicts. A second theme is the emphasis on interactive learning to engage students:

[i]t also makes extensive use of scenarios and role-playing, with the goal of increasing students’ problem-solving skills and providing opportunities to practice new skills…Role-play is also used in relationship violence scenarios. A wide variety of activities and exercises allow students to engage individually, in pairs, as small groups, or as a class (Cissner & Ayoub, 2014: 33)

A sub-theme is the diffusion of the Fourth R curriculum messages across the schools via peer-to-peer contact and or teacher-student contact (Cissner & Ayoub, 2014: 30).

**Outcomes**

The primary outcomes are reductions in physical dating, sexual harassment/assault, youth violence/bullying, and reversals in acceptance of gender norms and stereotypes, violence and acceptance norms. Secondary outcomes are reductions in related risk behaviours of peer violence, substance use, and unsafe sex (i.e. condom use).

**TakeCARE**

By using a brief video bystander programme, TakeCARE aims to promote self-efficacy among high school students for increased engagement in bystander behaviour (with the broader goal of reducing relationship violence). TakeCARE aims to ensure that students develop confidence that they can do something to help in risky situation or in situations where violence has occurred. TakeCARE is an acronym for students to remember that an effective bystander is: C—Confident that they can help their friend avoid risky situations, A—Aware that their friends could get hurt in these kinds of situations, R—Responsible for helping, and E—Effective in how they help.

**Figure 3.** Logic model of TakeCARE

**
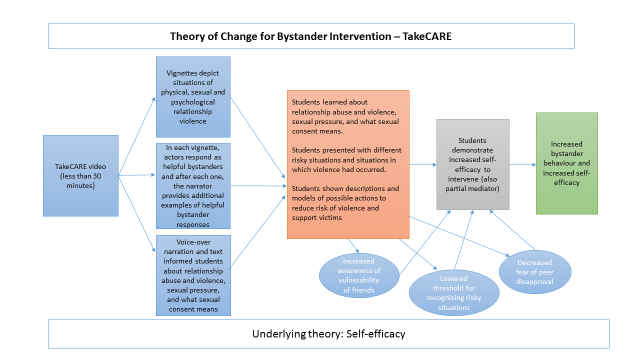
**

**Intervention Inputs**

The video is the key input of the TakeCARE intervention, delivered to the students by high school staff in the classroom. There are no inputs around teacher or staff training. This, according to the authors, makes TakeCARE an ‘inexpensive’ and ‘easily scalable’ intervention, in contrast to those bystander interventions that are delivered by trained facilitators which can be ‘cost-prohibitive for schools desiring campus-wide dissemination’ (Sargent et al, 2017: 634).

…the time and training demands of these programs [e.g. Green Dot] put them beyond the reach of many schools and school districts (Jouriles et al, 2019: 4).

**Mechanisms of change**

There are two mechanisms through which the TakeCARE video is expected to achieve the intervention’s outcomes. A key theme is the importance of building self-efficacy and showing students how they can be ‘more than just a bystander’ with three vignettes involving dating violence - one depicts a potentially violent situation, another an actively violent situation, and the third is about support after a risky situation has occurred. In each vignette, the actors respond as helpful bystanders to prevent a negative situation arising, de-escalate a situation, and support a friend after a risky situation has already happened. After each vignette, the narrator provides additional examples of helpful bystander responses that could have been provided. The authors emphasise that TakeCARE offers students ‘concrete examples’ of what they can say or do (Jouriles et al, 2019: 4); ‘not knowing what to do’ was identified by the students as the biggest obstacle to responsive bystander action by students in the intervention development meetings (Sargent et al, 2017: 636). Knowledge is the other mechanism and the video conveyed information to students on identifying abusive dating relationships, the definition of, and issues around consent, to sexual activity, and providing support to someone who discloses that non-consensual or distressing consensual has already occurred.

Jourilles et al (2019: 12) suggests that additional mechanisms may have been at play and “…might include increased awareness of the vulnerability of friends to violence, a lowered threshold for recognizing risky situations and therefore intervening, or a decreased fear of peer disapproval for saying or doing something to help protect friends.”

**Outcomes**

The TakeCARE intervention is expected to achieve the following outcomes: students are expected to increase their self-efficacy and responsibility which ultimately, should lead to increased bystander behaviour. Jourilles et al (2019: 12) acknowledge that while this study focused on bystander behaviour as an outcome, the ultimate goal of bystander programmes is to reduce rates of campus violence.
